# Supplementary material for: Anticipated impacts of Brexit scenarios on UK food prices and implications for policies on poverty and health: a structured expert judgement approach
Source: BMJ Open. 2020 Mar 3;10(3):e032376. doi: 10.1136/bmjopen-2019-032376 (PMC7059525; doi:10.1136/bmjopen-2019-032376)
Supplement: Supplementary data [file bmjopen-2019-032376supp001.pdf]

Supplementary Information – File S1

Barons, M. and Aspinall, W. **Anticipated impacts of Brexit scenarios on UK food prices and implications for policies on poverty and health: a structured expert judgement approach**

Summary of file contents

This file serves as a document of record for several details and aspects of a research Workshop that was convened in July 2018 to discuss and consider the potential impact of two alternative Brexit scenarios on food prices. The contents of this file relate to material that is too extensive to include in the main paper, and also cover details of the structured elicitation that was adopted to furnish the price change estimates reported in the paper. Other supporting information may be found in Supplementary File S2.

The objective of the Workshop was to develop estimates and quantify associated uncertainties in respect of several key variables that can influence future food prices, in order to provide decision support for UK policymakers concerned with food insecurity, public reliance on food banks, impacts on public health and related issues.

Contents

Supplementary Information – File S1 1
  Summary of file contents 1
Why the workshop was held 2
Structured Expert Judgement Elicitation 2
  Experts' scores 3
  Findings on target item food price changes 5
Discussion of results 18
  Target item comments 18
Appendix S1.1 Target items questionnaire 21
Appendix S1.2 Workshop Invitation 27
Appendix S1.3 Briefing note sent to participants 29

Why the workshop was held

This workshop was held as part of research aimed at providing decision support for policymakers concerned with ameliorating UK household food security (see also Appendix S2.2 Workshop Invitation). It is well known that critical factors in the pathway top household food insecurity are equivalised household disposable income and the cost of food.

Food prices in the UK are monitored as part of the Consumer Prices Index (CPI), the inflation measure used in the government's target for inflation. Consumer price inflation is the rate at which the prices of goods and services bought by households rise or fall. A convenient way of thinking about this is to imagine a very large "shopping basket" containing those goods and services bought by households. As the prices of the various items in the basket change over time, so does the total cost of the basket. Movements in consumer price inflation indices represent the changing cost of the shopping basket.

Although kept constant within year, the contents of the consumer price inflation basket of goods and services and their associated expenditure weights are updated annually. This is important in helping to avoid potential biases that might otherwise develop over time – for example, due to the development of entirely new goods and services, or the tendency for consumers to move away from buying goods and services that have risen relatively rapidly in price and to goods and services whose prices have fallen.

Changes to the items and their associated item weights are introduced in the February index each year, but prices are collected for both old and new items in January. This means that the figures for each year can be "chain linked" together to form a long-run price index spanning many years. In other words, price changes between December and January are based on the old basket and weights, while price changes between January and February, and beyond, are based on the new basket and weights. This procedure ensures that the annual changes to the basket and weights do not introduce a discontinuity in prices as measured by the indices.

(see: https://www.ons.gov.uk/economy/inflationandpriceindices/articles/ukconsumerpriceinflationbasketofgoodsandservices/2018)

Food and non-alcoholic beverages comprise one element of the UK CPI basket. This element is divided into 10 broad categories, with several sub-categories in each. The 10 categories are: Bread and cereals; Meat; Fish; Milk, cheese and eggs, Oils and fats; Fruit; Vegetables including potatoes and tubers; Sugar, jam, syrups, chocolate and confectionery; Coffee, tea and cocoa; Mineral waters, soft drinks and juices. In the workshop in July 2018 experts were asked to estimate the changes in prices of each of these food categories under a range of Brexit-related scenarios which were then expected to be implemented in March 2019; Brexit did not happen at that time.

Structured Expert Judgement Elicitation

When policymakers wish to make evidence-based decisions, they often take evidence from relevant experts. There are several established, tested structured elicitation protocols which aim to ameliorate biases and other frailties associated with gathering scientific advice.

In the present case, Cooke's method (Cooke, R.M. 1991 *Experts in Uncertainty*, OUP.), sometimes called the Classical Model, was adopted as the framework for eliciting and pooling experts' judgments about future food prices (a briefing note for participants is reproduced in Appendix S2.3).

It is a mathematical formulation for combining the views of a panel of experts, with their expressed uncertainty distributions on future food prices weighted by their performance scores from a set of calibration questions designed to assess an individual's statistical accuracy and informativeness. In the Classical Model, statistical accuracy is measured as the P-value or probability with which one would falsely reject the hypotheses that an expert's probability assessments were statistically accurate. A low value (near zero) means it is very unlikely that the discrepancy between an expert's probability statements and observed outcomes should arise by chance. Informativeness is measured as Shannon relative information with respect to an analyst-supplied background measure. Shannon relative information is used because it is scale invariant, tail insensitive, slow, and familiar.

Solutions to the elicitation of target item food price changes are presented as Performance Weighted (PW) combinations of the panel members' judgments and, for context, these PW solutions are usually compared with Equal Weights (EW) combinations, when there is no differential weighting of experts.

There are two flavours of performance weights: (1) *global weights*, which compute an expert's *combined score* as the product of his/her P-value (i.e. statistical accuracy score on the seeds) and the average of the expert's global information score over all seed and target variables; (2) *item weights*, which are computed specific to each variable/item, being the product, item-by-item, of the P-value (which is always global over all seeds) and the expert's information score for each separate variable/item.

Item weighting allows an expert, in effect, to up/down weight him/herself per item if (s)he thinks (s)he knows more/less about that item. If experts perform this up/down weighting well, item weights will out-perform global weights which, by definition, are more generic/non-specific. Hence, in the absence of contra-indications, we prefer *item weights* solutions to *global weights* as they offer more informative solutions, per item.

Experts' scores

In the present elicitation, ten participants completed the calibration exercise, and their statistical accuracy and information scores are shown on the following table.

Table S2. 1 Panel experts' scores from Classical Model calibration (see text)

Expert	Statistical accuracy (P-value)	Information score
1	0.0750	1.24
2	0.0171	1.25
3	0.0063	1.79
4	0.0028	0.97
5	0.0003	1.82
6	5.6E-05	1.40
7	1.9E-06	1.98
8	1.9E-06	1.97
9	2.8E-09	1.82
10	1.3E-10	2.24

The experts are ordered, anonymously, in terms of their P-values scores; expert labels, 1, 2 … 10 are not related to individual identities, nor do these identifiers correspond to labels used elsewhere in

this document. Three of the ten experts achieved scores indicating that they provided informative and statistically accurate judgments over the set of calibration questions.

The corresponding scores are shown in Figure S2.1:

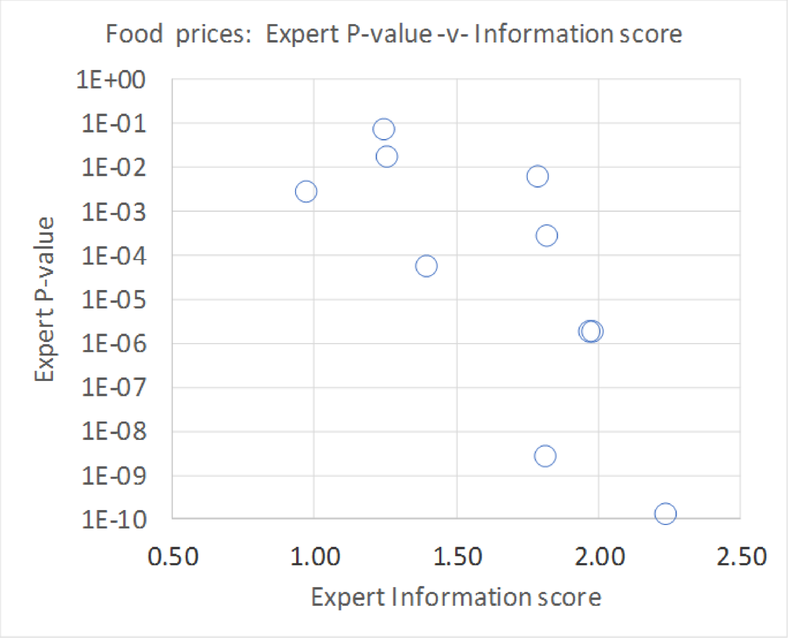

Figure S2. 1 Relationships of experts' statistical accuracy (P-value) scores to their Information scores (see text for explanation)

This plot shows that the food prices group evinced the usual trait of generally individual low P-values due to unduly high Information scores; this profile is encountered with nearly every professional expert group elicitation conducted with the Classical Model.

In short, there is a negative correlation between individuals' information scores (on seed items) and their corresponding P-values (statistical accuracy). Thus, several of the group recorded low P-values mainly because their uncertainty spreads were systematically too narrow; i.e. many demonstrated a tendency to express judgments that result in a high Information score but, because of this, they failed to capture enough realization values in the set to gain a high statistical accuracy score overall.

With the Classical Model providing a formal mathematical basis for expert scoring, this sort of expert group profile is unexceptional in structured elicitations that focus on uncertainty quantification for parameters and variables that are difficult to constrain otherwise, by statistical analysis, modelling or from theoretical considerations.

Barons MJ, Aspinall W. *BMJ Open* 2020; 10:e032376. doi: 10.1136/bmjopen-2019-032376

Findings on target item food price changes

The following range graphs record the median and 90% credible interval spreads provided by individual experts for each target question, together with the resulting Equal Weights ('EqWts') and Performance Weights ('PerfWts') solutions. The questions that were set before the participants for their judgments are reproduced in Appendix S1.1.

Subject matter: Changes in food prices with and without an agreed BREXIT trade deal (NB NO BREXIT in the plot title means no deal = hard BREXIT; agreed BREXIT means tade deals have been negotiated)

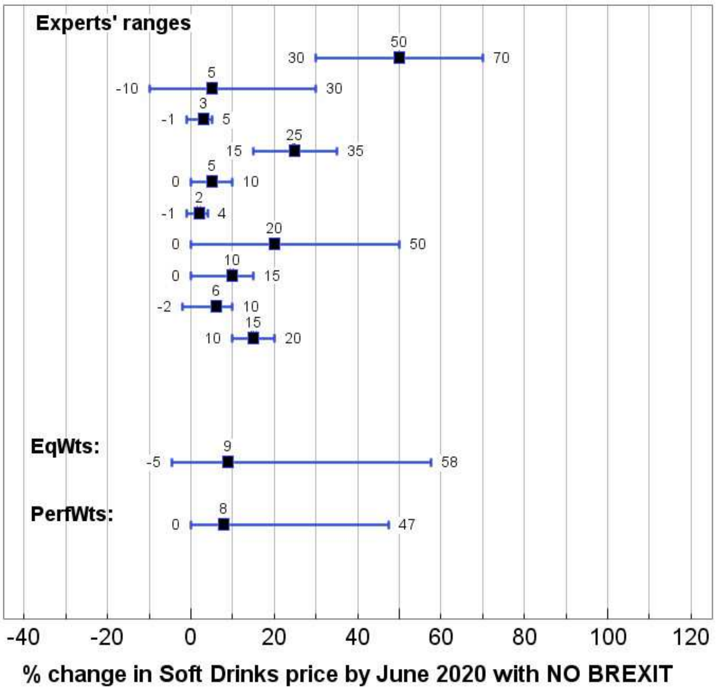

Barons MJ, Aspinall W. *BMJ Open* 2020; 10:e032376. doi: 10.1136/bmjopen-2019-032376

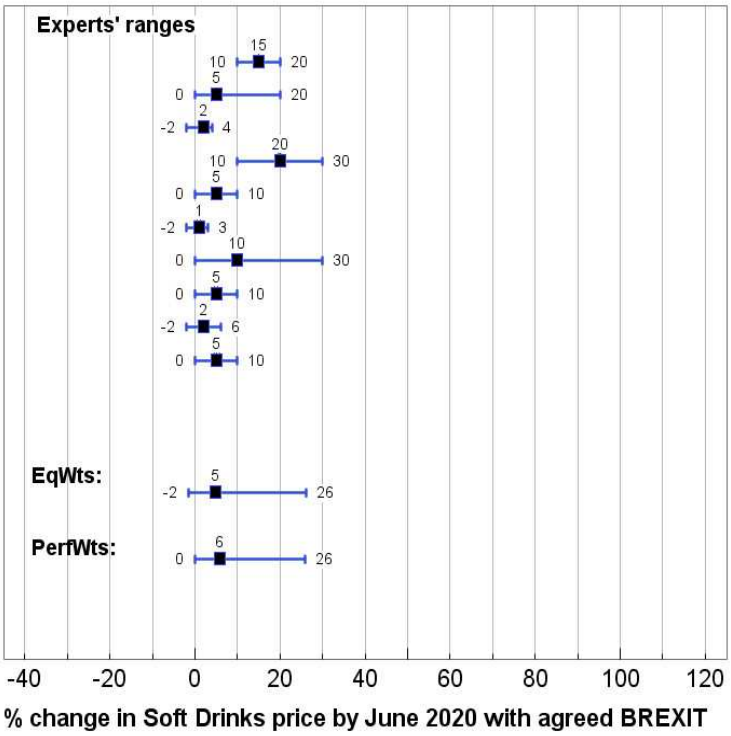

Barons MJ, Aspinall W. *BMJ Open* 2020; 10:e032376. doi: 10.1136/bmjopen-2019-032376

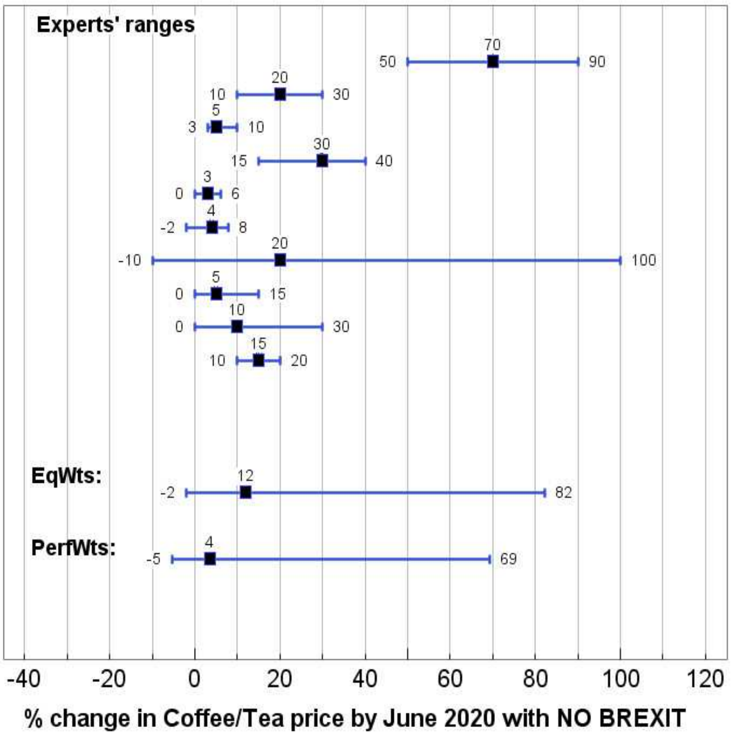

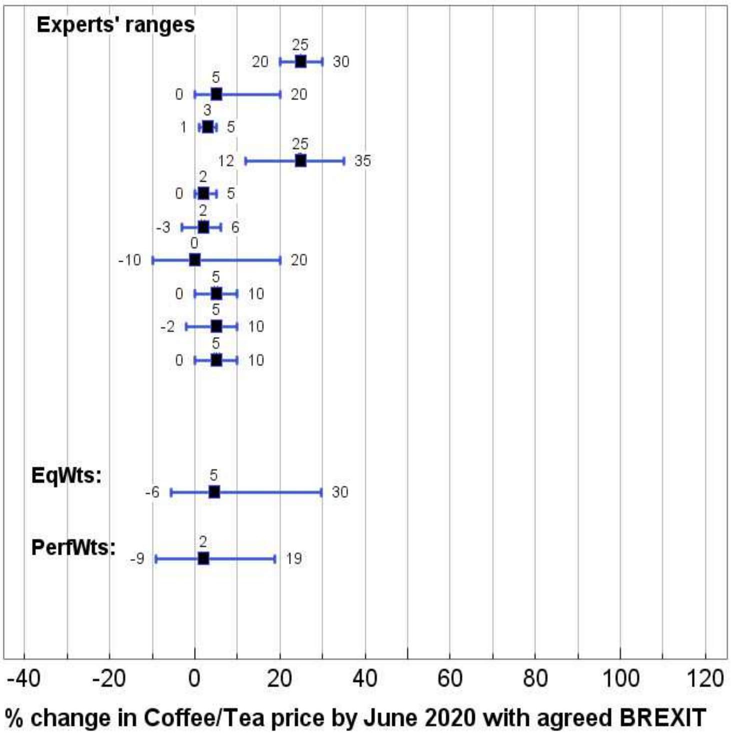

Barons MJ, Aspinall W. *BMJ Open* 2020; 10:e032376. doi: 10.1136/bmjopen-2019-032376

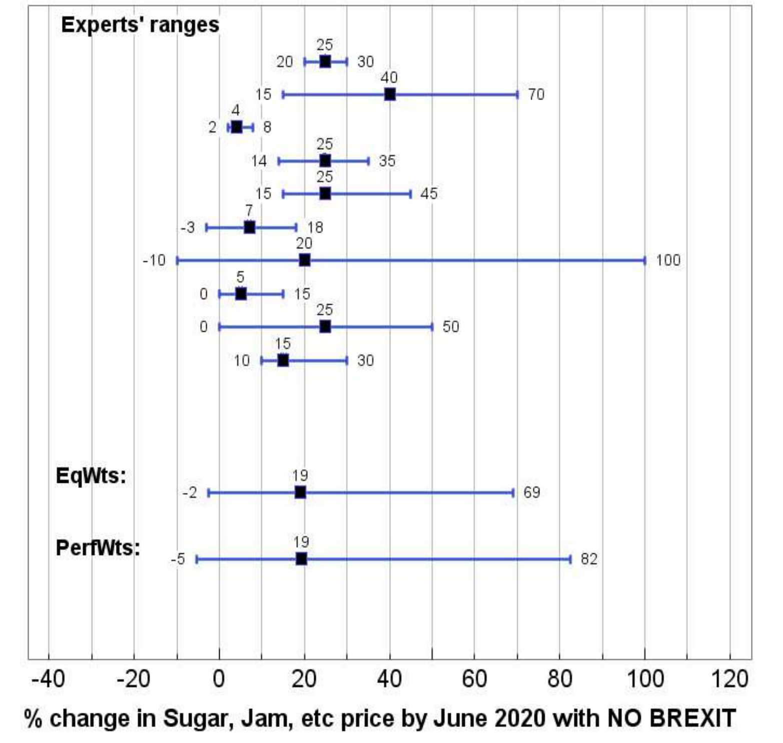
Experts' ranges
25
20 30
40
15 70
4
2 8
25
14 35
25
15 45
7
-3 18
20
-10 100
5
0 15
25
0 50
15
10 30
EqWts:
19
-2 69
PerfWts:
19
-5 82
-40 -20 0 20 40 60 80 100 120
% change in Sugar, Jam, etc price by June 2020 with NO BREXIT

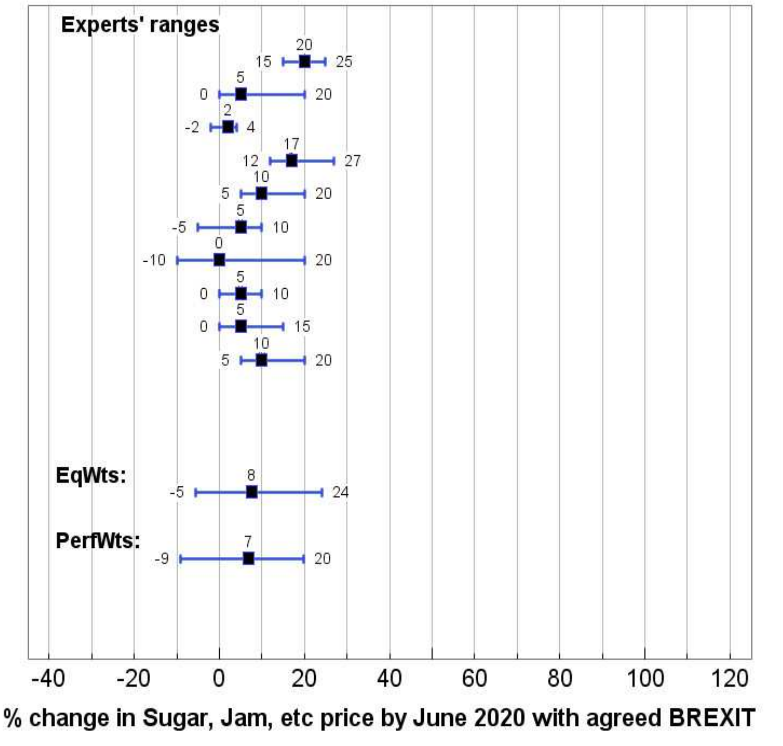
Experts' ranges
20
15 25
5
0 20
2
-2 4
17
12 27
10
5 20
5
-5 10
0
-10 20
5
0 10
5
0 15
10
5 20
EqWts:
8
-5 24
PerfWts:
7
-9 20
-40 -20 0 20 40 60 80 100 120
% change in Sugar, Jam, etc price by June 2020 with agreed BREXIT

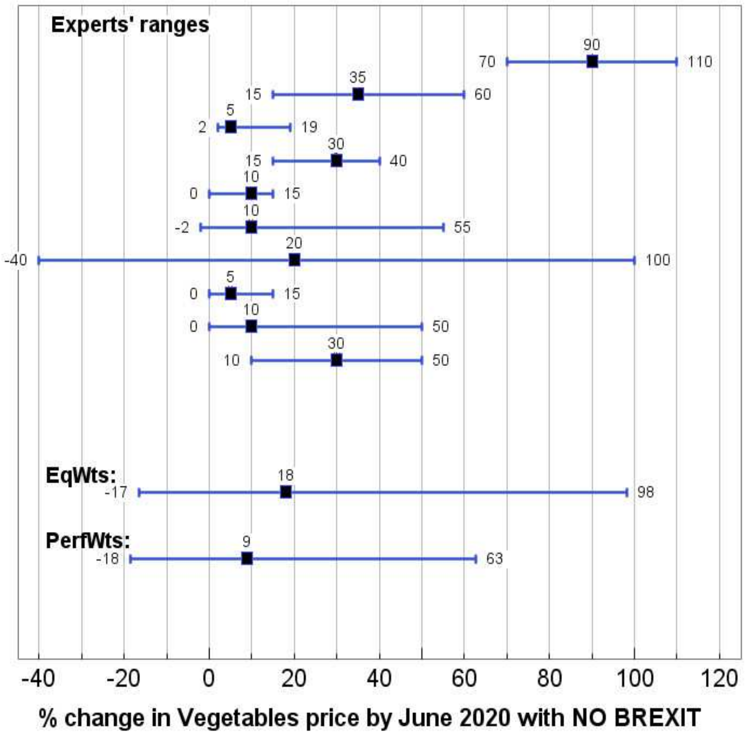

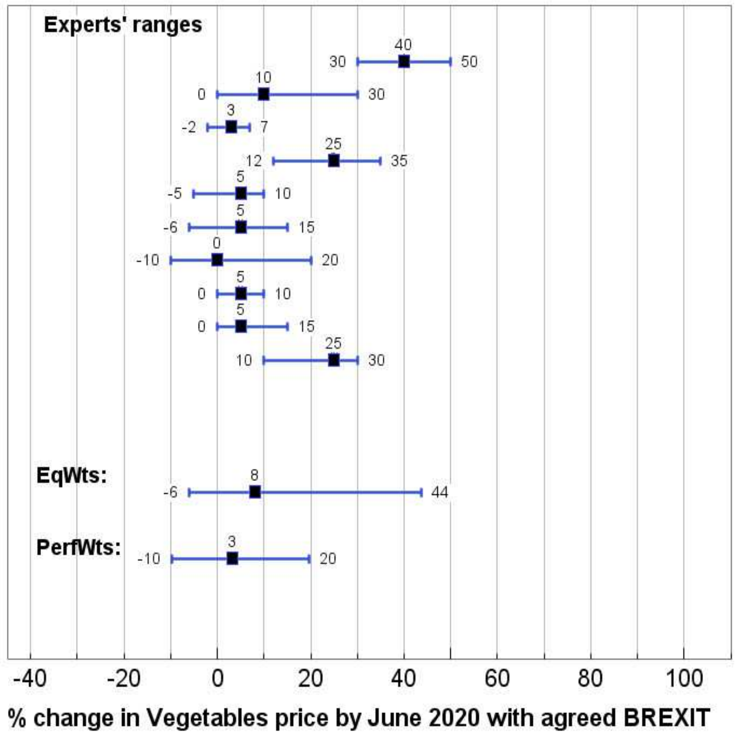

Barons MJ, Aspinall W. *BMJ Open* 2020; 10:e032376. doi: 10.1136/bmjopen-2019-032376

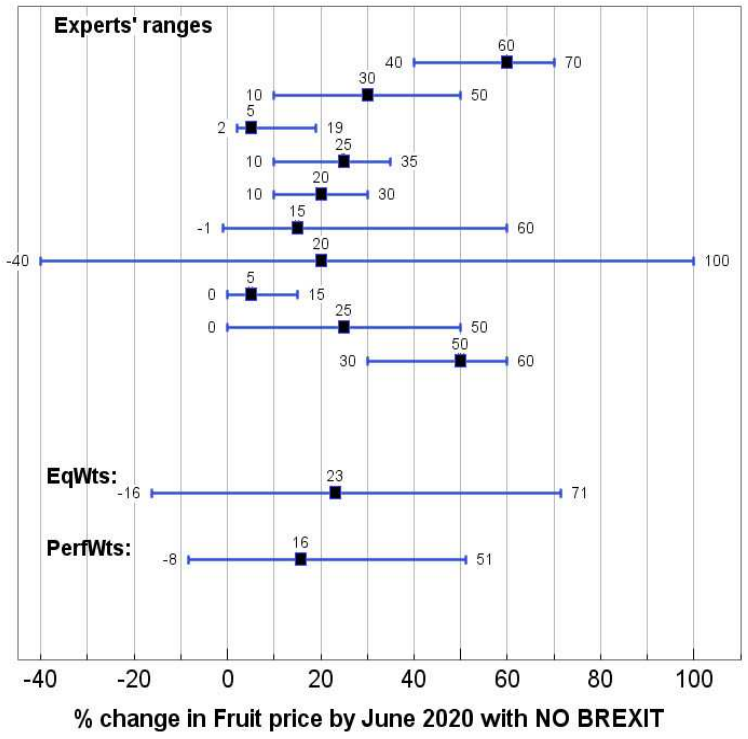
Experts' ranges
60
40 70
30
10 50
5
2 19
25
10 35
20
10 30
15
-1 60
20
-40 100
5
0 15
25
0 50
50
30 60
EqWts:
23
-16 71
PerfWts:
16
-8 51
-40 -20 0 20 40 60 80 100
% change in Fruit price by June 2020 with NO BREXIT

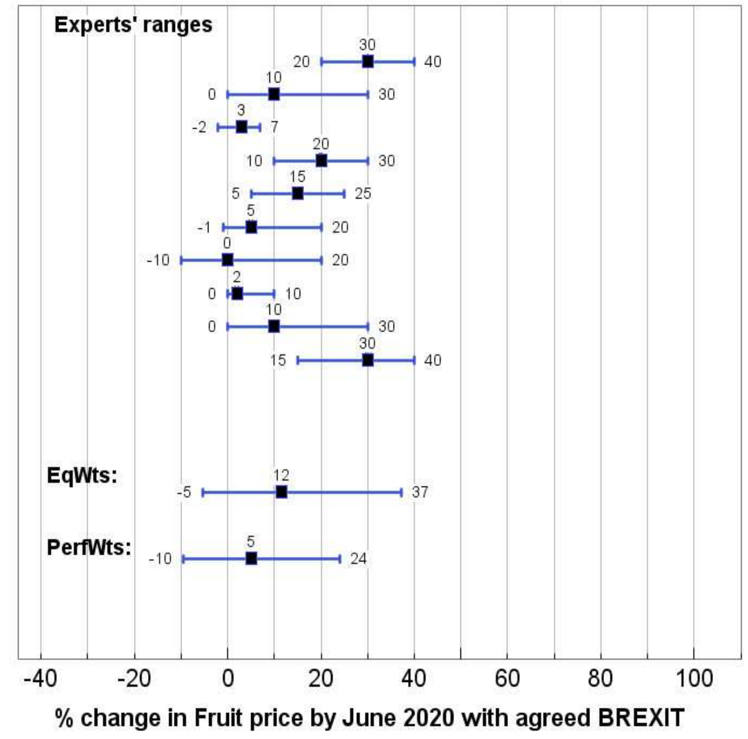
Experts' ranges
30
20 40
10
0 30
3
-2 7
20
10 30
15
5 25
5
-1 20
0
-10 20
2
0 10
10
0 30
30
15 40
EqWts:
12
-5 37
PerfWts:
5
-10 24
-40 -20 0 20 40 60 80 100
% change in Fruit price by June 2020 with agreed BREXIT

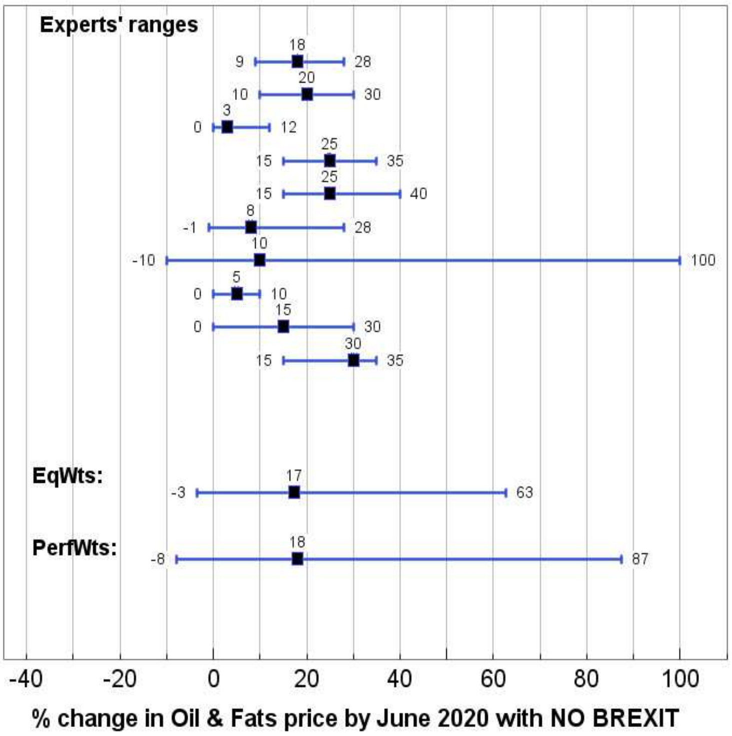
Experts' ranges
18
9 28
20
10 30
3
0 12
25
15 35
25
15 40
8
-1 28
10
-10 100
5
0 10
15
0 30
30
15 35
EqWts:
17
-3 63
PerfWts:
18
-8 87
-40 -20 0 20 40 60 80 100
% change in Oil & Fats price by June 2020 with NO BREXIT

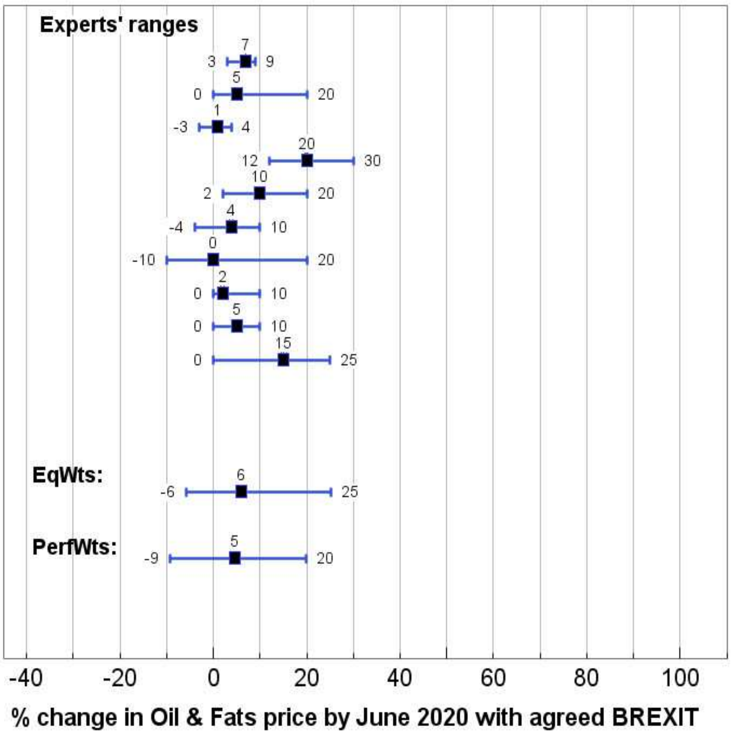
Experts' ranges
7
3 9
5
0 20
1
-3 4
20
12 30
10
2 20
4
-4 10
0
-10 20
2
0 10
5
0 10
15
0 25
EqWts:
6
-6 25
PerfWts:
5
-9 20
-40 -20 0 20 40 60 80 100
% change in Oil & Fats price by June 2020 with agreed BREXIT

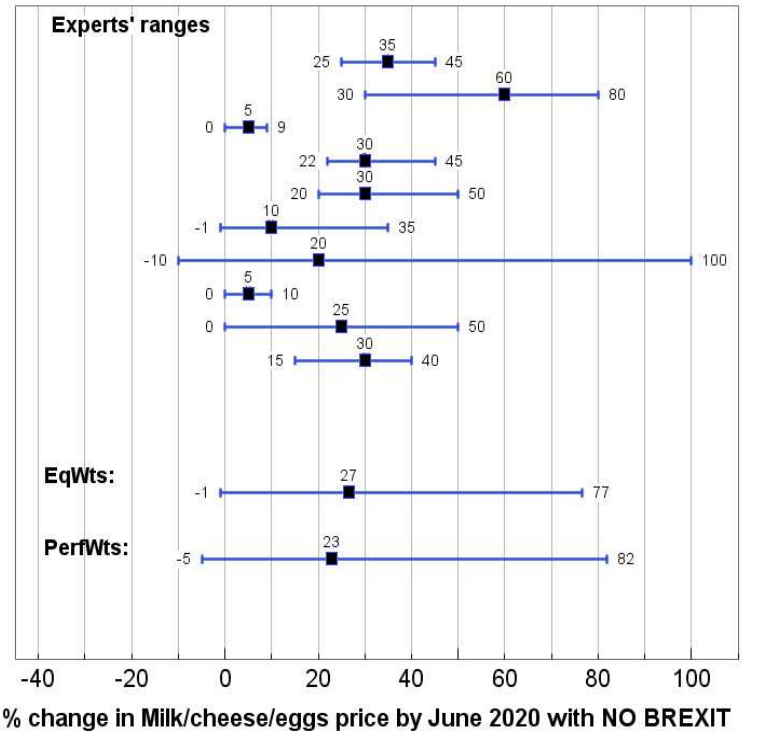
Experts' ranges
25 35 45
30 60 80
0 5 9
22 30 45
20 30 50
-1 10 35
-10 20 100
0 5 10
0 25 50
15 30 40
EqWts:
-1 27 77
PerfWts:
-5 23 82
-40 -20 0 20 40 60 80 100
% change in Milk/cheese/eggs price by June 2020 with NO BREXIT

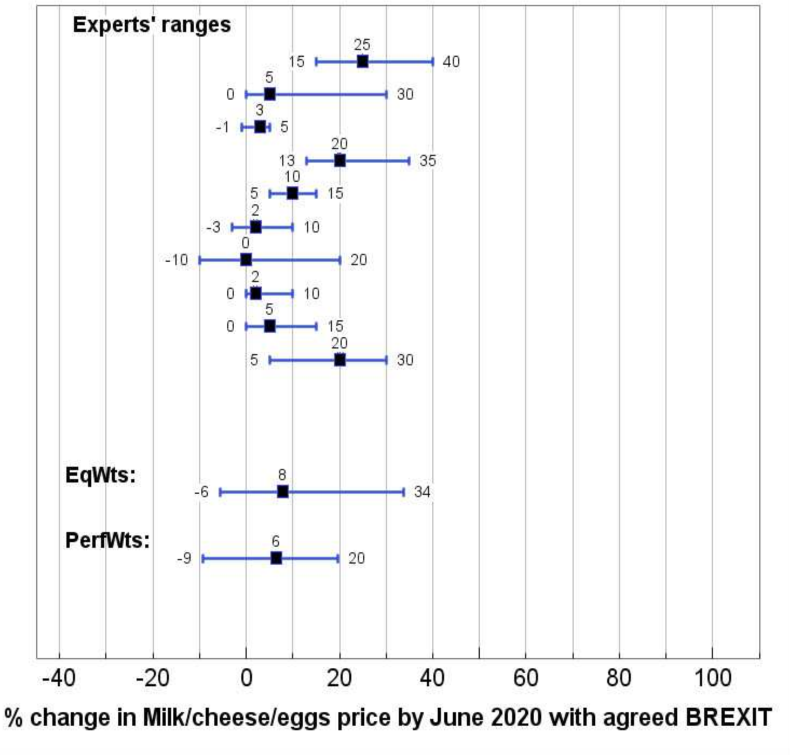
Experts' ranges
15 25 40
0 5 30
-1 3 5
13 20 35
5 10 15
-3 2 10
-10 0 20
0 2 10
0 5 15
5 20 30
EqWts:
-6 8 34
PerfWts:
-9 6 20
-40 -20 0 20 40 60 80 100
% change in Milk/cheese/eggs price by June 2020 with agreed BREXIT

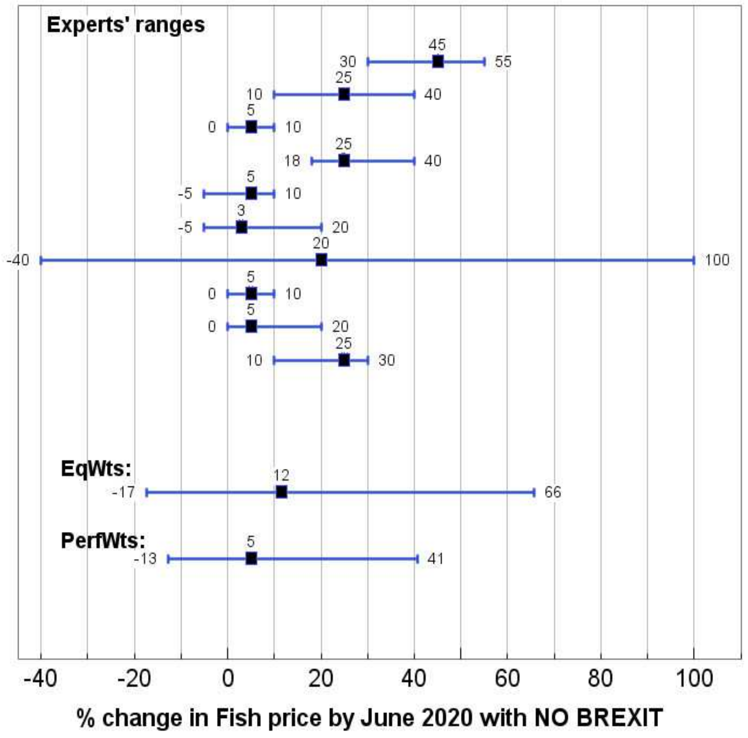
Experts' ranges
45
30 55
25
10 40
5
0 10
25
18 40
5
-5 10
3
-5 20
20
-40 100
5
0 10
5
0 20
25
10 30
EqWts:
12
-17 66
PerfWts:
5
-13 41
-40 -20 0 20 40 60 80 100
% change in Fish price by June 2020 with NO BREXIT

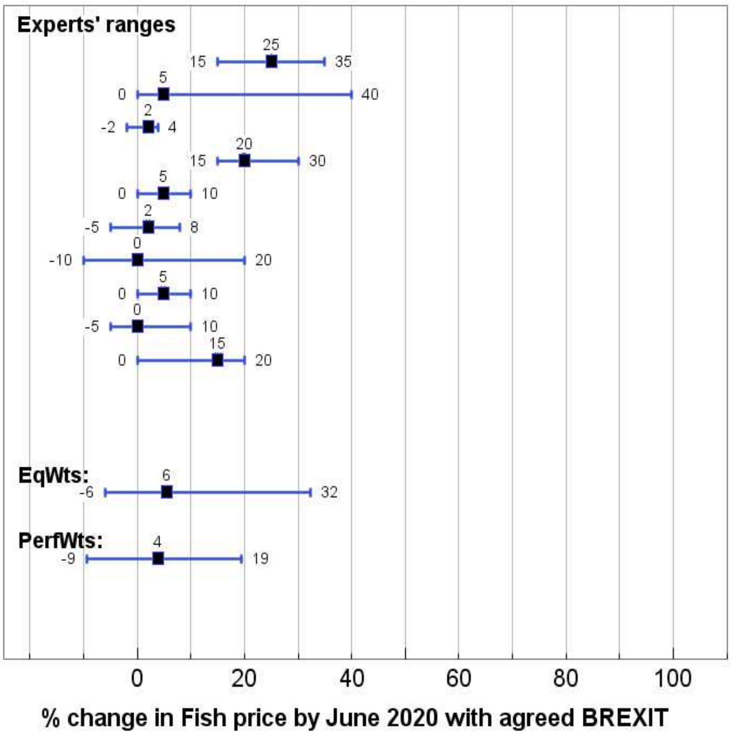
Experts' ranges
25
15 35
5
0 40
2
-2 4
20
15 30
5
0 10
2
-5 8
0
-10 20
5
0 10
0
-5 10
15
0 20
EqWts:
6
-6 32
PerfWts:
4
-9 19
0 20 40 60 80 100
% change in Fish price by June 2020 with agreed BREXIT

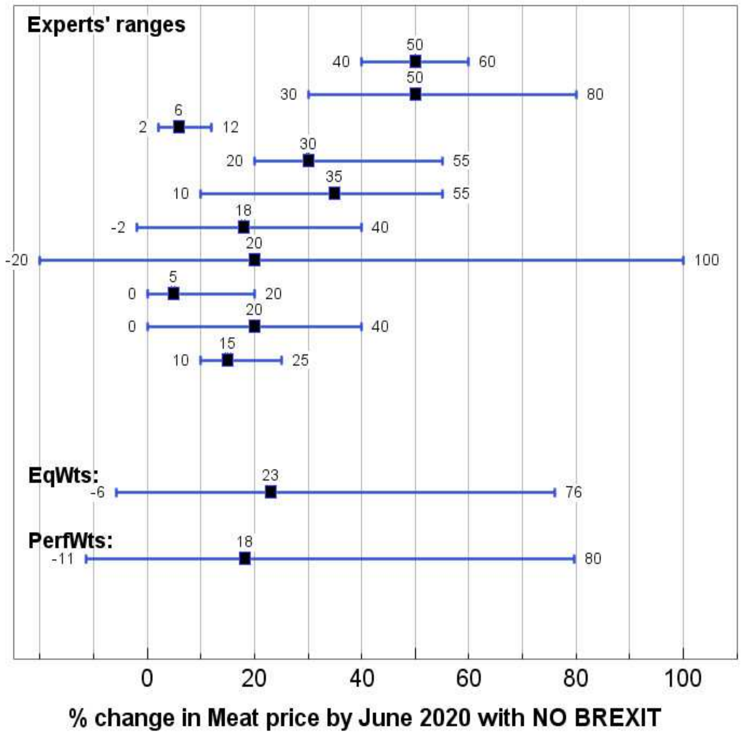

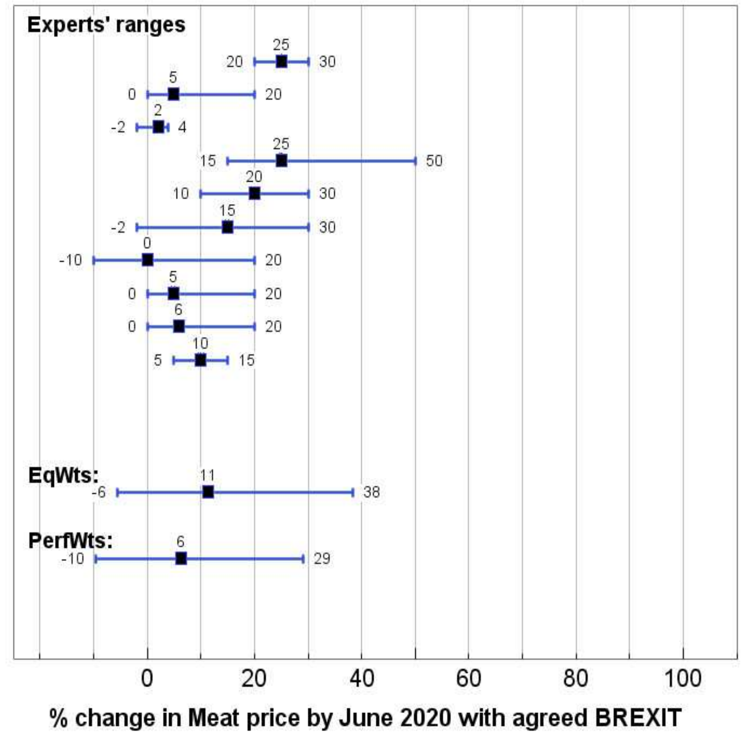

Barons MJ, Aspinall W. *BMJ Open* 2020; 10:e032376. doi: 10.1136/bmjopen-2019-032376

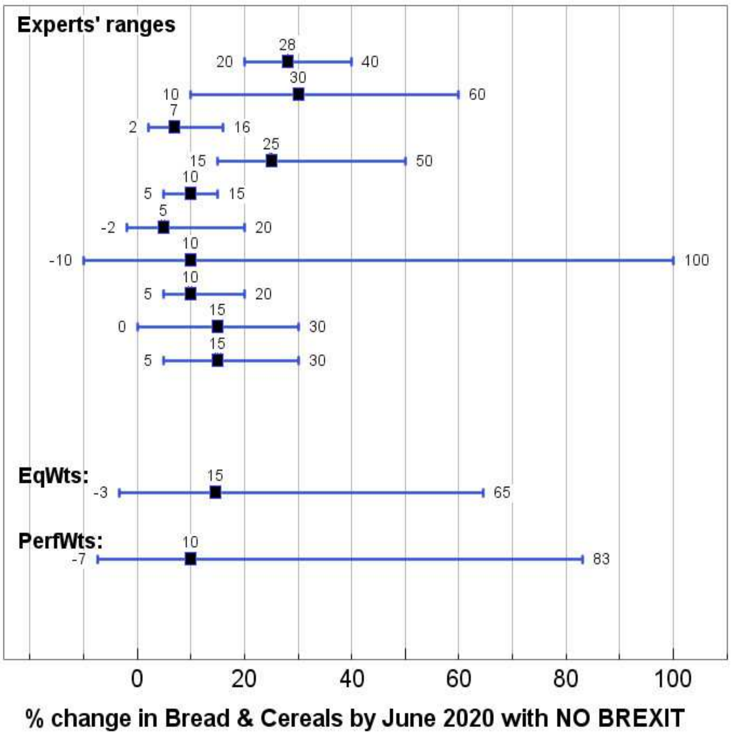

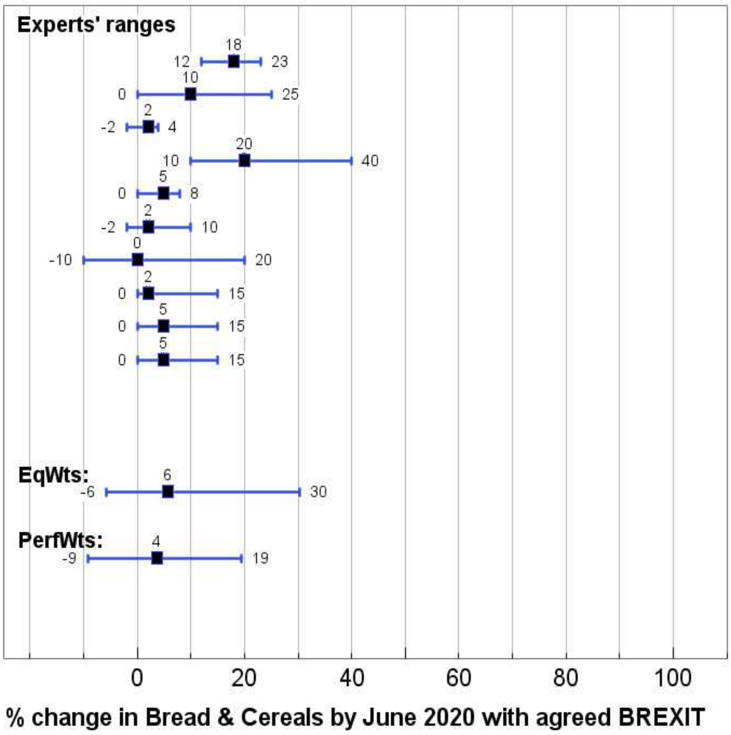

Barons MJ, Aspinall W. *BMJ Open* 2020; 10:e032376. doi: 10.1136/bmjopen-2019-032376

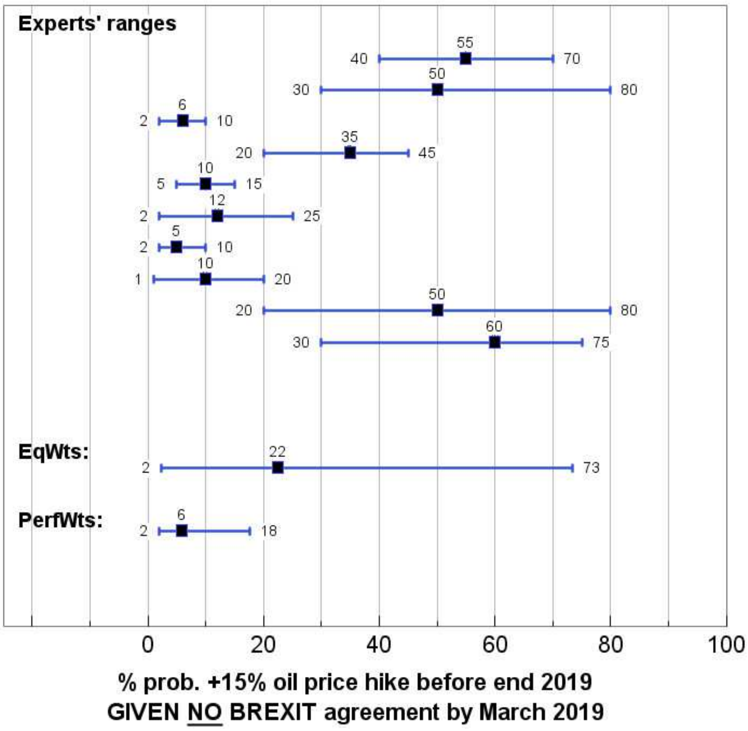

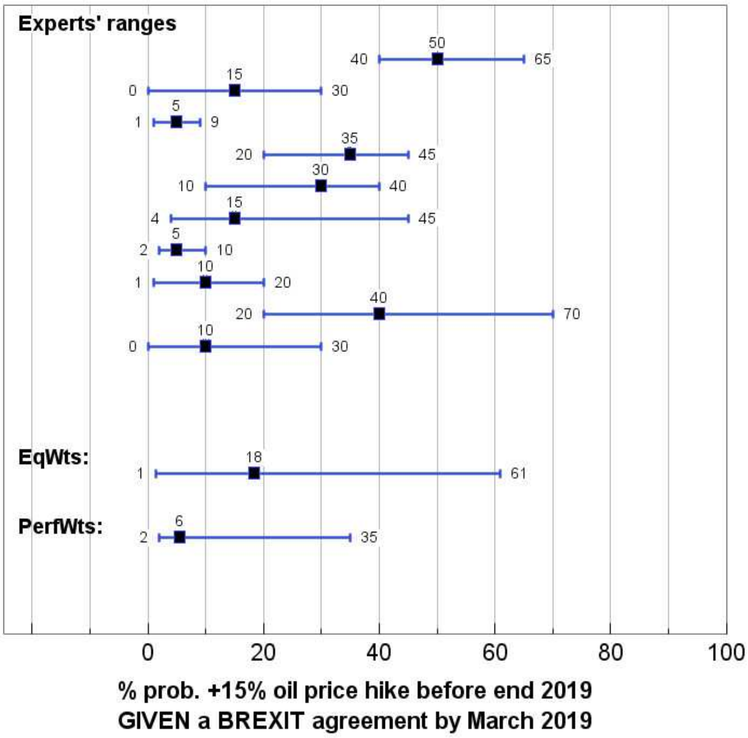

Barons MJ, Aspinall W. *BMJ Open* 2020; 10:e032376. doi: 10.1136/bmjopen-2019-032376

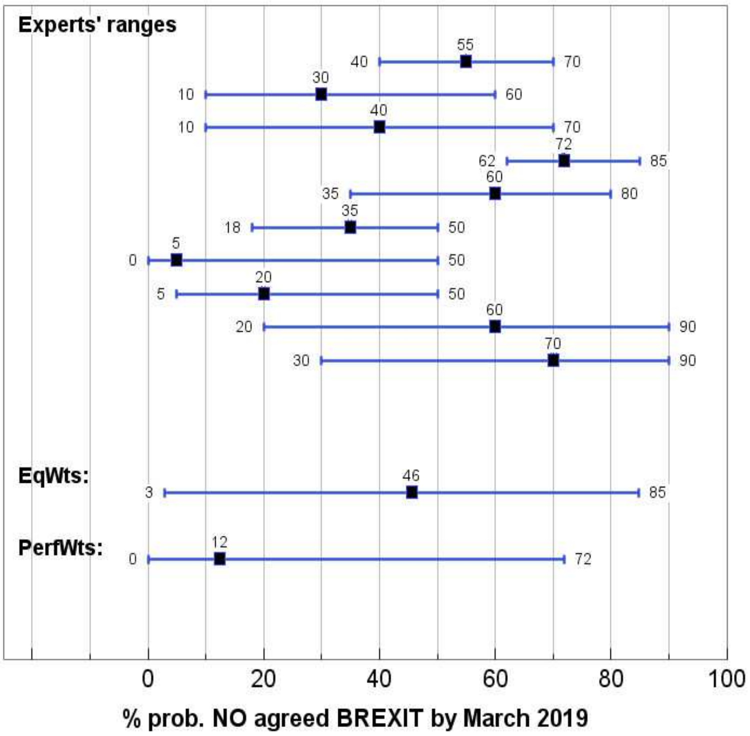

Barons MJ, Aspinall W. *BMJ Open* 2020; 10:e032376. doi: 10.1136/bmjopen-2019-032376

Discussion of results

The elicitation was judged to be highly successful by the experienced facilitator (WPA).

As noted above, three of the ten scored experts achieved significant performance weights. The performance weighted (PW) aggregation solutions, as influenced by these three experts and their weights, reduced group-wide uncertainty spreads in most cases.

The following notes record immediate panel member verbatim comments on preliminary results from the elicitation, which were presented to the group on the final morning of the workshop; n.b. target item solutions, as presented in the previous section, were subsequently finalised after additional responses were received from some participants.

Comments recorded here are not attributed to individual participants.

Target item comments

1. *Food prices with and without a BREXT trade deal in place*
    a. *Bread and cereals*

- Agreed that there was expected uncertainty relating to the ‘no BREXIT deal’ graph so were not surprised by the bounds widening.
- One expert pointed out that this is an individual food category, if the question considered the entire CPI basket as a single variable then their own bounds would be smaller, but for this one category their wide bounds were truly meant to reflect their uncertainty.
- Bread and cereals category is a mix of domestic flours for biscuits and animal feed, imported cereals from Canada and the US. This is a compounded first order effect of what happens to the supply.
- Input of information that the UK imports Canadian flour for making bread, but uses domestic flour for items such as biscuits. Some experts said that they assumed most of our cereals came from USA which would have little impact from BREXIT deals.
- Noted that cereals are highly weather dependent, so regardless of BREXIT we have to consider crop yield dependent on environmental factors.
- How much are farmers going to plant? What’s the weather like? What’s the yield? What happens elsewhere in the world? If we don’t have the protection of the EU, how do we meet the supply? What if we have to import ‘poor quality’ food, whether that means actually poor nutritional quality or ‘poor’ due to not meeting the technical specifications?
- Another factor that would affect the price is a heightened risk of job losses, which would result in less purchasing power in the population as a whole.
- In several of the instances we’ve looked at, then the substitution of goods also impacts the answer to this question. Potatoes and cereal don’t necessarily substitute for each other, although they’re both starches.
- With respect to potatoes, consider the fact that only half of the US crops are irrigated. If you have drought and flooding, then potato prices might shoot up in October [2019].
- One expert said they always tried to measure substitutes, for example if potatoes were blighted then they would expect more people to move to grains if their household was closer to food insecurity.

b. MEAT

- 70% of the cost of pork is attributed to wheat for feed; therefore this is heavily linked to the previous questions. However, most experts had not realised this so did not factor it into their judgements.
- One expert mentioned that the performance weights median seems too low because they reckon that we would see more climate change effects, even in the two-year short-term.
- Experts believed that with 'no BREXIT' deal then there would be a decrease in price for New Zealand lamb and South American meat due to the sudden influx in the market.
- Cattle are either dairy or meat assigned therefore no overlap in effects of meat and milk. Slaughtered dairy cows' meat is used in processed food such as pies and pasties, and not for joints of beef at the supermarket, etc. The calves born to initiate lactation are sold for meat, although some females may be retained to become milkers in future.

c. FISH

- Fish prices could go down quite a lot.
- UK fish comes predominantly from Norway; UK sells the fish it catches.
- Seas sovereignty is currently 40%. There was an expectation amongst the industry that this would increase to 80% after BREXIT, but that currently [July 2018] seems unlikely.
- We probably cannot police the seas as we have no military boats.

d. MILK, CHEESE, EGGS

- Lead time for hard cheese is anything up to one year, so would be a lag time effecting cheddar cheese.
- But soft cheeses, such as mozzarella, have a very short lead time and these are the cheeses used on pizzas and in ready meals; there are different lead times for each of the different cheese types.
- If milk subsidy disappeared, more farmers might turn to producing meat. How fast would the changes be with each of these?

e. OILS & FATS

- One expert was not happy that the aggregated answers for oils & fats are roughly the same as for the meats item – they believe that the median and uncertainty in meat should be much higher.
- Food oil is completely imported.
- Interconnected to petroleum oil due to biofuel industry.
- Price of crude oil directly affects the price of oil and fats.

f. FRUIT

- The UK imports 82% of fruit, a lot of these are non-EU countries; however, they have free EU trade agreements

g. VEGETABLES

- There seems to be more uncertainty associated with vegetables than with previous food categories in **either** situation (BREXIT deal vs. no-BREXIT deal) because of the inherent impacts of weather/seasonality.
- Most experts agree that uncertainty around vegetables is not as great as uncertainty around fruit because of the import numbers.
- Vegetable substitutions are vast. One expert hypothesised that the British public would be happy to drop certain supermarket fruits from diets, if these were too expensive. This is true for fish too, but the general consensus is that the public is much fussier about changing fish type than fruit type.
- Some crops (potatoes) have bulking phase in their growth cycle, so weather and the timing of rainfall, sun, etc. have a significant effect on yield.

Barons MJ, Aspinall W. *BMJ Open* 2020; 10:e032376. doi: 10.1136/bmjopen-2019-032376

h. SUGAR+JAM

- Sugar beet is grown in the UK in very focussed areas so flooding or rainfall in these areas has serious consequences (however, not sure this will change by 2020).
- Trade policy important for sugarcane and jam. One major producer of cane sugar is an advocate of BREXIT because of profit opportunity by reduction of cane tariff; the other major is a sugar beet producer.

i. COFFEE, TEA, COCOA

- In CPI terms, this category means instant and ground coffee or tea bags and loose tea sold in supermarkets, not drinks sold in shops.
- Huge market, all imported, subject to exchange rate and tariffs.
- Tea / coffee pricing can be controlled to some extent by different blending, e.g. by diluting Arabica.

j. SOFT DRINKS

- Relatively speaking, there seems to be much less future pricing uncertainty than for other foodstuffs.
- Soft drinks can easily recalibrate recipes and ingredients to accommodate changing prices of raw materials, e.g. sugar.
- One expert pointed out that they cannot recall Coca-cola or Pepsi changing their prices in our lifetime, which makes it seem like they are unlikely to change in the future

Barons MJ, Aspinall W. *BMJ Open* 2020; 10:e032376. doi: 10.1136/bmjopen-2019-032376

Appendix S1.1 Target items questionnaire

For each question, your three values should be unique (i.e. no ties), and should be in ascending order: i.e. Low < Median < High.

First, three questions about the probabilities of two potential major external factors that could influence Food prices.

TQ1a. This question concerns BREXIT

What is the probability there will be NO agreed EU-UK BREXIT deal by March 2019? Please give your range of judgments for the low-end, median and high-end probabilities of this happening, expressed as percentages in range 0 – 100%.
________ **Low (5th)** ________ **Median (50th)** ________ **High (95th)**

Future Food Prices changes: outlook timeframe is June 2020.

TQ2a. This pair of questions concern *Bread & cereals* prices and BREXIT

GIVEN current quarterly Bread & Cereals spend per household is £141.55 AND GIVEN there is an agreed EU-UK BREXIT deal by March 2019, please give your judgments of the low-end, median and high-end percentage changes in Bread & Cereals spend per household by June 2020. (A decrease should be expressed as a negative percentage change).
________ **Low (5th)** ________ **Median (50th)** ________ **High (95th)**

TQ2b.

GIVEN current quarterly Bread & Cereals spend per household is £141.55 AND GIVEN there is NO BREXIT deal by March 2019, please give your judgments of the low-end, median and high-end percentage changes in Bread & Cereals spend per household by June 2020. (A decrease should be expressed as a negative percentage change).
________ **Low (5th)** ________ **Median (50th)** ________ **High (95th)**

Barons MJ, Aspinall W. *BMJ Open* 2020; 10:e032376. doi: 10.1136/bmjopen-2019-032376

TQ3a. This pair of questions concern Meat prices and BREXIT

GIVEN current quarterly Meat spend per household is £188.12 AND GIVEN there is an agreed EU-UK BREXIT deal by March 2019, please give your judgments of the low-end, median and high-end percentage changes in Meat spend per household by June 2020. (A decrease should be expressed as a negative percentage change).
__________ **Low (5th)** __________ **Median (50th)** __________ **High (95th)**

TQ3b.

GIVEN current quarterly Meat spend per household is £188.12 AND GIVEN there is NO BREXIT deal by March 2019, please give your judgments of the low-end, median and high-end percentage changes in Meat spend per household by June 2020. (A decrease should be expressed as a negative percentage change).
__________ **Low (5th)** __________ **Median (50th)** __________ **High (95th)**

TQ4a. This pair of questions concern *Fish* prices and BREXIT

GIVEN current quarterly Fish spend per household is £40.47 AND GIVEN there is an agreed EU-UK BREXIT deal by March 2019, please give your judgments of the low-end, median and high-end percentage changes in Fish spend per household by June 2020. (A decrease should be expressed as a negative percentage change).
__________ **Low (5th)** __________ **Median (50th)** __________ **High (95th)**

TQ4b.

GIVEN current quarterly Fish spend per household is £40.47 AND GIVEN there is NO BREXIT deal by March 2019, please give your judgments of the low-end, median and high-end percentage changes in Meat spend per household by June 2020. (A decrease should be expressed as a negative percentage change).
__________ **Low (5th)** __________ **Median (50th)** __________ **High (95th)**

Barons MJ, Aspinall W. *BMJ Open* 2020; 10:e032376. doi: 10.1136/bmjopen-2019-032376

TQ5a. This pair of questions concern *Milk, cheese & eggs* prices and BREXIT

GIVEN current quarterly Milk, cheese & eggs spend per household is £110.37 AND GIVEN there is an agreed EU-UK BREXIT deal by March 2019, please give your judgments of the low-end, median and high-end percentage changes in Milk, cheese & eggs spend per household by June 2020.
(A decrease should be expressed as a negative percentage change).

___________	___________	___________
**Low (5th)**	**Median (50th)**	**High (95th)**

TQ5b.

GIVEN current quarterly Milk, cheese & eggs spend per household is £110.37 AND GIVEN there is NO BREXIT deal by March 2019, please give your judgments of the low-end, median and high-end percentage changes in Milk, cheese & eggs spend per household by June 2020.
(A decrease should be expressed as a negative percentage change).

___________	___________	___________
**Low (5th)**	**Median (50th)**	**High (95th)**

TQ6a. This pair of questions concern *Oil & fats* prices and BREXIT

GIVEN current quarterly Oil & fats spend per household is £19.65 AND GIVEN there is an agreed EU-UK BREXIT deal by March 2019, please give your judgments of the low-end, median and high-end percentage changes in Oil & fats spend per household by June 2020.
(A decrease should be expressed as a negative percentage change).

___________	___________	___________
**Low (5th)**	**Median (50th)**	**High (95th)**

TQ6b.

GIVEN current quarterly Oil & fats spend per household is £19.65 AND GIVEN there is NO BREXIT deal by March 2019, please give your judgments of the low-end, median and high-end percentage changes in Oil & fats spend per household by June 2020.
(A decrease should be expressed as a negative percentage change).

___________	___________	___________
**Low (5th)**	**Median (50th)**	**High (95th)**

Barons MJ, Aspinall W. *BMJ Open* 2020; 10:e032376. doi: 10.1136/bmjopen-2019-032376

TQ7a. This pair of questions concern *Fruit* prices and BREXIT

GIVEN current quarterly Fruit spend per household is £94.94 AND GIVEN there is an agreed EU-UK BREXIT deal by March 2019, please give your judgments of the low-end, median and high-end percentage changes in Fruit spend per household by June 2020.<br>(A decrease should be expressed as a negative percentage change).
__________ **Low (5th)** __________ **Median (50th)** __________ **High (95th)**

TQ7b.

GIVEN current quarterly Fruit spend per household is £94.94 AND GIVEN there is NO BREXIT deal by March 2019, please give your judgments of the low-end, median and high-end percentage changes in Fruit spend per household by June 2020.<br>(A decrease should be expressed as a negative percentage change).
__________ **Low (5th)** __________ **Median (50th)** __________ **High (95th)**

TQ8a. This pair of questions concern *Vegetables* prices and BREXIT

GIVEN current quarterly Vegetables spend per household is £123.55 AND GIVEN there is an agreed EU-UK BREXIT deal by March 2019, please give your judgments of the low-end, median and high-end percentage changes in Vegetables spend per household by June 2020.<br>(A decrease should be expressed as a negative percentage change).
__________ **Low (5th)** __________ **Median (50th)** __________ **High (95th)**

TQ8b.

GIVEN current quarterly Vegetables spend per household is £123.5 AND GIVEN there is NO BREXIT deal by March 2019, please give your judgments of the low-end, median and high-end percentage changes in Vegetables spend per household by June 2020.<br>(A decrease should be expressed as a negative percentage change).
__________ **Low (5th)** __________ **Median (50th)** __________ **High (95th)**

TQ9a. This pair of questions concern *Sugar, jam etc* prices and BREXIT

Barons MJ, Aspinall W. *BMJ Open* 2020; 10:e032376. doi: 10.1136/bmjopen-2019-032376

GIVEN current quarterly Sugar, jam, etc. spend per household is £122.78 AND GIVEN there is an agreed EU-UK BREXIT deal by March 2019, please give your judgments of the low-end, median and high-end percentage changes in Sugar, jam, etc. spend per household by June 2020. (A decrease should be expressed as a negative percentage change).
________ **Low (5th)** ________ **Median (50th)** ________ **High (95th)**

TQ9b.

GIVEN current quarterly Sugar, jam, etc. spend per household is £122.78 AND GIVEN there is NO BREXIT deal by March 2019, please give your judgments of the low-end, median and high-end percentage changes in Sugar, jam, etc. spend per household by June 2020. (A decrease should be expressed as a negative percentage change).
________ **Low (5th)** ________ **Median (50th)** ________ **High (95th)**

TQ10a. This pair of questions concern *Coffee, tea & cocoa* prices and BREXIT

GIVEN current quarterly Coffee, tea & cocoa spend per household is £31.88 AND GIVEN there is an agreed EU-UK BREXIT deal by March 2019, please give your judgments of the low-end, median and high-end percentage changes in Coffee, tea & cocoa spend per household by June 2020. (A decrease should be expressed as a negative percentage change).
________ **Low (5th)** ________ **Median (50th)** ________ **High (95th)**

TQ11b.

GIVEN current quarterly Coffee, tea & cocoa spend per household is £31.88 AND GIVEN there is NO BREXIT deal by March 2019, please give your judgments of the low-end, median and high-end percentage changes in Coffee, tea & cocoa spend per household by June 2020. (A decrease should be expressed as a negative percentage change).
________ **Low (5th)** ________ **Median (50th)** ________ **High (95th)**

TQ12a. This pair of questions concern *Soft drinks etc.* prices and BREXIT

Barons MJ, Aspinall W. *BMJ Open* 2020; 10:e032376. doi: 10.1136/bmjopen-2019-032376

GIVEN current quarterly <u>Soft drinks etc.</u> spend per household is £82.75 AND GIVEN there is an agreed EU-UK BREXIT deal by March 2019, please give your judgments of the low-end, median and high-end <u>percentage changes</u> in Soft drinks etc. spend per household by June 2020.<br>(A decrease should be expressed as a negative percentage change).
___________ **Low (5th)** ___________ **Median (50th)** ___________ **High (95th)**

TQ12b.

GIVEN current quarterly <u>Soft drinks etc.</u> spend per household is £82.75 AND GIVEN there is NO BREXIT deal by March 2019, please give your judgments of the low-end, median and high-end <u>percentage changes</u> in <u>Soft drinks etc.</u> spend per household by June 2020.<br>(A decrease should be expressed as a negative percentage change).
___________ **Low (5th)** ___________ **Median (50th)** ___________ **High (95th)**

Appendix S1.2 Workshop Invitation

On 21 June 2018 at 20:20, Barons, Martine <Martine.Barons@warwick.ac.uk> wrote:

I recently invited you to take part in a research exercise on food prices as part of developing decision support for policymakers concerned with food insecurity, reliance on food banks etc.

It would be very helpful if you could let me know in the next day or two whether you are able to participate.

There is a minimum number of participants required for the research exercise to run, and if the exercise needs to be cancelled, I would like to give reasonable notice to those who have already offered their time and to Prof Willy Aspinall, who is in high demand and may be able to accommodate other bookings if we cancel.

Best wishes, Martine

Dr Martine J. Barons CMath MIMA AMInstP
Director of the Applied Statistics and Risk Unit
Department of Statistics
University of Warwick
C0.10 | Zeeman Building | University of Warwick | CV4 7AL
martine.barons@warwick.ac.uk e-mail
http://www2.warwick.ac.uk/fac/sci/statistics/staff/academic-research/barons Web page

---

From: Barons, Martine
Sent: 21 May 2018 16:39

Subject: Food Price scenario workshop invitation

I would like to invite you to participate in a structured expert elicitation exercise to be facilitated by Prof. Willy Aspinall at the University of Warwick, 4th – 6th July 2018. Invitees will be asked to suggest what, in their best judgement would be the effects of various scenarios, such as oil price changes, food scares, natural disaster, trade deals, on the UK price of foodstuffs as categorised in the Consumer Prices Index.

We will acknowledge your contribution unless you tell us you would prefer not to be credited and all discussions will be held under Chatham House rules, i.e. anyone who comes to the meeting is free to use information from the discussion, but is not allowed to reveal who made any comment.

We are looking for up to 20 people to give opinions on one or more foodstuffs. You have been invited since we believe you are expert in this area. If you are unable to attend yourself, please nominate a suitably qualified colleague.

Whilst we are unable to pay for your time, all UK travelling expenses will be covered, all meals provided and overnight accommodation provided on campus if required.

This structured elicitation forms part of ongoing research by myself and Professor Jim Smith on decision support for policies designed to ameliorate food poverty. We are collaborating with local authorities and so our interest is the effects on UK food prices.

Prof Willy Aspinall is Cabot Professor in Natural Hazards & Risk Science, University of Bristol.

The exercise will proceed as follows:
4th July: arrive in time for lunch. Afternoon briefing on protocol and discussion of scenarios and target questions. Evening, social dinner and overnight accommodation on campus if required.
5th July: Main structured elicitation exercise, evening meal, and accommodation on campus if required.
6th July: Feedback and final elicitation items if required. Leave after lunch.

I do hope you will be able to join us for this event.

Please let me know either way at your earliest convenience. Here is the form to capture dietary and accommodation requirements http://www2.warwick.ac.uk/fac/sci/statistics/asru/elicitation.

Dr Martine J. Barons CMath MIMA AMInstP
Director of the Applied Statistics and Risk Unit
go.warwick.ac.uk/ASRU
Department of Statistics |University of Warwick
C0.10 | Zeeman Building |University of Warwick | CV4 7AL
martine.barons@warwick.ac.uk e-mail
go.warwick.ac.uk/MJBarons Web page

WARWICK
THE UNIVERSITY OF WARWICK

28

Appendix S1.3 Briefing note sent to participants

Expert Elicitation and Judgment Pooling using the Cooke Classical Model and EXCALIBUR

Short briefing note for participants in an Expert Elicitation

Prepared by Willy Aspinall

July 2018

Important statement

In this note, a formal mathematical process is described for deriving objective differentiating weights for a group of experts in order to pool their opinions on questions of interest to the project in an optimal way. This calibration is based on empirical testing of individuals' abilities to provide informative uncertainty distributions capturing known realization values for certain related subject-matter quantities. The resulting calibration scores are NOT a measure of knowledge, scholarship or intelligence – but simply furnish a rational basis for setting out an understanding - for decision-support - of how uncertain parameters may be represented when alternative methods or solutions are not available.

1 Essentials

Expert opinion is almost invariably sought when technical uncertainty impacts on an important decision process. Because such uncertainty is ubiquitous in scientific knowledge - if it were not, any decision related to the issue at hand would be obvious – there is the inescapable corollary that the experts themselves cannot be absolutely certain, and thus it is extremely unlikely they will ever be in total agreement with one another. This is especially true where such uncertainty is substantial, or where the consequences of the decision are particularly serious or onerous.

In circumstances where scientific uncertainty impinges on the determination of an issue, soliciting expert advice is not new. Generally, however, this has been pursued on an informal basis, and such an unstructured approach is rarely, if ever, found entirely satisfying to all parties. Neither is it likely to be immune to legitimate criticism, from one side or another. To counteract these shortcomings, a *structured* expert judgment elicitation refers to the deliberate effort to subject the whole process to transparent methodological rules, with the goal of treating expert judgments as scientific data in a formal decision process.

Various methods for assessing and combining expert uncertainty are available in the literature. Some advocate a group decision-conferencing consensus approach for eliciting opinions, for instance, but other approaches exist for carrying out this process. Notable amongst these is the expert weighting procedure known as the Classical Model, formulated by Cooke (1991), which can be implemented with the computer package EXCALIBUR to analyse opinions obtained through a structured elicitation procedure. The unique feature of this approach is that it is the only one in which distinct weights are given to individual experts, based statistically on their ability to judge uncertainties as determined empirically by performance metrics derived from control questions; the theoretical basis and

principles of the Classical Model are expounded in Cooke (1991), and a recent summary of case histories using the procedure is given in Cooke and Goossens (2008).

1.1 The EXCALIBUR Procedure

The main steps in the procedure for applying the EXCALIBUR approach in practice can be summarised as follows:

- A group of experts are selected.
- Experts are elicited individually regarding their uncertainty over the results of possible measurements or observations within their domain of expertise.
- Experts also assess variables within their field, the true values of which are known or become known post hoc.
- Experts are treated as statistical hypotheses and are scored with regard to statistical likelihood (often called ‘calibration’) and informativeness.
- Scores are combined to form weights. These weights are constructed to be ‘strictly proper scoring rules’ in an appropriate asymptotic sense: experts receive their maximal expected long-run weight by, and only by, stating their true degrees of belief. With these weights, statistical accuracy strongly dominates informativeness – one cannot compensate poor statistical performance by very high information.
- Likelihood and informativeness scores are used to derive performance- based weighted combinations of the experts’ uncertainty distributions.

The key feature of this method is the performance-based combination of expert uncertainty distributions. When it comes to attempting to resolve differences in expert judgments, people who seek to find a harmony of views by conciliation can be disconcerted by this approach, but extensive experience overwhelmingly confirms that experts grow to favour it because its performance measure are entirely objective and amenable to diagnostic examination.

1.2 Combining expert assessments to form a Decision Maker

A combination of expert assessments is often referred to as a “decision maker” (DM), in the sense of linear pooling. The steps in the process by which one can arrive at a decision maker outcome are summarised and illustrated schematically in Fig. 1. On the left-hand side of this diagram, hypothetical examples of the responses of three different experts to three seed questions are depicted, showing how their calibration can vary, in relation to the true realization value for the seed item, and how their information can also vary, generally from expert-to-expert, rather than within experts. Note that each expert is required to provide a fixed number of quantiles (usually three) to express his or her degree of belief in their judgment of the seed item value and the credible interval within which it should fall in their opinion.

With a set of several seed items (usually about ten in number), a group of experts can be ranked according to their individual calibration and information scores, and then according to the weights overall, as determined by the product of calibration and information scores. With these latter weights

Barons MJ, Aspinall W. BMJ Open 2020; 10:e032376. doi: 10.1136/bmjopen-2019-032376

to hand, it is then possible to elicit from the same group of experts their quantile-based distributions for items of interest (i.e. for questions for which an expert consensus is sought), and these individual response distributions can be linearly pooled, applying the individual weights. It should be noted that a weighted combination distribution, obtained in this way, is seldom if ever identical to the distribution of any one contributing expert, but does represent a rational consensus of the information provided by members of the group as a whole, differentiated by their performance on the seed items.

The Classical Model is essentially a formal method for deriving the requisite weights for a linear pool in which, as just noted, these weights are expressed as the product of an individual's calibration and information scores. "Good expertise" corresponds to good calibration (high statistical likelihood the expert's distributions reflect true values) and superior information. Strong weights reward good expertise, and pass these virtues on to the decision maker.

The reward aspect of weights is very important. An expert's influence on the decision maker should not appear haphazard, and he/she should be discouraged from attempting to game the system by tilting his/her assessments to achieve a desired outcome. Thus, it is necessary to impose a strictly proper scoring rule constraint on the weighing scheme. Roughly speaking, this means that an expert achieves his maximal expected weight by, and only by, stating assessments in conformity with his/her true beliefs.

2 Remarks

In the Classical Model, calibration and information are combined to yield an overall or combined score with the following attributes:

1. Individual expert assessments, realizations and scores can be recorded. This enables any reviewer to check the application of the method, in compliance with the principle of **accountability / scrutability.**
2. Performance is measured and hopefully validated, in compliance with the principle of **empirical control.** An expert's weight is determined by performance on seed items.
3. The score is a long run proper scoring rule for average probabilities, in compliance with the principle of **neutrality.**
4. Experts are treated equally, prior to the performance measurement, in compliance with the principle of **fairness.**

Whilst expert names and qualifications should be part of the documentation of every expert judgment study, they are not usually associated directly with identifiable individual assessments in the open literature. The experts' reasoning is always recorded and that is sometimes published as expert rationales.

There is no mathematical theorem which states that either item weights or global weights will out-perform equal weights or out-perform the best expert. Indeed, it is not difficult to construct artificial examples where this is not the case. Selecting which of these weighting schemes to use is a matter of experience. In practice, global weights are used unless item weights perform markedly better.

Of course, there may be other ways of defining expert weights that perform better, and indeed there might be better performance measures. But, good performance on a one-off basis for a single individual data set is not convincing. What is convincing is good performance on a large diverse data

set, such as the TU Delft expert judgment database (Cooke and Goossens, 2008; Colson and Cooke, 2017). In practice a method should be easy to apply, easy to explain, should do better than equal weighting and should never do something ridiculous.

More than one hundred different expert elicitations involving seed variables have been performed to date. These are all studies performed under contract for a problem owner, and reviewed and accepted by the contracting party. In most cases the results have been published. Given the body of experience with structured expert judgment that has now accumulated, the performance-based Classical Model approach is well established: as mentioned earlier, simply using equal weights for scientific uncertainty quantification no longer seems to be a convincing alternative.

Colson and Cooke (2017) have updated the TU Delft structured expert judgment database with data from 33 recent professionally-contracted Classical Model studies, and have evaluated the Classical Model's performance relative to other expert aggregation models. Performance weighting outperformed equal weighting in all but 1 of the 33 studies in-sample. For out-of-sample testing of the sets of calibration variables, overall the information of performance-based combinations is typically double that of equal weights combinations. Colson and Cooke (2017) propose an Out-of-Sample Validity Index based on averaging the product of statistical accuracy and information over all training sets sized at 80% of the calibration set. Performance weighting outperforms equal weighting on this Out-of-Sample Validity Index in 26 of the 33 studies; the probability of 26 or more successes on 33 trials -- if there were no difference between performance weighting and equal weighting -- is 0.001.

This experience shows that in the great majority of cases, the Classical Model performance-based combination of expert judgments gives more informative and statistically more accurate results than either the best individual expert or the 'equal weights' combination of all expert distributions. Upon reflection, it is evident that equal weighting has a very serious drawback. As the number of experts increases, the equal weight combination typically becomes increasingly diffuse, until it represents no one's belief and is useless for decision support. This is frequently seen as the number of experts exceeds, say, eight. The viability of equal weighting is maintained only by severely restricting the number of experts who will be treated equally, leaving others outside the process. It appeals to a sort of one-man-one-vote consensus ideal. Progress in science, however, is driven by rational consensus.

Ultimately, consensus is an equilibration of power; in science, it is not the power of the ballot but the power of arguments that counts (Kurowicka and Cooke, 2006), and this can be made manifest through the Classical Model / EXCALIBUR structured elicitation procedure.

Bibliography

Aspinall, W.P., 2006. Structured elicitation of expert judgement for probabilistic hazard and risk assessment in volcanic eruptions. In: Statistics in Volcanology (eds. H.M. Mader, S.G. Coles, C.B. Connor and L.J. Connor) - Special Publications of IAVCEI No. 1; London, The Geological Society for IAVCEI: 15-30.

Brown, A.J. and Aspinall, W.P., 2004. Use of expert elicitation to quantify the internal erosion processes in dams. Proceedings of the British Dam Society Conference, Thomas Telford, pp 282-297

Colson, A. R. and R. M. Cooke (2017). Cross validation for the classical model of structured expert judgment. Reliability Engineering and System Safety 163: 109-120.

Cooke, R. M., 1991. Experts in Uncertainty - Opinion and Subjective Probability in Science. Environmental Ethics and Science Policy Series. Oxford University Press, ISBN 0195064658.

Cooke, R.M., 2008. Guest Editorial, Special Issue on Expert Judgment. Reliability Engineering & System Safety. In press, corrected proof. doi:10.1016/j.ress.2007.03.001

Cooke, R.M. and Goossens, L.L.H.J., 2008. TU Delft expert judgment data base. Reliability Engineering & System Safety. In press, corrected proof. doi:10.1016/j.ress.2007.03.005.

Goossens L., Cooke R. and Kraan B. (1998) Evaluation of weighting schemes for expert judgment studies. PSAM4 Proceedings, eds. A. Mosleh and R.A. Bari. Vol. 4. Springer, 1937-1942.

Kurowicka, D. and Cooke, R., 2006. *Uncertainty Analysis with High Dimensional Dependence Modelling*. Wiley, Series in Probability and Statistics, Chichester: 284pp.

Fig. 1 Schematic chart showing how experts responses are calibrated against (multiple) seed

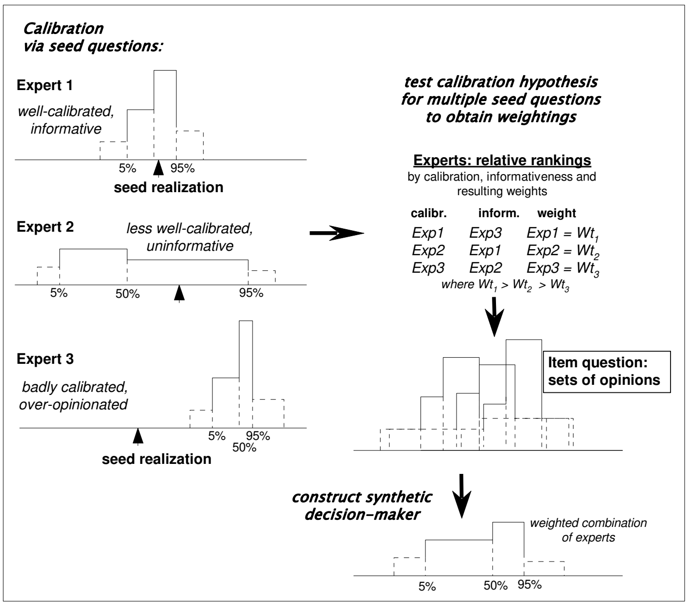

questions at given quantiles to produce performance-based weights; these are then used to pool the experts' opinions on corresponding quantiles for target questions which the problem owner wants quantified.
