# Supplementary material for: Anticipated impacts of Brexit scenarios on UK food prices and implications for policies on poverty and health: a structured expert judgement approach
Source: BMJ Open. 2020 Mar 3;10(3):e032376. doi: 10.1136/bmjopen-2019-032376 (PMC7059525; doi:10.1136/bmjopen-2019-032376)
Supplement: Supplementary data [file bmjopen-2019-032376supp002.pdf]

Supplementary Information – File S2

Barons, M. and Aspinall, W. **Anticipated impacts of Brexit scenarios on UK food prices and implications for policies on poverty and health: a structured expert judgement approach**

UNINET

UNINET enables efficient calculation of Bayes rule equations and rapid parameter conditionalization for making probabilistic inferences from observations. Conditionalizing a large network on multiple disparate observations and propagating this information through a multi-nodal model is analytically challenging; heuristics and simple statistical models can often lead us astray. BNs not only solve particular problems of information synthesis, they also provide a basis for reasoning probabilistically and testing model assumptions.

The main features of UNINET (distinguishing it from other packages) are that it supports non-parametric continuous or discrete BBNs, supplemented with functional nodes. Probabilistic nodes may be assigned arbitrary continuous or discrete distributions. UNINET has the valuable attribute of accepting Decision Maker results directly from EXCALIBUR output. Thus, Classical Model DM quantile histograms can be compiled into minimum information distribution form, without pre-processing, and used for propagating uncertainties into the desired analysis calculations.

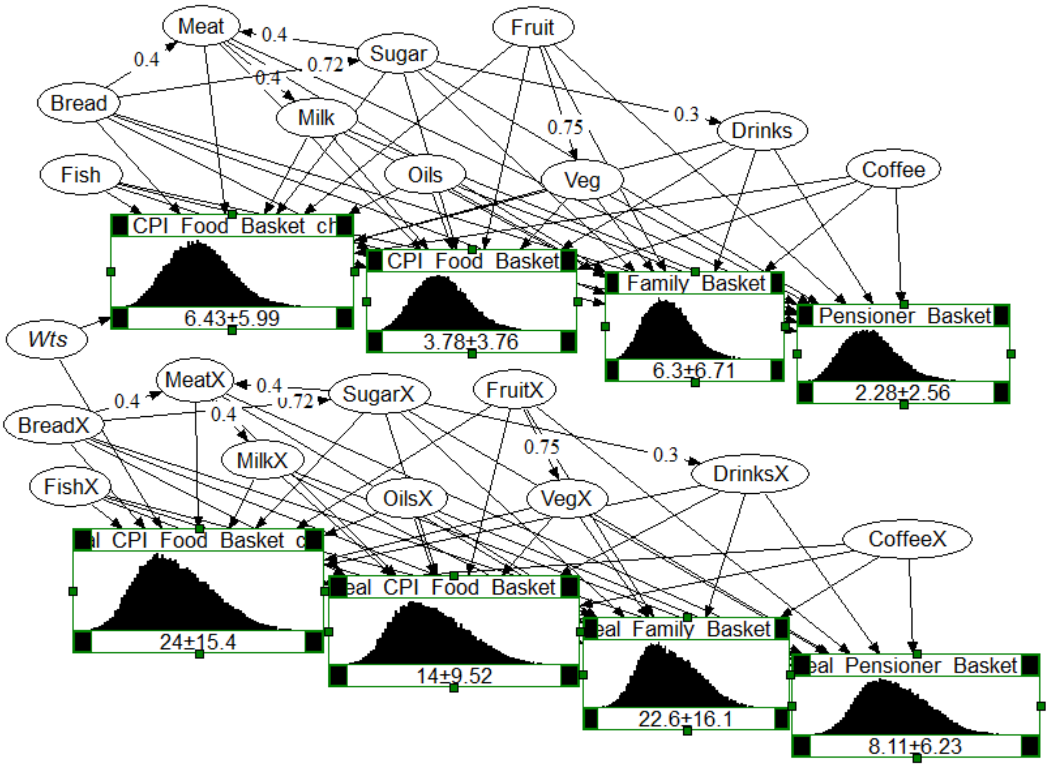

Figure A1 Bayes Net calculated output node distributions for percentage change in CPI Food Basket cost for Brexit Deal and No deal scenarios (LH pair of histograms) and for cost change in CPI Food Basket and two household cases in £, for the same Brexit scenarios. Each histogram panel reports the mean ± 1 s.d. change for that target item; other distribution statistics are output numerically by UNINET. Note hints of long upper tail skewness in every case (see text for discussion).

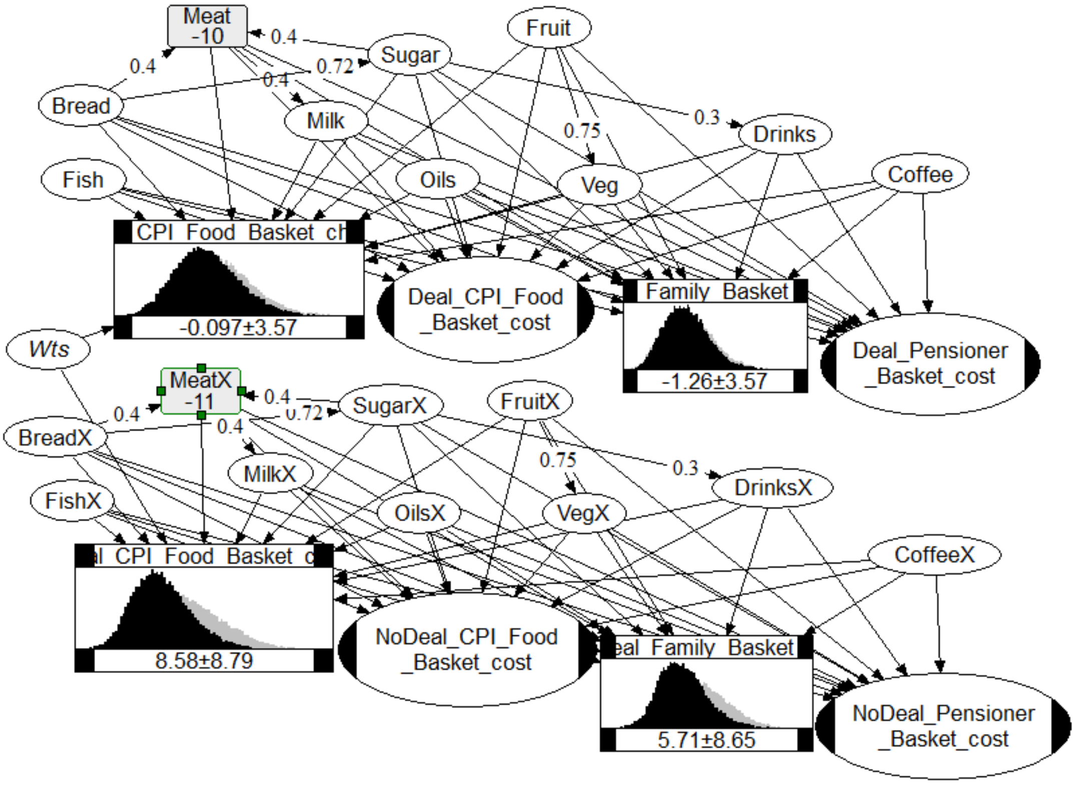

Figure A2 Bayes Net for Food baskets prices conditionalized on projected 5th percentile Meat (Deal: -10% change) and MeatX (No deal: -11% change) costs. The resulting updated target node distributions, due to conditionalizing Meat costs, are shown in black, the original model unconditionalized distributions are shown in grey.

A valuable attribute of UNINET, of particular benefit in the present study, is its capability to allow specific value conditioning on any number of probabilistic nodes. Analytic conditioning comprises either: (a) conditioning on a single value, which applies only for probabilistic nodes; or (b) sample-based conditioning, by which means the user can conditionalize on specific value intervals (for probabilistic and functional nodes). Thus, UNINET can model an empirical multivariate distribution by building a joint density function, which can be analytically conditioned. This capability is used here for conducting a pair of example sensitivity tests, based on selected Meat cost changes.

The UNINET BN is re-run with two food category nodes (deal Meat and no-deal MeatX) conditionalized to their 5th percentile levels from the elicitation distributions (-10% and -11%, respectively). In this example, we examine the effects of fixing these two nodes on the deal and no-deal outcome CPI basket percentage change and on the family-of-four basket cost, respectively. The resulting updated target node distributions, due to conditionalizing Meat costs, are shown on Figure A2.

Next, we run the BN with Meat (Deal) and MeatX (No deal) conditionalized to their elicited 95th percentile changes, i.e. +29% and +80%, which represent price increases well above the

elicited medians, respectively (Figure A3). Under the Brexit no-deal scenario: the CPI Basket mean change would be +44.0% (cf +24.0%) %)

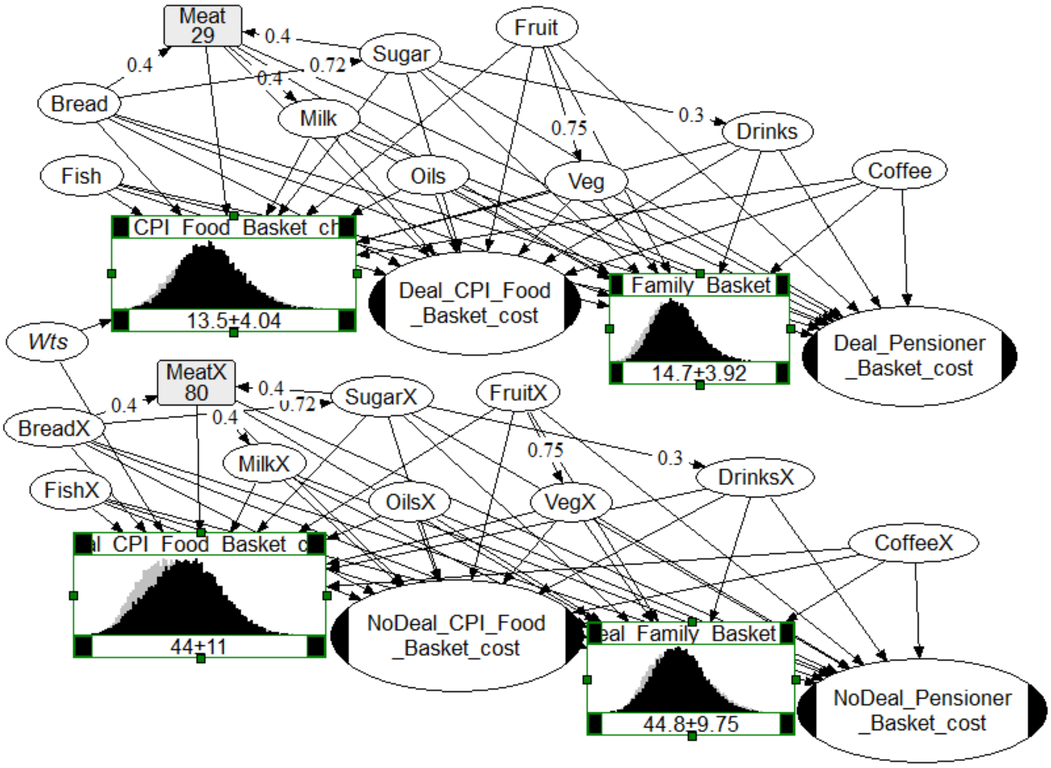

Figure A3 Bayes Net for Food baskets prices conditionalized on projected 95th percentile costs for Meat (Deal: +29% change) and MeatX (No deal: +80% change). The resulting updated target node distributions, due to conditionalizing Meat costs, are shown in black, the original model unconditionalized distributions are shown in grey.

For the what-if scenario where Meat prices are anchored to their projected 5th percentile values (Figure A2), the resulting Basket distribution shapes are bulk shifted to lower values, while those for the 95th percentile sensitivity test are moved higher (as might be expected, given certain other food prices are correlated with Meat price). Moreover, the standard deviations on the means are universally smaller under these Meat price sensitivity tests, and kurtosis of the distributions is reduced. In this hypothetical situation, because of the correlations structure in the Bayes Net, uncertainty distributions relating to the selected Baskets' percentage changes and monetary costs may be more Gaussian if future Meat prices were better constrained. However, the opposite may apply with other foods or combinations of foods; only detailed sensitivity testing will reveal how the estimates might change under different circumstances.

Gaining new or better information about individual contributory food price trajectories can radically change inferences about target item forecast distributions; focussing only on mean values can conceal or distract from meaningful effects, especially in patterns of tail behaviour. And, when we are dealing with multiple variables – here just ten food categories – the interplay

between factors and their uncertainties will be almost impossible to evaluate reliably, without the assistance of suitable numerical software. There are always likely to be subtle, sometimes counter-intuitive, relationships lurking in the complexities of any stochastic model of this nature.

The changes in the CPI Basket sub-food costs, expressed as percentages, appear numerically smaller than the corresponding changes if monetary costs are used. This is likely due to the weights that are applied to the different ways the various Baskets are composed. For instance, under the scenario of No deal and Meat price being set to its 95th percentile, the increase in mean percentage change for the CPI Basket is less than a factor 2x (+44% -v- +24%), whereas the monetary cost increase is more than a factor 3x greater (£44.84 -v- £13.97). Similar differences in sensitivity are present at the distribution 95th percentile levels.

Basic dependence structuring is added into the net to reflect the likely main correlations in prices that exist in reality. The converse, and frequently adopted, assumption -- that probabilistic model parameters are independent when in fact they are correlated -- introduces the risk of under-estimating the extent of joint distribution tails. At this stage, we do not claim that the correlation structures included in our Bayes Net model are comprehensive or numerically precise -- formally determining these dependencies requires further work. This said, the influence links in our net (i.e. arrows carrying correlation values) serve to offset, to some extent, the fallacious assumption that all the food price factors can be regarded as independent of one another.

To map the entire landscape of various possible Brexit-related food price projections, a full range of similar what-if sensitivity tests would be needed, including compound pricing evaluations for multiple combinations of foodstuffs, and for all, jointly; such an exercise lies outside the scope of the present analysis.
