# Supplementary material for: Anticipated impacts of Brexit scenarios on UK food prices and implications for policies on poverty and health: a structured expert judgement approach
Source: BMJ Open. 2020 Mar 3;10(3):e032376. doi: 10.1136/bmjopen-2019-032376 (PMC7059525; doi:10.1136/bmjopen-2019-032376)
Supplement: Author's manuscript [file bmjopen-2019-032376.draft_revisions.pdf]

BMJ Open is committed to open peer review. As part of this commitment we make the peer review history of every article we publish publicly available.

When an article is published we post the peer reviewers' comments and the authors' responses online. We also post the versions of the paper that were used during peer review. These are the versions that the peer review comments apply to.

The versions of the paper that follow are the versions that were submitted during the peer review process. They are not the versions of record or the final published versions. They should not be cited or distributed as the published version of this manuscript.

BMJ Open is an open access journal and the full, final, typeset and author-corrected version of record of the manuscript is available on our site with no access controls, subscription charges or pay-per-view fees (http://bmjopen.bmj.com).

If you have any questions on BMJ Open's open peer review process please email info.bmjopen@bmj.com

BMJ Open

Anticipated impacts of Brexit scenarios on UK food prices using structured expert judgement: implications for policies on poverty and health

Journal:	*BMJ Open*
Manuscript ID	bmjopen-2019-032376
Article Type:	Research
Date Submitted by the Author:	19-Jun-2019
Complete List of Authors:	Barons, Martine; University of Warwick<br>Aspinall, Willy; University of Bristol
Keywords:	Food poverty, PUBLIC HEALTH, chronic disease, Malnutrition, Child poverty

SCHOLARONE™
Manuscripts

I, the Submitting Author has the right to grant and does grant on behalf of all authors of the Work (as defined in the below author licence), an exclusive licence and/or a non-exclusive licence for contributions from authors who are: i) UK Crown employees; ii) where BMJ has agreed a CC-BY licence shall apply, and/or iii) in accordance with the terms applicable for US Federal Government officers or employees acting as part of their official duties; on a worldwide, perpetual, irrevocable, royalty-free basis to BMJ Publishing Group Ltd ("BMJ") its licensees and where the relevant Journal is co-owned by BMJ to the co-owners of the Journal, to publish the Work in this journal and any other BMJ products and to exploit all rights, as set out in our licence.

The Submitting Author accepts and understands that any supply made under these terms is made by BMJ to the Submitting Author unless you are acting as an employee on behalf of your employer or a postgraduate student of an affiliated institution which is paying any applicable article publishing charge ("APC") for Open Access articles. Where the Submitting Author wishes to make the Work available on an Open Access basis (and intends to pay the relevant APC), the terms of reuse of such Open Access shall be governed by a Creative Commons licence – details of these licences and which Creative Commons licence will apply to this Work are set out in our licence referred to above.

Other than as permitted in any relevant BMJ Author's Self Archiving Policies, I confirm this Work has not been accepted for publication elsewhere, is not being considered for publication elsewhere and does not duplicate material already published. I confirm all authors consent to publication of this Work and authorise the granting of this licence.

For peer review only

Anticipated impacts of Brexit scenarios on UK food prices using structured expert judgement: implications for policies on poverty and health

Martine J Barons[1] [0000-0003-1483-2943]• Willy Aspinall[2] [Orcid 0000-0001-6014-6042]
[1]Director of the Applied Statistics & Risk Unit Applied Statistics & Risk Unit, Department of Statistics, University of Warwick, Coventry, UK. Martine.Barons@warwick.ac.uk Corresponding author.
[2] Principal Consultant, Aspinall and Associates, Tisbury, and University of Bristol, Bristol, UK Willy.Aspinall@bristol.ac.uk

Abstract

Introduction Food insecurity has been associated with increased risk for several health conditions and poor management of chronic disease. Key determinants for household food insecurity are income and food costs. Whereas short-term household incomes are likely to remain static, increased food prices would be a significant driver of food insecurity.

Objectives To investigate food price drivers for household food security and health consequences in the UK under Brexit deal and no deal scenarios. To quantify the uncertainties by estimating the 5% and 95% quantiles of the price distribution.

Design Structured expert judgement elicitation, a well-established method for quantifying uncertainty, using experts. Each expert estimated the median, 5% and 95% quantiles of changes in ten food categories under Brexit deal and no-deal scenarios. These were aggregated based on the accuracy and informativeness of the experts on calibration questions.

Participants 10 experts with expertise in food procurement, retail, agriculture, economics, statistics and household food security.

Results Expected changes in food costs varied between categories. When combined in proportions used to calculate CPI, median food price changes for Brexit with a deal are expected to be +6.1% [90% credible interval:-3%, +17%] and with no deal +22.5% [+1%, +52%].

Conclusions The number of households experiencing food insecurity and the severity of food insecurity is likely to increase since the median food cost increases expected after Brexit are significant. The increasing burden on healthcare services is likely to increase sharply. Moreover, the uncertainty in food costs is skewed, making higher increases more likely than lower rises. The plausible worst case would entail severe impacts. The demand for health services in both the short and longer term is likely to increase due to the effects of food insecurity on disease incidence, management of chronic conditions, amplifying the involvement of physicians in referral to emergency food relief.

Keywords Brexit • Food prices • Consumer Price Index • Structured expert judgement • Uncertainty

Strengths limitations of this study

- First study to quantify anticipated food price changes and associated uncertainty relating to Brexit Deal and No deal scenarios using a transparent and established protocol, and to articulate links to potential healthcare impacts.
- Inclusion of experts with broad and overlapping areas of expertise in food production and supply.
- Sensitivity analysis showing how price changes in linked food categories could combine to affect overall food costs.
- Fewer than optimal number of experts elicited.
- Study was undertaken on the assumption that Brexit would occur on 29th March 2019. Delays and major ambiguities with respect to eventual Brexit circumstances have emerged since experts made projections in 2018.

INTRODUCTION

Food insecurity, the lack of access to sufficient nutritious food, is associated with multiple negative outcomes including several chronic diseases(1-6), poor educational attainment, poor mental health and social isolation, which increases mortality(7). The medical importance of a basic nutritional safety net has long been recognised by policymakers through the Welfare Food Scheme and Healthy Start (HS) programmes. A recent systematic review identifies poor diet quality as an important preventable risk factor for non-communicable disease, responsible for one-in-five deaths globally and 127 deaths per 100,000 Britons(8). Under post-2008 austerity measures, cuts have been made to HS and other provisions. The UK's main response to growing food insecurity has been charitable food relief, but the efficacy of these and similar approaches is unmeasured. Social protection spending and welfare state interventions are the only actions known to alter the prevalence of household food insecurity(9). The rise in food bank use is attributed largely to welfare cuts(10). Despite the purported end of austerity, the inability of some households to feed themselves adequately persists. Figures from the Trussell Trust, the UK's largest network of foodbanks, show 658,048 3-day emergency food supplies were issued in the 6 months to September 2018, an increase of 13% over the same period in 2017[1].

An important driver of household food security is the costs of food and other essentials relative to incomes. Absolute income levels and volatility are both important (11, 12). There was little growth in real earnings in 2017–18, and the Office for Budget Responsibility forecast slow earnings growth for the next four years(13). This means that the main driver of household food security will be food price. UK price inflation is measured by changes in the UK Consumer Prices Index (CPI). The CPI is based on a 'shopping basket' of goods and services, including a food element. In November 2018, the CPI inflation was 2.3% p.a. over all items and 0.5% p.a. for the food element[2]. The CPI is based on actual consumer spending and the food element incorporates both spending on healthy nutrition and on less healthy options.

[1] Trussell Trust T. End of Year Stats. 2018.
[2] Consumer Price Inflation Table 22 [Internet]. Office for National Statistics. 2019 [cited 11/01/2019]. Available from: https://www.ons.gov.uk/economy/inflationandpriceindices/datasets/consumerpriceinflation.

For peer review only - http://bmjopen.bmj.com/site/about/guidelines.xhtml

In 2016, the UK voted to relinquish its membership of the European Union (EU), known colloquially as 'Brexit', to be completed by 29 March 2019. UK reliance on food imports, including from EU, is significant. Almost one-half of the UK's food is imported: 30% comes from the EU, and another 11% comes from non-EU countries under the terms of trade deals negotiated by the EU. Prices of fruit and vegetables are particularly vulnerable to vagaries of production and supply(14). In estimates of the economic impact of Brexit on the UK, the least damaging scenarios are those which are closest to the current situation under EU membership (i.e. retaining membership of the Single Market and Customs Union), while a 'no-deal' scenario is predicted to be the most damaging(15). In anticipation of Brexit in March 2019, we conducted an elicitation of a group of experts in July 2018 on their expectations for possible impacts of two Brexit deal scenarios on food prices over the 14 months following Brexit (i.e. to June 2020).

One of the motivations for investigating possible impacts of Brexit on food prices is that, over the last few years, medical General Practitioners in the UK have raised concerns about food insecure patients seeking referrals to food banks[3] and that food insecurity is affecting medication compliance, health and wellbeing[4]. This has raised concern about resource implications for surgeries (BMJ, 2018) and that "the welfare system is failing to provide a robust last line of defence against hunger"(16).

A 2017 survey showed 13% of people were worried that their food would run out before they got money to buy more ('marginally food secure households') and 8% couldn't afford to eat balanced meals or went hungry ('low or very low food secure households')(17) . In low income households, 29% experience food insecurity(18). Lower-income households inevitably allocate a higher proportion of spending on food, and buy a similar fraction of imported food; therefore, low-income households are more exposed to food price rises. In November 2018 the United Nations OHCHR special rapporteur issued a statement in which he argued that the rising use food banks in the UK is a consequence of poverty, including in-work poverty. He recommended that the UK government should settle on a single measure of poverty and should begin to measure and monitor food security (19).

In setting a comprehensive strategy for the UK to ensure household food security, policymakers must grapple with how to prioritise low food prices, animal welfare, minimum income, health, welfare and social protection.

METHODS

A projection of food prices gives a key insight into the effects of uncharted events on household food security and it health consequences. As far as we know, no projections have been published for the impact on food prices or CPI of Brexit which enumerate associated uncertainties formally. We report key findings from the application of a structured expert judgement (SEJ) elicitation to potential food price changes and their uncertainties in the event of Brexit under two scenarios: 'deal' and 'no-deal'. By Brexit deal we mean with trading arrangements broadly similar to the present. With Brexit no-deal we mean that such arrangements will be discontinued and individual trade deals would need to be negotiated. Our

[3] Pulse. One in six GPs asked to refer a patient to a food bank in the past year. Pulse: Pulse; 2014.

[4] Guardian. 'It's a life or death situation': why GPs are referring patients to food banks: The Guardian; 2018 [Newspaper]. Available from: https://www.theguardian.com/healthcare- network/2018/feb/09/life-death-situation-why-gps-referring-patients-food-banks

panel (see Acknowledgments) had expertise in food procurement, retail, agriculture, economics, statistics and household food security, and each expert considered potential impacts of the Brexit scenarios on prices separately for each of ten food categories that are used in the UK Consumer Prices Index (CPI).

Structured expert judgement

Structured expert judgement (SEJ) elicitation provides a formal approach for estimating uncertain quantities according to current professional knowledge and understanding, and has been utilised in a wide range of applications. To amalgamate our experts' judgements, we used Cooke's Classical Model, a well-established, validated approach (20-23). Cooke's method uses a mathematical scoring rule basis to evaluate empirical performance-based weights for aggregating individual experts' judgements. This means that when all the individual estimates are combined, the experts who were most informative and most accurate on a set of related calibration questions (to which only the analysts had the answers) contributed most to the final estimates of the questions of interest.

The independent facilitator (WA) devised calibration questions based on historic food price changes. The questions of interest were future food price changes. We began by discussing with the group the wordings and meanings of the questions, to minimise ambiguities or misunderstandings. The experts themselves then clarified and revised several of the target questions.

For both calibration questions and target questions, we asked the experts to integrate, within their judgements, all the factors and circumstances under which food prices could be driven up or down. Each expert provided their own estimates, confidentially to the facilitator (WA), for the lowest plausible, highest plausible and best estimate for price change for each food category under the specific scenario. We discussed with the experts that we would treat their three judgement values for each item in the subsequent analysis as analogous to a 90% credible interval range, with their best estimate value representing the median of their uncertainty spread. We made clear that a median value need not necessarily be central to the credible interval if, in the expert's judgement, the relevant uncertainty distribution is not symmetric and should exhibit skewness (either to higher or lower values).

This confidential elicitation procedure encourages participants to express judgements based on their true beliefs, reducing potential sources of bias due to peer- and group influences. We also maintained anonymity when presenting the individual judgements and weights derived from the calibration questions.

Analysis of Elicitation

We aggregated mathematically individual uncertainty distributions for each food category to construct food price change probability density functions, using the performance-based weights derived from the expert calibration step in the Cooke's Classical Model (20).

For peer review only

We processed our experts' responses using the program EXCALIBUR[5] (24), which computes weighted combinations of judgements and produces a synthesis for each food item expressed in terms of the same three quantiles used in the elicitation (i.e. 5th; 50th and 95th percentiles). We report in the next section both the CPI weighted combinations and the family healthy basket combinations (based on McMahon and Weld (2015)(25)).

Computing Brexit-related Food Basket Cost Changes

In order to estimate price change distributions for different shopping baskets under the two Brexit scenarios we use the Bayes Net (BN) code UNINET[6] (26). BNs are graphical tools for representing and computing high dimensional joint uncertainty distributions (27, 28). Our BN for calculating food basket price changes post-Brexit is shown in Figure 1.

Recognising the prices of certain foodstuffs are subject to common influences or are related to one another through production and processing, we introduced some basic dependence structure to reflect the likely main correlations that are present in food prices. The converse assumption, that food prices are independent, introduces the risk of under-estimating the joint extent of interrelated changes on distribution tails.

Vegetable and fruit prices are correlated by a mutual dependency on weather conditions. However, both within and between food categories there can be differential effects on different crops: for example, spring floods which rot potatoes and delay grain sowing do no harm to orchards, which suffer if there is an unseasonal frost as the fruit is setting. We set this correlation at about +0.75

Corn and sugar beet are used as feed for cattle, inducing a correlation between meat and grain (bread), and between meat and sugar; we set these correlations each at +0.4. While dairy and beef cattle markets are quite distinct, their prices are linked through a common dependency on feed costs, so we link milk and meat with a correlation +0.4.

Grain (bread) and sugar (beet) can both be feedstock for biofuel and thus are linked via oil price; to account for this and other, secondary joint correlations, we set this correlation to +0.72. In fact, all food prices are linked to oil price through production and transport costs, but these two more strongly.

The 2016 'sugar tax' saw food producers make a substantial switch to artificial sweeteners, smaller serving sizes etc., to avoid significant price rises being passed to the consumer. In light of this, we set the correlation between sugar and soft drinks to a relatively low value of +0.3.

[FIGURE 1 HERE]

Figure 1: UNINET Bayes Net for calculating joint overall percentage change in CPI Basket from elicited sub-Food categories (factored with ONS weights: node "Wts") and changes in Food Baskets costs. Upper sub-net: for Brexit with- deal Brexit; lower sub-net: for no-deal Brexit scenario. Food category nodes are labelled Bread, Meat.. etc for with-deal scenario, and BreadX, MeatX, etc for no-deal. The food category ellipse nodes contain the expert group weighted combinations of percentage price change uncertainty distributions per sub-Food category. Target nodes (large ellipses) are weighted sums over the ten selected food category PDFs, expressed

[5] Program available at www.lighttwist.net/wp/excalibur
[6] Program available at www.lighttwist.net/wp/uninet

For peer review only

in terms of CPI Food Basket percentage change (left pair of target nodes) and the cost changes for the CPI Food Basket and for two typical consumer food basket types. Arrows with numbers attached denote correlated factors (see text).

RESULTS

We present estimated projected price changes by June 2020, assuming Brexit on 29th March 2019, using food price change quantiles elicited at food category level (Table 1). We use the BN in Figure 1 to combine these price changes and associated uncertainties for the food element of the CPI basket, and to estimate monetised equivalents for specific family types based on McMahon and Weld (2015). We discuss the likely effects on health and the demand for health services. The results of the analysis are in Table 1 and are shown graphically in Figure A1.

	Food category percentage price changes by June 2020 median (5th, 95th percentile)	
**CPI category**	**Brexit deal**	**Brexit no-deal**
Soft drinks etc.	6 (0, 26)	8 (0, 47)
Coffee, tea & cocoa	2 (-9, 19)	4 (-5, 69)
Sugar, jam, etc.	7 (-9, 20)	19 (-5, 82)
Vegetables	3 (-10, 20)	9 (-18, 63)
Fruit	5 (-10, 24)	16 (-8, 51)
Oil & fats	5 (-9, 20)	18 (-8, 87)
Milk, cheese & eggs	6 (-9, 20)	23 (-5, 82)
Fish	4 (-9, 19)	5 (-13, 41)
Meat	6 (-10, 29)	18 (-11, 80)
Bread & Cereals	4 (-9, 19)	10 (-7, 83)
**Overall % change ONS CPI sub-Foods, with category weights**	**Mean +6.4% ± 6.0 Median +6.1% [-2.7, +16.9]**	**Mean +24.0% ± 15.4 Median +22.5% [+1.49, +51.7]**
	**Food basket cost changes by June 2020 (in £'s)**	
**Change in CPI weekly cost relative to 2018 year end Basket total £58.00***	**Mean +£3.78 ± £3.76 Median +£3.53 [-£1.90, +£10.41]**	**Mean +£13.97 ± £9.52 Median +£13.00 [+£0.08, +£31.02]**
**Change in family of 4 Healthy Food Basket basis weekly cost £93.56****	**+£6.30 ± £6.71 +£5.80 [-£3.68, +£18.17]**	**+£22.58 ± £16.14 +£20.98 [-£1.07, +£50.98]**
**Change in single pensioner Healthy Food Basket basis weekly cost £35.44****	**+£2.28 ± £2.56 +£2.09 [-£1.51, +£6.80]**	**+£8.11 ± £6.23 +£7.55 [-£1.05, +£18.99]**

*Table 1 Aggregated food prices change estimates. "Brexit deal" means a deal similar to the present arrangements will be implemented, so little disruption or additional costs to supply routes. "Brexit no-deal" means that such arrangements will be discontinued and individual trade deals would need to be negotiated. Numerical values are medians (90% credible intervals). *Based on ONS Table A2 2018 year end data (Mar 2018):*

For peer review only

*selected Basket sub-Food category weekly costs; total for the ten items = £58.00. **Based on MacMahon and Weld (2015) Northern Ireland minimum essential Healthy Basket sub-Food category weekly costs at November 2014 Tesco prices. For two adults and two children, one in pre-school (aged 2-4) and one in primary school (aged 6-11), total cost for the ten items = £93.56; for a single pensioner, the corresponding selected items cost = £35.44.*

Using the ONS food category weightings, we aggregate their contributions to a CPI basket. We see that, under a Brexit deal, this gives expected median food price rises around +6% (i.e. a rise 12 times higher than in 2018) by June 2020, with a plausible, i.e. 1-in-20 (5%) likelihood of a drop in prices of about -3% or more and a 1-in-20 chance of a rise of +17% or more. Under Brexit no-deal, the overall median food price escalation is expected to be +23%, with a lower plausible increase of about +1% and upper plausible increase of about +52% (again, each bound has a 1-in-20 chance of being exceeded).

Thus, the foreseeable most likely outcome of Brexit is significant price rises. This will lead to more household food insecurity and its attendant deterioration in health and increase in demand for health services.

What-if scenario sensitivity testing

We wished to investigate how sensitive the overall basket food cost results are to a single food type. Currently the only UK differentials from global prices are beef and poultry, where the EU's production standards are higher than the rest of the world[7]. This suggests that constraining price rises for these foods following Brexit might be achieved principally by lowering animal welfare and food hygiene standards, with associated risks to human health.

We set cost of meat to its 5th percentile level and then its 95th percentile level and investigated the effects on the overall food basket cost, with other food types unchanged. The resulting changes to the CPI Food Basket and to the Family Basket cost projections reported in Table 2 and Figures A2 and A3.

**Bayes Net food costs estimates: what-if scenario analytical conditioning**				
Scenario	CPI Basket sub-foods cost: mean percentage change*	Family basket: mean cost change*	CPI Basket sub-foods cost: 95%ile percentage change*	Family basket: 95%ile cost change*
Meat price → projected 5th percentile cost				
**Deal** [Meat cost -10%]	-0.1% (+6.4%)	-£1.26 (+£3.78)	+6.2% (+16.9%)	+£5.01 (+£10.41)
**No deal** [Meat cost -11%]	+8.6% (+24.0%)	+£5.71 (+£13.97)	+24.4% (+51.7%)	+£21.13 (+£31.02)
Meat price → projected 95th percentile cost				
**Deal**	+13.5% (+6.4%)	+£14.66 (+£3.78)	+20.6% (+16.9%}	+£21.61 (+£10.41)

[7] Farmers Weekly. Opinion: Cheap food after Brexit is an illusion. Farmers Weekly 2018.

For peer review only - http://bmjopen.bmj.com/site/about/guidelines.xhtml

[Meat cost +29%]				
**No deal** [Meat cost +80%]	+44.0% (+24.0%)	+£44.84 (+£13.97)	+62.2% (+51.7%)	+£61.56 (+£31.02)

Table 2 Example impacts on CPI and Family Food Basket costs from analytical conditioning the Bayes Net to the 5th percentile and 95th percentile projected costs of Meat, under Brexit Deal and No deal scenarios.
* Corresponding base model results are shown in brackets.

With meat cost at 5th percentile levels in the elicitation distributions (-10% and -11%, deal & no-deal respectively, see Table 1), the CPI Basket mean change for Brexit deal becomes -0.1% (cf +6.4%) and the Family Basket mean cost changes from +£3.78 to -£1.26. Under the Brexit no-deal scenario the CPI Basket mean change would be +8.6% (cf +24.0%) and, for the Family Basket, the cost increase becomes +£5.71 (c.f. +£13.97).

With meat cost at 95th percentile levels from the elicitation deal and no-deal distributions (+29% and +80%, respectively) the CPI Basket mean change for Brexit deal increases from +6.4% to + 13.5% and the Family Basket mean cost rises from +£3.78 to +£14.66. For the Family Basket, the cost rises to +£44.84 (c.f. +£13.97).

The mean (expected) CPI Basket and Family Basket cost changes would be kept close to zero only if Meat prices were, somehow, limited to near their projected 5th percentile levels -- and, with them, the price changes of foods correlated with Meat were also curbed -- and then only under the Brexit Deal scenario. Otherwise, higher Meat prices will inevitably amplify basket cost changes. The standard deviations on the means are smaller under these Meat price sensitivity tests, and the correlation structure reduces the kurtosis of the distributions.

Although there are some notable differences in the item price compositions of the CPI food basket and the family 'healthy' food basket (e.g. lower spend in UK CPI basket on meat: £12.80 - v- £30.18 per week for Northern Ireland), our analysis shows that overall cost percentage changes in these two representative household food baskets differ little: for Brexit no-deal, both estimates represent about +22% increases in projected mean costs.

We selected meat for this sensitivity analysis as it the most likely to change in price depending on the details of any trade deals agreed. For other foods or combinations of foods, the opposite may apply; similar sensitivity analyses are possible.

DISCUSSION

Food price rises after Brexit are likely to be significant and may be substantial. In the light of the expected stagnation of household incomes, this is likely to drive more households into food insecurity. Food is a substantial portion of household expenditure, especially in low-income households. MacMahon and Weld (25) found that, after housing and childcare costs, the highest category of household expenditure in Northern Ireland is on a minimum essential 'healthy' food basket. They found food costs to be more expensive in rural areas for all household types and the highest spend was on meat, followed by the category fruit and vegetables. Those households buying most would, of course, incur the greatest actual spend

For peer review only

increases, with concomitant implications for affordability in terms of differing household-related incomes.

One strength of our analysis is that the expert judgement median estimates we obtain by elicitation are consistent with central estimates produced by UK Trade Policy Observatory and by the British Retail consortium[4]. However, we add further information for decision support by presenting quantified uncertainties around our estimates. These spreads can be substantial and all exhibit skew in the form of extended, 'heavy' upper tails – i.e. larger price increases are more likely than smaller increases (or reductions). Related decision-making that is based only on central (average) estimates and neglects these uncertainties can lead to poor policy selection (29).

Policymakers benefit from consideration of a defined reasonable worst case for contingency planning, and are proficient at interpreting probabilistic statements, which help clarify underlying assumptions(30). Moreover, historic vegetable CPI rises offer a perspective for the elicited credible intervals for CPI vegetable price change in Table 1. The largest historic one-year change in vegetable prices since 1987 was +14.8% (2006-7) and the largest two-year jump was +27.1%, for the period 2006 to 2008. Compared to the Brexit deal scenario, the record two-year rise is greater than the elicited 95th percentile vegetable price index change (i.e. +20% by June 2020, two-years ahead from the elicitation), but falls within the Brexit no-deal 90% credible interval, for which the projected 95th percentile change could exceed +63%. One weakness of the study was that we had fewer than the optimal number of experts. Whilst the spread of expertise was good, a wider spread of expertise could have improved quantification of price changes, possibly narrowing the uncertainty around the central estimates.

Other attempts to quantify food prices after Brexit have not been set within the context of health and healthcare provision. Whilst the experience of food security is largely driven by the cost of food and other essentials relative to incomes, other factors such as self-efficacy, access to credit and other forms of social capital are also significant (cite Loopstra thesis).

The implications for health, healthcare costs and clinician workloads are clear – unless the rising tide of food insecurity is reversed, health costs will continue to rise. Our findings should alert policymakers to the potential for significant increases in food costs under either Brexit scenario, with major impacts likely to follow a no-deal outcome. The expected levels of these increases and, more importantly, the uncertainty spreads on the estimates - all of which are skewed moderately toward higher costs - should inform policies that allow households to afford minimum essential food baskets, meeting acceptable physical, psychological and social needs. One likely corollary to substantial post Brexit food price rises is even greater consumption of cheaper, less healthy diets, with inevitable impacts on population long-term health trends and demands on the NHS. Medical practitioners and health care workers are amongst those who will have to confront the related challenges if food prices rise sharply and substantially after Brexit. Clinicians may need to organise processes to cope with the demand from food insecure patients for referrals to foodbanks. The management of chronic conditions may deteriorate, increasing the need for intervention. Food insecurity at present may also increase healthcare demand in the longer term. Policies that support incomes and access to food are the only interventions that have a demonstrable positive effect on food insecurity. It is incumbent on policymakers to ensure that the last line of defence against hunger is robust in order to promote well-being, productivity and healthy ageing.

For peer review only

Current evidence on the efficacy of some of the most widespread current responses to food insecurity, such as foodbanks, is limited. More research is needed in order to select the most effective strategies for supporting health and well-being. While Brexit did not take place by the due date, and the overall political situation is now even less clear than before, certain aspects of potential impacts on food supplies have become more explicit. For example, the impact of stockpiling on non-perishable food prices can be now ascertained more accurately, as those costs can be quantified. In other respects, however, uncertainties associated with post-Brexit food pricing are likely to have increased. For instance, at the time of writing (June 2019), the current target date for Brexit has moved to 31 October 2019, and might be postponed even later. Inevitably, the precise timing of Brexit will have an impact on the prices of fresh foods due to the seasonal nature of such produce.

Given almost everything concerning Brexit is currently in a state of flux, it is our intention to conduct an updating elicitation, once the circumstances surrounding how Brexit will be implemented become clearer. Experts who participated in our 2018 elicitation have expressed a universal interest in re-visiting the issues and factors, and in reviewing their judgements of future food prices and the related uncertainties. We will consider increasing membership of the panel of experts to obtain an even wider sample of judgements. One advantage of this repeat elicitation would be the opportunity to investigate how much the quantified uncertainties, reported here, may have changed. When we know the terms of Brexit, an elicitation update will be able to quantify possible adjustments to the present estimates. In addition, we intend to explore in more quantitative detail possible underlying correlations between prices of different foodstuffs. New advances in elicitation techniques for assessing parameter dependences (31) offer a ratifiable basis for quantifying the properties of such correlations. It seems inevitable that the implications of an eventual Brexit for projected food price forecasts will be fundamental to, and crucial for policy planning in many societal and political areas.

For peer review only

Acknowledgements

We acknowledge with gratitude the invaluable contributions of the experts who participated in the elicitation workshop, 4-6 July 2018:

Martine J. Barons (University of Warwick, Department of Statistics)
Steve Brewer (University of Lincoln, Lincoln Institute for Agri-Food Technology (LIAT), Network Coordinator for the Internet of Food Things Network Plus)
Rosemary Collier (University of Warwick, Life Sciences, Crop Centre)
Peter Crosskey (freelance food journalist, formerly of The Grocer)
Jonathan Horsfield (retired from Warwickshire County Council; energy expert)
Jim Q Smith (University of Warwick, Department of Statistics)
Thijs van Rens (University of Warwick, Department of Economics)
Rachel Wilkerson (University of Warwick, Department of Statistics)
Sophia Wright (University of Warwick, Department of Statistics)

Other participating experts from industry preferred to remain anonymous.

Funding The workshop was funded by the Warwick Global Research Priority for Food. The study is part of work undertaken for EPSRC grant number EP/K007580/1.

Competing interests None declared.

Contributions MJB conceived the study and organised the workshop and experts' participations. WA advised MJB on the elicitation and acted as a neutral independent facilitator for the elicitation and processed the experts' responses. MJB provided information for contextualising the elicitation findings, and both authors jointly wrote the paper.

Ethics approval; Patient and Public Involvement Not applicable.

Provenance and peer review Not commissioned; external peer reviewed.

Data sharing statement The first author may be contacted for requests for data or information used in this study.

Inclusion criteria The study did not use or rely directly on other published data.

Open access This is an Open Access article distributed in accordance with the terms of the Creative Commons Attribution (CC BY 4.0) license, which permits others to distribute, remix, adapt and build upon this work, for commercial use, provided the original work is properly cited. See: http://creativecommons.org/licenses/by/4.0/

For peer review only

REFERENCES

1. Seligman HK, Laraia BA, Kushel MB. Food Insecurity Is Associated with Chronic Disease among Low-Income NHANES Participants. The Journal of Nutrition. 2009;140(2):304-10.
2. Townsend MS, Peerson J, Love B, Achterberg C, Murphy SP. Food Insecurity Is Positively Related to Overweight in Women. The Journal of Nutrition. 2001;131(6):1738-45.
3. Vozoris NT, Tarasuk VS. Household Food Insufficiency Is Associated with Poorer Health. The Journal of Nutrition. 2003;133(1):120-6.
4. Laraia BA. Food Insecurity and Chronic Disease. Advances in Nutrition. 2013;4(2):203-12.
5. Hampton T. Food Insecurity Harms Health, Well-being of Millions in the United States. JAMA. 2007;298(16):1851-3.
6. Seligman HK, Bindman AB, Vittinghoff E, Kanaya AM, Kushel MB. Food Insecurity is Associated with Diabetes Mellitus: Results from the National Health Examination and Nutrition Examination Survey (NHANES) 1999–2002. Journal of General Internal Medicine. 2007;22(7):1018-23.
7. Steptoe A, Shankar A, Demakakos P, Wardle J. Social isolation, loneliness, and all-cause mortality in older men and women. Proceedings of the National Academy of Sciences. 2013:201219686.
8. Afshin A, Sur PJ, Fay KA, Cornaby L, Ferrara G, Salama JS, et al. Health effects of dietary risks in 195 countries, 1990–2017: a systematic analysis for the Global Burden of Disease Study 2017. The Lancet. 2019;393(10184):1958-72.
9. Loopstra R. Interventions to address household food insecurity in high-income countries. . Proceedings of the Nutrition Society,. 2018; 77(3):270-81.
10. Rachel Loopstra, Aaron Reeves , David Taylor-Robinson , Ben Barr, Martin McKee, David Stuckler Austerity, sanctions, and the rise of food banks in the UK. British Medical Journal. 2015;350.
11. Laura Leete NB. The effect of income shocks on food insufficiency. Review of Economics of the Household. 2010;8(4):505-26.
12. Jonathan Morduch RS. The Financial Diaries: How American Families Cope in a World of Uncertainty. 1 ed. Oxford, UK: Princeton University Press; 2017. 248 p.
13. Jonathan Cribb ANK, Tom Waters. Living standards, poverty and inequality in the UK: 2018. In: Studies TIfF, editor. 2018.
14. HOUSE OF LORDS European Union Committee. Brexit: food prices and availability. In: Committee HOLEU, editor. 2018.
15. Mott H. Exploring the Economic Impact of Brexit on Women Women's Budget Group; 2018.
16. Ashton JR, Middleton J, Lang T. Open letter to Prime Minister David Cameron on food poverty in the UK. The Lancet. 2014;383:1631-.
17. Beverley Bates CR, Hayley Lepps, Lauren Porter. The Food & You Survey Wave 4 Combined Report for England, Wales and Northern Ireland In: Agency FS, editor.: NatCen Social Research 2017.
18. Nelson M EB, Bates B, Church S, Bosher T. Low income diet and nutrition survey. Vol 3. Nutritional status, physical activity, economic, social and other factors. In: Food Standards Agency, editor. 2007.
19. Alston P. Statement on Visit to the United Kingdom, by Professor Philip Alston, United Nations Special Rapporteur on extreme poverty and human rights. In: Commissioner OotH, editor.: OHCHR; 2018.
20. Cooke RM. Experts in Uncertainty - opinion and subjective probability in science. New York: Oxford University Press; 1991 321 p.
21. Aspinall W. A route to more tractable expert advice. Nature. 2010;463:294-5.
22. Sutherland WJaMB. Comment: Policy advice: Use experts wisely. Nature 2015;526:317–8.
23. Colson AR, Cooke, R. M. . Cross validation for the classical model of structured expert judgment. . Reliability Engineering and System Safety 2017;163:109-20.
24. Cooke R, Solomatine D. EXCALIBR User's Manual. Delft, The Netherlands: Delft University of Technology/SoLogic; 1992.

For peer review only

25. MacMahon B, Weld, G. The cost of a minimum essential food basket in the Republic of Ireland. Study for six household types. . 2015.
26. Ababei D. UNINET. Software designed by the Risk and Environmental Modeling Group, Delft University of Technology. Developed by D. Ababei, Lighttwist Software. . 2016.
27. Jensen F, Jensen F, Nielsen T, Graven-Nielsen T. Bayesian Networks and Decision Graphs Second Edition ed. New York: Springer; 2010.
28. Smith JQ. Bayesian Decision Analysis, principles and practice. Cambridge: Cambridge University Press; 2010.
29. M. Leonelli JQS. Using graphical models and multi-attribute utility theory for probabilistic uncertainty handling in large systems, with application to the nuclear emergency management. IEEE International Conference on Data Engineering Workshop proceedings 2013

2013.
30. Stirling A. Keep it complex. Nature. 2010;468 1029–31.
31. Bamber JL, Oppenheimer M, Kopp RE, Aspinall WP, Cooke RM. Ice sheet contributions to future sea-level rise from structured expert judgment. Proceedings of the National Academy of Sciences. 2019;116(23):11195-200.

For peer review only

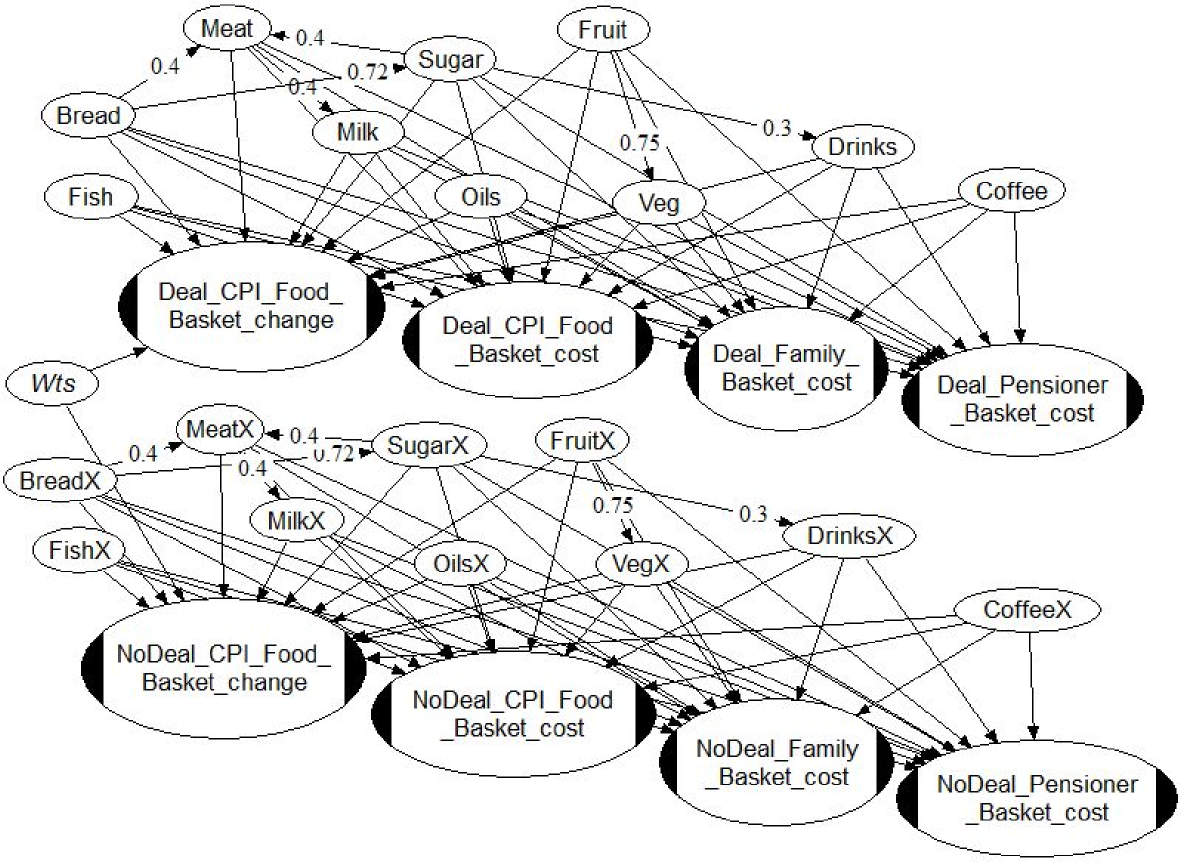
Meat
0.4
Sugar
Fruit
0.4
0.72
Bread
0.4
Milk
0.75
0.3
Drinks
Fish
Oils
Veg
Coffee
Deal_CPI_Food_Basket_change
Deal_CPI_Food_Basket_cost
Deal_Family_Basket_cost
Deal_Pensioner_Basket_cost
Wts
MeatX
0.4
SugarX
FruitX
0.4
0.72
BreadX
0.4
MilkX
0.75
0.3
DrinksX
FishX
OilsX
VegX
CoffeeX
NoDeal_CPI_Food_Basket_change
NoDeal_CPI_Food_Basket_cost
NoDeal_Family_Basket_cost
NoDeal_Pensioner_Basket_cost

Supplementary information

UNINET

UNINET enables efficient calculation of Bayes rule equations and rapid parameter conditionalization for making probabilistic inferences from observations. Conditionalizing a large network on multiple disparate observations and propagating this information through a multi-nodal model is analytically challenging; heuristics and simple statistical models can often lead us astray. BNs not only solve particular problems of information synthesis, they also provide a basis for reasoning probabilistically and testing model assumptions.

The main features of UNINET (distinguishing it from other packages) are that it supports non-parametric continuous or discrete BBNs, supplemented with functional nodes. Probabilistic nodes may be assigned arbitrary continuous or discrete distributions. UNINET has the valuable attribute of accepting Decision Maker results directly from EXCALIBUR output. Thus, Classical Model DM quantile histograms can be compiled into minimum information distribution form, without pre-processing, and used for propagating uncertainties into the desired analysis calculations.

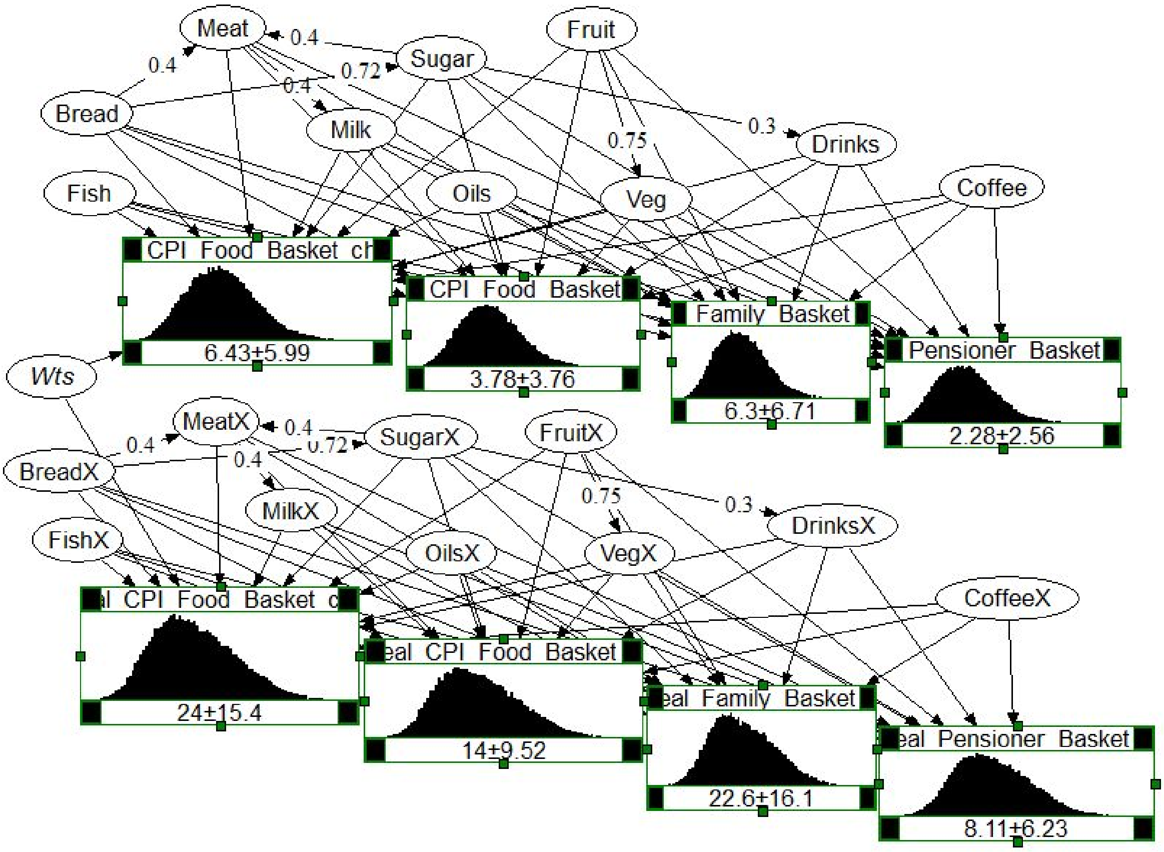

Figure A1 Bayes Net calculated output node distributions for percentage change in CPI Food Basket cost for Brexit Deal and No deal scenarios (LH pair of histograms) and for cost change in CPI Food Basket and two household cases in £, for the same Brexit scenarios. Each histogram panel reports the mean ± 1 s.d. change for that target item; other distribution statistics are output numerically by UNINET. Note hints of long upper tail skewness in every case (see text for discussion).

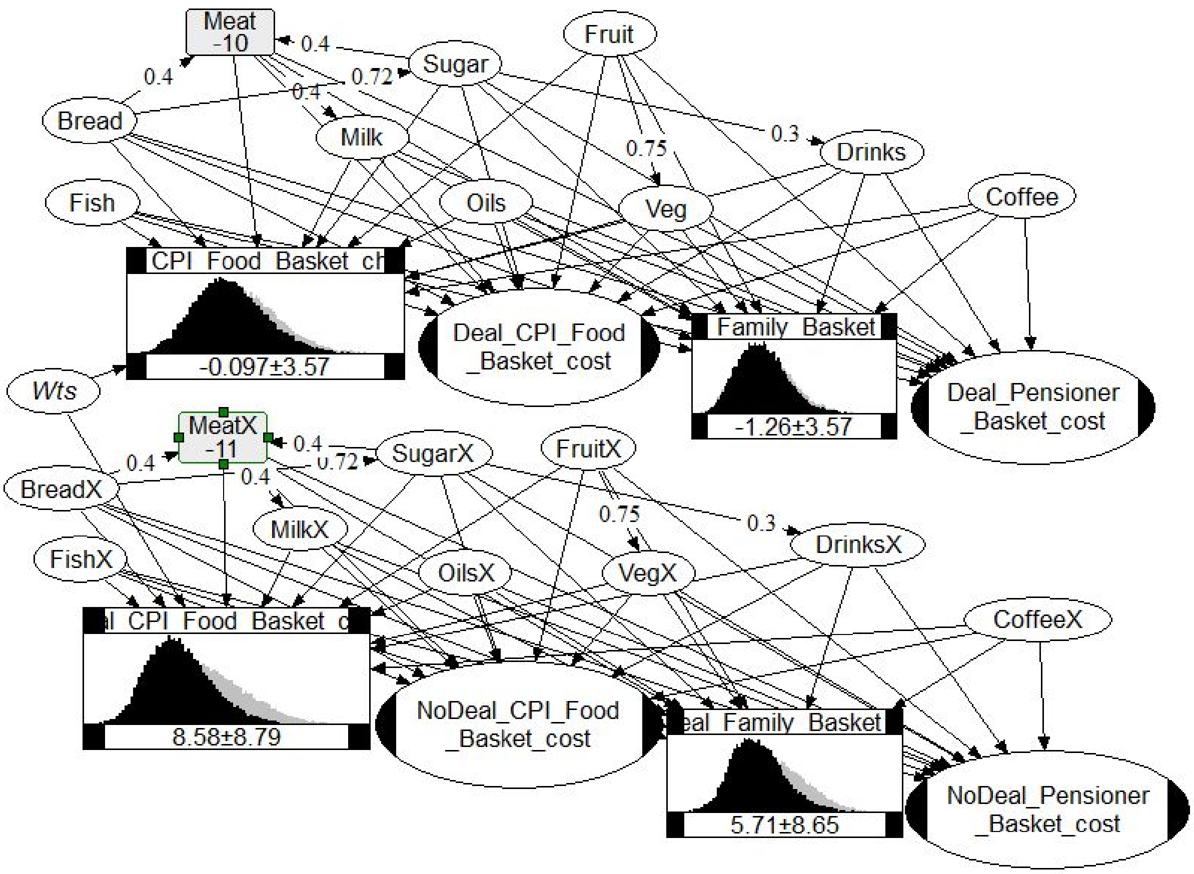

Figure A2 Bayes Net for Food baskets prices conditionalized on projected 5th percentile Meat (Deal: -10% change) and MeatX (No deal: -11% change) costs. The resulting updated target node distributions, due to conditionalizing Meat costs, are shown in black, the original model unconditionalized distributions are shown in grey.

A valuable attribute of UNINET, of particular benefit in the present study, is its capability to allow specific value conditioning on any number of probabilistic nodes. Analytic conditioning comprises either: (a) conditioning on a single value, which applies only for probabilistic nodes; or (b) sample-based conditioning, by which means the user can conditionalize on specific value intervals (for probabilistic and functional nodes). Thus, UNINET can model an empirical multivariate distribution by building a joint density function, which can be analytically conditioned. This capability is used here for conducting a pair of example sensitivity tests, based on selected Meat cost changes.

The UNINET BN is re-run with two food category nodes (deal Meat and no-deal MeatX) conditionalized to their 5th percentile levels from the elicitation distributions (-10% and -11%, respectively). In this example, we examine the effects of fixing these two nodes on the deal and no-deal outcome CPI basket percentage change and on the family-of-four basket cost, respectively. The resulting updated target node distributions, due to conditionalizing Meat costs, are shown on Figure A2.

Next, we run the BN with Meat (Deal) and MeatX (No deal) conditionalized to their elicited 95th percentile changes, i.e. +29% and +80%, which represent price increases well above the elicited medians, respectively (Figure A3). Under the Brexit no-deal scenario: the CPI Basket mean change would be +44.0% (cf +24.0%) %)

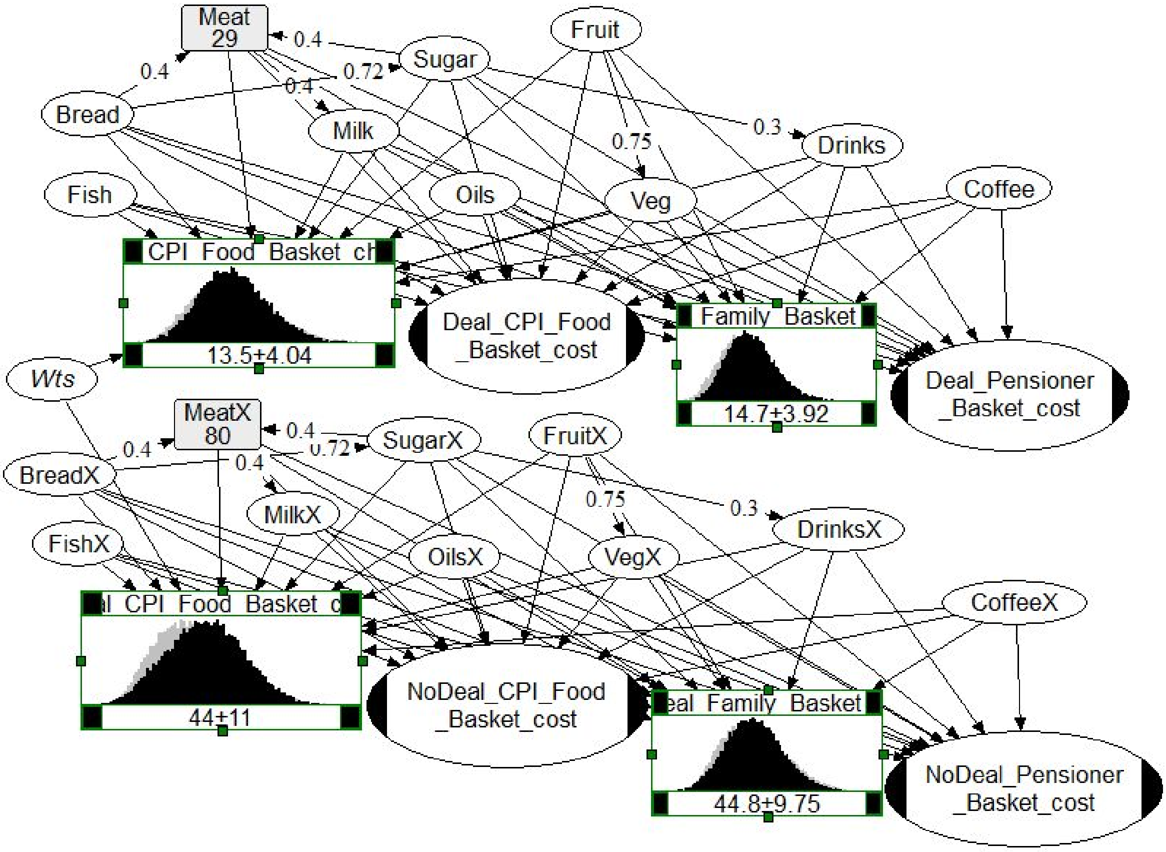

Figure A3 Bayes Net for Food baskets prices conditionalized on projected 95th percentile costs for Meat (Deal: +29% change) and MeatX (No deal: +80% change). The resulting updated target node distributions, due to conditionalizing Meat costs, are shown in black, the original model unconditionalized distributions are shown in grey.

For the what-if scenario where Meat prices are anchored to their projected 5th percentile values (Figure A2), the resulting Basket distribution shapes are bulk shifted to lower values, while those for the 95th percentile sensitivity test are moved higher (as might be expected, given certain other food prices are correlated with Meat price). Moreover, the standard deviations on the means are universally smaller under these Meat price sensitivity tests, and kurtosis of the distributions is reduced. In this hypothetical situation, because of the correlations structure in the Bayes Net, uncertainty distributions relating to the selected Baskets' percentage changes and monetary costs may be more Gaussian if future Meat prices were better constrained. However, the opposite may apply with other foods or combinations of foods; only detailed sensitivity testing will reveal how the estimates might change under different circumstances.

Gaining new or better information about individual contributory food price trajectories can radically change inferences about target item forecast distributions; focussing only on mean values can conceal or distract from meaningful effects, especially in patterns of tail behaviour. And, when we are dealing with multiple variables – here just ten food categories – the interplay between factors and their uncertainties will be almost impossible to evaluate reliably, without the assistance of suitable numerical software. There are always likely to be subtle, sometimes

counter-intuitive, relationships lurking in the complexities of any stochastic model of this nature.

The changes in the CPI Basket sub-food costs, expressed as percentages, appear numerically smaller than the corresponding changes if monetary costs are used. This is likely due to the weights that are applied to the different ways the various Baskets are composed. For instance, under the scenario of No deal and Meat price being set to its 95th percentile, the increase in mean percentage change for the CPI Basket is less than a factor 2x (+44% -v- +24%), whereas the monetary cost increase is more than a factor 3x greater (£44.84 -v- £13.97). Similar differences in sensitivity are present at the distribution 95th percentile levels.

Basic dependence structuring is added into the net to reflect the likely main correlations in prices that exist in reality. The converse, and frequently adopted, assumption -- that probabilistic model parameters are independent when in fact they are correlated -- introduces the risk of under-estimating the extent of joint distribution tails. At this stage, we do not claim that the correlation structures included in our Bayes Net model are comprehensive or numerically precise -- formally determining these dependencies requires further work. This said, the influence links in our net (i.e. arrows carrying correlation values) serve to offset, to some extent, the fallacious assumption that all the food price factors can be regarded as independent of one another.

To map the entire landscape of various possible Brexit-related food price projections, a full range of similar what-if sensitivity tests would be needed, including compound pricing evaluations for multiple combinations of foodstuffs, and for all, jointly; such an exercise lies outside the scope of the present analysis.

For peer review only

Anticipated impacts of Brexit scenarios on UK food prices and implications for policies on poverty and health: a structured expert judgement approach

Journal:	*BMJ Open*
Manuscript ID	bmjopen-2019-032376.R1
Article Type:	Original research
Date Submitted by the Author:	17-Dec-2019

Complete List of Authors:	Barons, Martine; University of Warwick<br>Aspinall, Willy; University of Bristol

**Primary Subject Heading**:	Public health
Secondary Subject Heading:	Health policy
Keywords:	Food poverty, PUBLIC HEALTH, chronic disease, Malnutrition, Child poverty, Health policy < HEALTH SERVICES ADMINISTRATION & MANAGEMENT

SCHOLARONE™ Manuscripts

For peer review only - http://bmjopen.bmj.com/site/about/guidelines.xhtml

I, the Submitting Author has the right to grant and does grant on behalf of all authors of the Work (as defined in the below author licence), an exclusive licence and/or a non-exclusive licence for contributions from authors who are: i) UK Crown employees; ii) where BMJ has agreed a CC-BY licence shall apply, and/or iii) in accordance with the terms applicable for US Federal Government officers or employees acting as part of their official duties; on a worldwide, perpetual, irrevocable, royalty-free basis to BMJ Publishing Group Ltd ("BMJ") its licensees and where the relevant Journal is co-owned by BMJ to the co-owners of the Journal, to publish the Work in this journal and any other BMJ products and to exploit all rights, as set out in our licence.

The Submitting Author accepts and understands that any supply made under these terms is made by BMJ to the Submitting Author unless you are acting as an employee on behalf of your employer or a postgraduate student of an affiliated institution which is paying any applicable article publishing charge ("APC") for Open Access articles. Where the Submitting Author wishes to make the Work available on an Open Access basis (and intends to pay the relevant APC), the terms of reuse of such Open Access shall be governed by a Creative Commons licence – details of these licences and which Creative Commons licence will apply to this Work are set out in our licence referred to above.

Other than as permitted in any relevant BMJ Author's Self Archiving Policies, I confirm this Work has not been accepted for publication elsewhere, is not being considered for publication elsewhere and does not duplicate material already published. I confirm all authors consent to publication of this Work and authorise the granting of this licence.

For peer review only

Anticipated impacts of Brexit scenarios on UK food prices and implications for policies on poverty and health: a structured expert judgement approach

Martine J Barons[1] [0000-0003-1483-2943]• Willy Aspinall[2] [Orcid 0000-0001-6014-6042]
[1]Director of the Applied Statistics & Risk Unit Applied Statistics & Risk Unit, Department of Statistics, University of Warwick, Coventry, UK. Martine.Barons@warwick.ac.uk Corresponding author.
[2] Principal Consultant, Aspinall and Associates, Tisbury, and University of Bristol, Bristol, UK
Willy.Aspinall@bristol.ac.uk

Abstract

Introduction Food insecurity is associated with increased risk for several health conditions and with poor chronic disease management. Key determinants for household food insecurity are income and food costs. Whereas short-term household incomes are likely to remain static, increased food prices would be a significant driver of food insecurity.

Objectives To investigate food price drivers for household food security and its health consequences in the United Kingdom under scenarios of Deal and No deal for Britain's exit from the European Union. To estimate the 5% and 95% quantiles of the projected price distributions.

Design Structured expert judgement elicitation, a well-established method for quantifying uncertainty, using experts. In July 2018, each expert estimated the median, 5% and 95% quantiles of changes in price for ten food categories under Brexit Deal and No-deal to June 2020 assuming Brexit had taken place on 29th March 2019. These were aggregated based on the accuracy and informativeness of the experts on calibration questions.

Participants Ten specialists with expertise in food procurement, retail, agriculture, economics, statistics and household food security.

Results when combined in proportions used to calculate Consumer Prices Index food basket costs, median food price change for Brexit with a Deal is expected to be +6.1% [90% credible interval:-3%, +17%] and with No deal +22.5% [+1%, +52%]

Conclusions

The number of households experiencing food insecurity and its severity are likely to increase because of expected sizeable increases in median food prices after Brexit. Higher increases are more likely than lower rises and towards the upper limits, these would entail severe impacts. Research showing a low food budget leads to increasingly poor diet suggests that demand for health services in both the short and longer term is likely to increase due to the effects of food insecurity on the incidence and management of diet-sensitive conditions.

Keywords Brexit • Food prices • Consumer Price Index • Structured expert judgement • Uncertainty

For peer review only - http://bmjopen.bmj.com/site/about/guidelines.xhtml

Strengths limitations of this study

- First study to quantify anticipated food price changes and associated uncertainty relating to Brexit Deal and No deal scenarios using a transparent and established protocol, and to articulate links to potential healthcare impacts.
- Inclusion of experts with broad and overlapping areas of expertise in food production and supply.
- Scenario analysis showing how price changes in linked food categories could combine to affect overall food costs.
- Fewer than optimal number of experts elicited.
- Study was undertaken on the assumption that Brexit would occur on 29th March 2019. Delays and major ambiguities with respect to eventual Brexit circumstances have emerged since experts made projections in 2018.

INTRODUCTION

Food insecurity, the lack of access to sufficient nutritious food, is associated with multiple negative outcomes including diet-sensitive chronic diseases. An important driver of household food security is the costs of food and other essentials relative to incomes. In 2016, the United Kingdom (UK) voted to relinquish its membership of the European Union (EU), known colloquially as 'Brexit', to be completed by 29 March 2019. UK reliance on food imports, including from EU, is significant and food price rises have been widely forecast (1).

One of the motivations for investigating possible impacts of Brexit on food prices is that, over the last few years, medical General Practitioners in the UK have raised concerns about food insecure patients seeking referrals to food banks (2) and that food insecurity is affecting medication compliance, health and wellbeing (3). This has raised concern about resource implications for surgeries (4) and that "the welfare system is failing to provide a robust last line of defence against hunger"(5).

A 2017 survey showed 13% of people were worried that their food would run out before they got money to buy more ('marginally food secure households') and 8% couldn't afford to eat balanced meals or went hungry ('low or very low food secure households')(6) . In low income households, 29% experience food insecurity(7). Lower-income households inevitably allocate a higher proportion of spending on food, and buy a similar fraction of imported food; therefore, low-income households are more exposed to food price rises. In November 2018 the United Nations OHCHR special rapporteur issued a statement in which he argued that the rising use food banks in the UK is a consequence of poverty, including in-work poverty. He recommended that the UK government should begin to measure and monitor food security (8).

Absolute income levels and volatility are both important drivers of household food insecurity (9, 10). There was little growth in real earnings in 2017–18, and the Office for Budget Responsibility forecast slow earnings growth for the following four years(11). This means that the main driver of household food insecurity will be food price. UK price inflation is measured by changes in the UK Consumer Prices Index (CPI). The CPI is based on a 'shopping basket' of goods and services, including a food element. In November 2018, the CPI inflation was 2.3% p.a. over all items and 0.5% p.a. for the food element (12). The CPI is based on actual

For peer review only - http://bmjopen.bmj.com/site/about/guidelines.xhtml

consumer spending and the food element incorporates both spending on healthy nutrition and on less healthy options. CPI alone cannot indicate if consumers are shifting towards less healthy diets because confounding effects are smoothed out across income ranges.

Consequences of food insecurity on diet-sensitive chronic diseases, including hypertension, hyperlipidaemia, and diabetes(13-19), is significant. Other effects include poor educational attainment, poor mental health and social isolation, which increases mortality(20). People on lower incomes report shopping for cheaper foods and eating less (6), eating more high fat, salty, sugar-sweetened foods, and processed meat (21). Fruit and vegetable consumption is lower in low income households (22). The medical importance of a basic nutritional safety net has long been recognised by policymakers through the Welfare Food Scheme and Healthy Start programmes. A recent systematic review identifies poor diet quality as an important preventable risk factor for non-communicable disease, responsible for one-in-five deaths globally and 127 deaths per 100,000 Britons(23). Under post-2008 austerity measures, cuts have been made to Healthy Start and other provisions. The UK's main response to growing food insecurity has been charitable food relief, but the efficacy of these and similar approaches is unmeasured. Social protection spending and welfare state interventions are the only actions known to alter the prevalence of household food insecurity(24). The rise in food bank use is attributed largely to welfare cuts(4). Despite the purported end of austerity, the inability of some households to feed themselves adequately persists. Figures from the Trussell Trust, the UK's largest network of foodbanks, show 658,048 three-day emergency food supplies were issued in the 6 months to September 2018, an increase of 13% over the same period in 2017 (25).

The UK is deeply integrated with the EU and its decision to exit from this trading block has no parallels in modern history(26). Almost one-half of the UK's food is imported: 30% comes from the EU, and another 11% comes from non-EU countries under the terms of trade deals negotiated by the EU. Prices of fruit and vegetables are particularly vulnerable to vagaries of production and supply(1). In estimates of the economic impact of Brexit on the UK, the least damaging scenarios are those which are closest to the current situation under EU membership (i.e. retaining membership of the Single Market and Customs Union), while a 'no-deal' scenario is predicted to be the most damaging(27).
In setting a comprehensive strategy for the UK to ensure household food security, policymakers must grapple with how to prioritise low food prices, animal welfare, minimum income, health, welfare and social protection.

In anticipation of Brexit in March 2019, we conducted an elicitation of a group of experts in July 2018 on their expectations for possible impacts of two Brexit scenarios on food prices over the 14 months following Brexit (i.e. to June 2020). This period was chosen to recognise that there is a period of transition to any new regime and that what we are interested in is how post Brexit food prices will settle to after the initial volatility. Experts were asked to integrate into their judgements all factors relevant to the changing of food prices at current exchange rates.

METHODS

For peer review only - http://bmjopen.bmj.com/site/about/guidelines.xhtml

A projection of food prices gives a key insight into the effects of unprecedented events on household food security and contingent health consequences. As far as we know, no projections have been published for the impact of Brexit on food prices or CPI which enumerate associated uncertainties formally. That said, one new study has estimated potential impacts of Brexit on the prices of fruits and vegetables and the uncertainties in these using Monte Carlo simulation (28). Other studies provided only point estimates. We report key findings from the application of a structured expert judgement (SEJ) elicitation to potential food price changes and their uncertainties in the event of Brexit under two scenarios: 'Deal' and 'No-deal'. By Brexit deal we mean with trading arrangements broadly similar to the present. With Brexit no-deal we mean that such arrangements will be discontinued and individual trade deals would need to be negotiated.

Since SEJ involves the combination of expert judgement, diversity of experts is more important than large numbers. Literature supports 8–15 experts as a viable number in practice; having greater numbers may not significantly impact the findings and would incur extra expense and time. Having fewer than five experts reduces the prospect of providing adequate diversity of views and could weaken the strength of the inferences(29). We identified potential experts through literature search and scanning webpages of relevant organisations. We sent invitations to 43 individuals whose expertise represented a wide range within the domain. Of these, only six were able to spend three days at the elicitation workshop, so we rescheduled and sent out another tranche of 67 invitations. Again only six could commit to the workshop, so we rescheduled once more, expanded our list of potential experts and issued 81 invitations. Following this iteration, we decided to go ahead with the six external experts and supplement the panel with additional academic colleagues. Two external experts were then late withdrawals from the panel, so added two more academic volunteers with relevant expertise bring the panel up to ten specialists in all. Our panel (see Acknowledgments) had expertise in food procurement, retail, agriculture, economics, statistics and household food security, and each expert considered potential impacts of the Brexit scenarios on prices separately for each of ten food categories that are used in the UK Consumer Prices Index (CPI). We then used these results to explore a number of scenarios.

Structured expert judgement

Structured expert judgement (SEJ) elicitation provides a formal approach for estimating uncertain quantities according to current professional knowledge and understanding, and has been utilised in a wide range of applications. To amalgamate our experts' judgements, we used Cooke's Classical Model, a well-established, validated approach (30-33). Cooke's method uses a mathematical scoring rule basis to evaluate empirical performance-based weights for aggregating individual experts' judgements. This means that when all the individual estimates are combined, the experts who were most informative and most accurate on a set of related calibration questions (to which only the analysts had the answers) contributed most to the final estimates of the questions of interest (34).

The independent facilitator (WA) devised calibration questions based on historic food price changes. The questions of interest were future food price changes. We began by discussing with the group the wordings and meanings of the questions, to minimise ambiguities or misunderstandings. The experts themselves then clarified and revised several of the target questions.

For peer review only - http://bmjopen.bmj.com/site/about/guidelines.xhtml

For both calibration questions and target questions, we asked the experts to integrate, within their judgements, all the factors and circumstances under which food prices could be driven up or down. Each expert provided their own estimates, confidentially to the facilitator (WA), for the lowest plausible, highest plausible and best estimate for price change for each food category under the specific scenario. We discussed with the experts that we would treat their three judgement values for each item in the subsequent analysis as analogous to a 90% credible interval range, with their best estimate value representing the median of their uncertainty spread. We made clear that a median value need not necessarily be central to the credible interval if, in the expert's judgement, the relevant uncertainty distribution is not symmetric and should exhibit skewness (either to higher or lower values). More details are given in supplementary information – File S1.

This confidential elicitation procedure encourages participants to express judgements based on their true beliefs, reducing potential sources of bias due to peer- and group influences. We also maintained anonymity when presenting the individual judgements and weights derived from the calibration questions.

Analysis of Elicitation

We aggregated mathematically individual uncertainty distributions for each food category to construct food price change probability density functions, using the performance-based weights derived from the expert calibration step in the Cooke's Classical Model (30).

We processed our experts' responses using the program EXCALIBUR (35, 36), which computes weighted combinations of judgements and produces a synthesis for each food item expressed in terms of the same three quantiles used in the elicitation (i.e. 5th; 50th and 95th percentiles). We report in the next section both the overall food price change using the category weights employed by the CPI and those for a healthy basket based on McMahon and Weld (2015)(37).

Computing Brexit-related Food Basket Cost Changes

In order to estimate price change distributions for different shopping baskets under the two Brexit scenarios we use the Bayes Net (BN) code UNINET (38). BNs are graphical tools for representing and computing high dimensional joint uncertainty distributions (39, 40). Our BN for calculating food basket price changes post-Brexit is shown in Figure 1.

In post-processing the elicitation data it was recognised that implicit correlations existed between certain foodstuff prices in the judgments of many of the experts. Ideally, such correlations need to be accounted for in an uncertainty analysis to avoid creating spurious results; here, to mitigate their absence, we adopted approximate correlation values from our knowledge of foodstuff pricing.
The converse assumption, that food prices are independent, introduces the risk of under-estimating the joint extent of interrelated changes on distribution tails.

Vegetable and fruit prices are correlated by a mutual dependency on weather conditions. However, both within and between food categories there can be differential effects on different crops: for example, spring floods which rot potatoes and delay grain sowing do no harm to

For peer review only - http://bmjopen.bmj.com/site/about/guidelines.xhtml

orchards, which suffer if there is an unseasonal frost as the fruit is setting. We set this correlation at about +0.75

Corn and sugar beet are used as feed for cattle, inducing a correlation between meat and grain (bread), and between meat and sugar; we set these correlations each at +0.4. While dairy and beef cattle markets are quite distinct, their prices are linked through a common dependency on feed costs, so we link milk and meat with a correlation +0.4.

Grain (bread) and sugar (beet) can both be feedstock for biofuel and thus are linked via oil price; to account for this and other, secondary joint correlations, we set this correlation to +0.72. In fact, all food prices are linked to oil price through production and transport costs, but these two more strongly.

The 2016 ‘sugar tax’ saw food producers make a substantial switch to artificial sweeteners, smaller serving sizes etc., to avoid significant price rises being passed to the consumer. In light of this, we set the correlation between sugar and soft drinks to a relatively low value of +0.3.

[FIGURE 1 HERE]

RESULTS

We present estimated projected price changes by June 2020, assuming Brexit on 29th March 2019, using food price change quantiles elicited at food category level (Table 1). We use the BN in Figure 1 to combine these price changes and associated uncertainties for the food element of the CPI basket, and to estimate monetised equivalents for specific family types based on McMahon and Weld (2015). We discuss the likely effects on health and the demand for health services. The results of the analysis are in Table 1 and are shown graphically in Figure A1. Negative values indicate that the experts judge that, under a given scenario, some prices could conceivably go down, as well as up, albeit with low probabilities.

	**Food category percentage price changes by June 2020 median (5th, 95th percentile)**	
**CPI category**	**Brexit deal**	**Brexit no-deal**
Soft drinks etc.	6 (0, 26)	8 (0, 47)
Coffee, tea & cocoa	2 (-9, 19)	4 (-5, 69)
Sugar, jam, etc.	7 (-9, 20)	19 (-5, 82)
Vegetables	3 (-10, 20)	9 (-18, 63)
Fruit	5 (-10, 24)	16 (-8, 51)
Oil & fats	5 (-9, 20)	18 (-8, 87)
Milk, cheese & eggs	6 (-9, 20)	23 (-5, 82)
Fish	4 (-9, 19)	5 (-13, 41)
Meat	6 (-10, 29)	18 (-11, 80)
Bread & Cereals	4 (-9, 19)	10 (-7, 83)

For peer review only - http://bmjopen.bmj.com/site/about/guidelines.xhtml

**Overall % change ONS CPI sub-Foods, with category weights**	**Mean +6.4% ± 6.0 Median +6.1% [-2.7, +16.9]**	**Mean +24.0% ± 15.4 Median +22.5% [+1.49, +51.7]**
	**Food basket cost changes by June 2020 (in £'s)**	
**Change in CPI weekly cost relative to 2018 year end Basket total £58.00***	**Mean +£3.78 ± £3.76 Median +£3.53 [-£1.90, +£10.41]**	**Mean +£13.97 ± £9.52 Median +£13.00 [+£0.08, +£31.02]**
**Change in family of 4 Healthy Food Basket basis weekly cost £93.56****	**Mean +£6.30 ± £6.71 Median +£5.80 [-£3.68, +£18.17]**	**Mean +£22.58 ± £16.14 Median+£20.98 [-£1.07, +£50.98]**
**Change in single pensioner Healthy Food Basket basis weekly cost £35.44****	**Mean +£2.28 ± £2.56 Median +£2.09 [-£1.51, +£6.80]**	**Mean +£8.11 ± £6.23 Median +£7.55 [-£1.05, +£18.99]**

*Table 1 Aggregated food prices change estimates. "Brexit deal" means a deal similar to the present arrangements will be implemented, so little disruption or additional costs to supply routes. "Brexit no-deal" means that such arrangements will be discontinued and individual trade deals would need to be negotiated. Numerical values are medians (90% credible intervals). *Based on ONS Table A2 2018 year end data (Mar 2018): selected Basket sub-Food category weekly costs; total for the ten items = £58.00. **Based on MacMahon and Weld (2015) Northern Ireland minimum essential Healthy Basket sub-Food category weekly costs at November 2014 Tesco prices. For two adults and two children, one in pre-school (aged 2-4) and one in primary school (aged 6-11), total cost for the ten items = £93.56; for a single pensioner, the corresponding selected items cost = £35.44.*

Using the ONS food category weightings, we aggregate their contributions to a CPI basket. We see that, under a Brexit deal, this gives expected median food price rises around +6% (i.e. a rise 12 times higher than in 2018) by June 2020, with a plausible, i.e. 1-in-20 (5%) likelihood of a drop in prices of about -3% or more and a 1-in-20 chance of a rise of +17% or more. Under Brexit no-deal, the overall median food price escalation is expected to be +23%, with a lower plausible increase of about +1% and upper plausible increase of about +52% (again, each bound has a 1-in-20 chance of being exceeded).

Thus, the foreseeable most likely outcome of Brexit is significant price rises. This will lead to more household food insecurity and its attendant deterioration in health and increase in demand for health services.

What-if scenario sensitivity testing

We wished to investigate how sensitive the overall basket food cost results are to a single food type. Currently the only UK differentials from global prices are beef and poultry, where the EU's production standards are higher than the rest of the world (41). This suggests that constraining price rises for these foods following Brexit might be achieved principally by lowering animal welfare and food hygiene standards, with associated risks to human health.

We set cost of meat to its 5th percentile level and then its 95th percentile level and investigated the effects on the overall food basket cost, with other food types unchanged. The resulting changes to the CPI Food Basket and to the Family Basket cost projections reported in Table 2 and Figures A2 and A3.

For peer review only - http://bmjopen.bmj.com/site/about/guidelines.xhtml

Meat price impacts on food basket mean costs: Scenario-based Bayes Net analytical conditioning				
Scenario	CPI Basket sub-foods cost: mean percentage change*	Family basket: mean cost change*	CPI Basket sub-foods cost: 95%ile percentage change*	Family basket: 95%ile cost change*
Meat price → projected 5th percentile cost				
**Deal** [Meat cost -10%]	-0.1% (+6.4%)	-£1.26 (+£3.78)	+6.2% (+16.9%)	+£5.01 (+£10.41)
**No deal** [Meat cost -11%]	+8.6% (+24.0%)	+£5.71 (+£13.97)	+24.4% (+51.7%)	+£21.13 (+£31.02)
Meat price → projected 95th percentile cost				
**Deal** [Meat cost +29%]	+13.5% (+6.4%)	+£14.66 (+£3.78)	+20.6% (+16.9%}	+£21.61 (+£10.41)
**No deal** [Meat cost +80%]	+44.0% (+24.0%)	+£44.84 (+£13.97)	+62.2% (+51.7%)	+£61.56 (+£31.02)

Table 2 Example impacts on CPI and Family Food Basket costs from analytical conditioning the Bayes Net to the 5th percentile and 95th percentile projected costs of Meat, under Brexit Deal and No deal scenarios.
(Corresponding base model results are shown in brackets.)*

With meat cost at 5th percentile levels in the elicitation distributions (-10% and -11%, deal & no-deal respectively, see Table 1), the CPI Basket mean change for Brexit Deal becomes -0.1% (cf +6.4%) and the Family Basket mean cost changes from +£3.78 to -£1.26. Under the Brexit No-deal scenario the CPI Basket mean change would be +8.6% (cf +24.0%) and, for the Family Basket, the cost increase becomes +£5.71 (c.f. +£13.97).

With meat cost at 95th percentile levels from the elicitation Deal and No-deal distributions (+29% and +80%, respectively) the CPI Basket mean change for Brexit deal increases from +6.4% to + 13.5% and the Family Basket mean cost rises from +£3.78 to +£14.66. Under the Brexit No-deal scenario the Family Basket, the mean change would be +44.0% (cf+24.0%) the cost increase becomes +£44.84 (c.f. +£13.97).

The mean (expected) CPI Basket and Family Basket cost changes would be kept close to zero only if Meat prices were, somehow, limited to near their projected 5th percentile levels - and, with them, the price changes of foods correlated with Meat were also curbed - and then only under the Brexit Deal scenario. Otherwise, higher Meat prices will inevitably amplify basket cost changes. The standard deviations on the means are smaller under these Meat price sensitivity tests, and the correlation structure reduces the kurtosis of the distributions.

Although there are some notable differences in the item price compositions of the CPI food basket and the family 'healthy' food basket (e.g. lower spend in UK CPI basket on meat: £12.80 - v- £30.18 per week for Northern Ireland), our analysis shows that overall cost percentage

For peer review only - http://bmjopen.bmj.com/site/about/guidelines.xhtml

changes in these two representative household food baskets differ little: for Brexit no-deal, both estimates represent about +22% increases in projected mean costs.

We selected meat for this sensitivity analysis as it the most likely to change in price depending on the details of any trade deals agreed. For other foods or combinations of foods, the opposite may apply; similar sensitivity analyses are possible.

The key finding is that the most likely effect of Brexit on food prices as calculated using the CPI method is a median rise of 6% if there is a deal and 23% if there is no deal. These represent significant additional costs for household budgets and are highly likely to lead to poorer diets with the concomitant effects on diet-related health.

DISCUSSION

Principal findings

Food price rises after Brexit are likely to be significant and may be substantial and changes will be felt by the whole population very quickly(1). In the light of the expected stagnation of household incomes, this is likely to drive more households into food insecurity. Food is a substantial portion of household expenditure, especially in low-income households(1). MacMahon and Weld (37) found that, after housing and childcare costs, the highest category of household expenditure in Northern Ireland is on a minimum essential 'healthy' food basket. They found food costs to be more expensive in rural areas for all household types and the highest spend was on meat, followed by the category fruit and vegetables. Those households buying most would, of course, incur the greatest actual spend increases, with concomitant implications for affordability in terms of differing household-related incomes. The well-established links between lower budgets available for food and lower diet quality lead to the expectation of multiple negative outcomes. Nutrient-dense foods, such as fruits and vegetables, are often more expensive and less available in lower-income neighbourhoods when compared with processed foods. Processed foods are generally inexpensive and highly accessible. They are energy-dense, high in added fats, sugar, or salt, and often considered highly palatable with addictive potential (21). Increasing numbers of patients with this kind of diet will likely drive increases in the incidence of diet-sensitive chronic diseases in the longer term, such as hypertension, hyperlipidaemia, and diabetes (13-19). The downturn in fruit and vegetable intake has been estimated between 2.5% and 11.4%, dependent on the Brexit trade agreement, with increasing cardiovascular disease mortality (28). This in turn will drive increased demands on the health services. In the short term, this might manifest in reduced control of existing chronic conditions such as diabetes, coeliac disease, and hypertension, leading to demand on front-line and general practice services.

Strengths and weaknesses of the study

One strength of our analysis is that the expert judgement median estimates we obtain by elicitation are consistent with central estimates produced by UK Trade Policy Observatory and by the British Retail consortium(1) and other modelling studies (28). However, we add further information for decision support by presenting quantified uncertainties around our estimates. These spreads can be substantial and all exhibit skew in the form of extended, 'heavy' upper tails – i.e. larger price increases are more likely than smaller increases (or reductions). Related decision-making that is based only on central (average) estimates and neglects these uncertainties can lead to poor policy selection (42).

For peer review only - http://bmjopen.bmj.com/site/about/guidelines.xhtml

Policymakers benefit from consideration of a defined reasonable worst case for contingency planning, and are proficient at interpreting probabilistic statements, which help clarify underlying assumptions(43). Moreover, historic vegetable CPI rises offer a perspective for the elicited credible intervals for CPI vegetable price change in Table 1. The largest historic one-year change in vegetable prices since 1987 was +14.8% (2006-7) and the largest two-year jump was +27.1%, for the period 2006 to 2008. Compared to the Brexit deal scenario, the record two-year rise is greater than the elicited 95th percentile vegetable price index change (i.e. +20% by June 2020, two-years ahead from the elicitation), and falls within the 90% credible interval for Brexit No-deal;, the projected 95th percentile change could exceed +63% under this scenario. One weakness of the study was that we had fewer than the optimal number of experts. Whilst the spread of expertise was good, a wider spread of expertise could have improved quantification of price changes, possibly narrowing the uncertainty around the central estimates.

Strengths and weaknesses in relation to other studies

Although updating is warranted, our likely price change projections exemplify a basis for undertaking detailed modelling of impacts on health and healthcare provision under the two alternative Brexit scenarios. Most attempts to quantify food prices after Brexit have not been set within the context of health and healthcare provision, nor have they quantified the uncertainty in such estimates; one notable exception is the estimation of the potential impacts of fruit and vegetable price increases on cardiovascular disease (28).Whilst the experience of food insecurity is largely driven by the cost of food and other essentials relative to incomes, other factors such as self-efficacy, access to credit and other forms of social capital are also significant (44).

Meaning of the study

The likely effect of Brexit, under either scenario, is a significant rise in food prices. Unless the rising tide of food insecurity is reversed, health costs will continue to rise with implications for health and clinician workloads. Our findings should alert policymakers to the potential for significant increases in food costs under either Brexit scenario, with major impacts likely to follow a no-deal outcome. The expected levels of these increases and, more importantly, the uncertainty spreads on the estimates - all of which are skewed moderately toward higher costs - should inform policies that allow households to afford minimum essential food baskets, meeting acceptable physical, psychological and social needs. One likely corollary to substantial post Brexit food price rises is even greater consumption of cheaper, less healthy diets, with inevitable impacts on population long-term health trends and demands on the NHS. Medical practitioners and health care workers are amongst those who will have to confront the related challenges if food prices rise sharply and substantially after Brexit. Clinicians may need to organise processes to cope with the demand from food insecure patients for referrals to foodbanks. The management of chronic conditions may deteriorate, increasing the need for intervention. Food insecurity in the present may also increase healthcare demand in the longer term. Policies that support incomes and access to food are the only interventions that have a demonstrable positive effect on food insecurity. It is incumbent on policymakers to ensure that the last line of defence against hunger is robust in order to promote well-being, productivity and healthy ageing.

Current evidence on the efficacy of some of the most widespread current responses to food insecurity, such as foodbanks, is limited. More research is needed in order to select the most effective strategies for supporting health and well-being. While Brexit did not take place by the due date, and the overall political situation is now even less clear than before, certain aspects

For peer review only - http://bmjopen.bmj.com/site/about/guidelines.xhtml

of potential impacts on food supplies have become more explicit. For example, the impact of stockpiling on non-perishable food prices can be now ascertained more accurately, as those costs can be quantified. In other respects, however, uncertainties associated with post-Brexit food pricing are likely to have increased. For instance, at the time of writing (December 2019), the current target date for Brexit has moved to 31 January 2020, and might be postponed even later. Inevitably, the precise timing of Brexit will have an impact on the prices of fresh foods due to the seasonal nature of such produce.

Unanswered questions and future research

Given almost everything concerning Brexit is in a state of flux at the time of writing, it is our intention to conduct an updating elicitation, once the circumstances surrounding how Brexit will be implemented become clearer. Experts who participated in our 2018 elicitation have expressed a universal interest in re-visiting the issues and factors, and in reviewing their judgements of future food prices and the related uncertainties. We will consider increasing membership of the panel of experts to obtain an even wider sample of judgements. One advantage of this repeat elicitation would be the opportunity to investigate how much the quantified uncertainties, reported here, may have changed. When we know the terms of Brexit, an elicitation update will be able to quantify possible adjustments to the present estimates. In addition, we intend to explore in more quantitative detail possible underlying correlations between prices of different foodstuffs. New advances in elicitation techniques for assessing parameter dependences (45) offer a ratifiable basis for quantifying the properties of such correlations. It seems inevitable that the implications of an eventual Brexit for projected food price forecasts will be fundamental to, and crucial for policy planning in many societal and political areas.

Acknowledgements

We acknowledge with gratitude the invaluable contributions of the experts who participated in the elicitation workshop, 4-6 July 2018:

Martine J. Barons (University of Warwick, Department of Statistics)
Steve Brewer (University of Lincoln, Lincoln Institute for Agri-Food Technology (LIAT), Network Coordinator for the Internet of Food Things Network Plus)
Rosemary Collier (University of Warwick, Life Sciences, Crop Centre)
Peter Crosskey (freelance food journalist, formerly of The Grocer)
Jonathan Horsfield (retired from Warwickshire County Council; energy expert)
Jim Q Smith (University of Warwick, Department of Statistics)
Thijs van Rens (University of Warwick, Department of Economics)
Rachel Wilkerson (University of Warwick, Department of Statistics)
Sophia Wright (University of Warwick, Department of Statistics)

Other participating experts from industry preferred to remain anonymous.

Funding The workshop was funded by the Warwick Global Research Priority for Food. The study is part of work undertaken for EPSRC grant number EP/K007580/1.

Competing interests None declared.

Contributions MJB conceived the study and organised the workshop and experts' participations. WA advised MJB on the elicitation and acted as a neutral independent facilitator

For peer review only - http://bmjopen.bmj.com/site/about/guidelines.xhtml

for the elicitation and processed the experts' responses. MJB provided information for contextualising the elicitation findings, and both authors jointly wrote the paper.

Ethics approval; Patient and Public Involvement No patient involved.

Provenance and peer review Not commissioned; external peer reviewed.

Data sharing statement The first author may be contacted for requests for data or information used in this study.

Inclusion criteria The study did not use or rely directly on other published data.

Open access This is an Open Access article distributed in accordance with the terms of the Creative Commons Attribution (CC BY 4.0) license, which permits others to distribute, remix, adapt and build upon this work, for commercial use, provided the original work is properly cited. See: http://creativecommons.org/licenses/by/4.0/

For peer review only

REFERENCES

1. HOUSE OF LORDS European Union Committee. Brexit: food prices and availability. In: HOUSE OF LORDS European Union Committee, editor. 14th Report of Session 2017–19, ed. London, UK: HOUSE OF LORDS European Union Committee,; 2018.
2. Matthews-King A. One in six GPs asked to refer a patient to a food bank in the past year. In: Pulse, editor. Pulse: At the heart of general practice since 1960. UK: Cogora Limited, 140 London Wall, London, EC2Y 5DN, United Kingdom,; 2014.
3. Pugh R. 'It's a life or death situation': why GPs are referring patients to food banks. The Guardain. 2018 09/02/2018;Sect. Healthcare.
4. Rachel Loopstra, Aaron Reeves , David Taylor-Robinson , Ben Barr, Martin McKee, David Stuckler Austerity, sanctions, and the rise of food banks in the UK. British Medical Journal. 2015;350.
5. Ashton JR, Middleton J, Lang T. Open letter to Prime Minister David Cameron on food poverty in the UK. The Lancet. 2014;383:1631-.
6. Bates B, Roberts C, Lepps H, Porter L. The Food & You Survey Wave 4 Combined Report for England, Wales and Northern Ireland In: Agency FS, editor.: NatCen Social Research 2017.
7. Nelson M, Erens B, Bates B, Church S, Bosher T. Low income diet and nutrition survey. Vol 3. Nutritional status, physical activity, economic, social and other factors. In: Food Standards Agency, editor. 2007.
8. Alston P. Statement on Visit to the United Kingdom, by Professor Philip Alston, United Nations Special Rapporteur on extreme poverty and human rights. In: Commissioner OotH, editor.: OHCHR; 2018.
9. Leete L, Bania N. The effect of income shocks on food insufficiency. Review of Economics of the Household. 2010;8(4):505-26.
10. Morduch J, Schneider R. The Financial Diaries: How American Families Cope in a World of Uncertainty. 1 ed. Oxford, UK: Princeton University Press; 2017. 248 p.
11. Cribb J, Keiller AN, Waters T. Living standards, poverty and inequality in the UK: 2018. In: Studies TIfF, editor. 2018.
12. Office for National Statistics. Consumer price inflation tables UK: Office for National Statistics,; 2019 [updated 13 November 2019. Available from: https://www.ons.gov.uk/economy/inflationandpriceindices/datasets/consumerpriceinflation.
13. Seligman HK, Laraia BA, Kushel MB. Food Insecurity Is Associated with Chronic Disease among Low-Income NHANES Participants. The Journal of Nutrition. 2009;140(2):304-10.
14. Townsend MS, Peerson J, Love B, Achterberg C, Murphy SP. Food Insecurity Is Positively Related to Overweight in Women. The Journal of Nutrition. 2001;131(6):1738-45.
15. Vozoris NT, Tarasuk VS. Household Food Insufficiency Is Associated with Poorer Health. The Journal of Nutrition. 2003;133(1):120-6.
16. Laraia BA. Food Insecurity and Chronic Disease. Advances in Nutrition. 2013;4(2):203-12.
17. Hampton T. Food Insecurity Harms Health, Well-being of Millions in the United States. JAMA. 2007;298(16):1851-3.
18. Seligman HK, Bindman AB, Vittinghoff E, Kanaya AM, Kushel MB. Food Insecurity is Associated with Diabetes Mellitus: Results from the National Health Examination and Nutrition Examination Survey (NHANES) 1999–2002. Journal of General Internal Medicine. 2007;22(7):1018-23.
19. Lyles CR, Wolf MS, Schillinger D, Davis TC, DeWalt D, Dahlke AR, et al. Food Insecurity in Relation to Changes in Hemoglobin A1c, Self-Efficacy, and Fruit/Vegetable Intake During a Diabetes Educational Intervention. Diabetes Care 2013 36(6):1448-53.
20. Steptoe A, Shankar A, Demakakos P, Wardle J. Social isolation, loneliness, and all-cause mortality in older men and women. Proceedings of the National Academy of Sciences. 2013:201219686.
21. Leung CW, Epel ES, Ritchie LD, Crawford PB, Laraia BA. Food Insecurity Is Inversely Associated with Diet Quality of Lower-Income Adults. Journal of the Academy of Nutrition and Dietetics. 2014;114(12):1943-53.

For peer review only - http://bmjopen.bmj.com/site/about/guidelines.xhtml

22. Public Health England. Statistical Summary: National Diet and Nutrition Survey: Years 1 to 9 of the Rolling Programme (2008/09 – 2016/17): Time trend and income analyses. England, UK: Public Health England,; 2017.
23. Afshin A, Sur PJ, Fay KA, Cornaby L, Ferrara G, Salama JS, et al. Health effects of dietary risks in 195 countries, 1990–2017: a systematic analysis for the Global Burden of Disease Study 2017. The Lancet. 2019;393(10184):1958-72.
24. Loopstra R. Interventions to address household food insecurity in high-income countries. . Proceedings of the Nutrition Society,. 2018; 77(3):270-81.
25. The Trussell Trust. End of Year Stats UK: The Trussell Trust,; 2019 [Available from: https://www.trusselltrust.org/news-and-blog/latest-stats/end-year-stats/.
26. Bergin A, Economides P, Garcia-Rodriguez A, Murphy G. Ireland and Brexit: Modelling the impact of deal and no-deal scenarios. Dublin: Economic and Social Research Institute; 2019.
27. Mott H. Exploring the Economic Impact of Brexit on Women Women's Budget Group; 2018.
28. Seferidi P, Laverty AA, Pearson-Stuttard J, Bandosz P, Collins B, Guzman-Castillo M, et al. Impacts of Brexit on fruit and vegetable intake and cardiovascular disease in England: a modelling study. BMJ Open. 2019;9(1).
29. EFSA. Guidance on Expert Knowledge Elicitation in Food and Feed Safety Risk Assessment. European Food safety Authority (EFSA) Journal. 2014;12(6):3734-.
30. Cooke RM. Experts in Uncertainty - opinion and subjective probability in science. New York: Oxford University Press; 1991 321 p.
31. Aspinall W. A route to more tractable expert advice. Nature. 2010;463:294-5.
32. Sutherland WJ, Burgman M. Policy advice: Use experts wisely. Nature. 2015:317–8-–8.
33. Colson AR, Cooke, R. M. . Cross validation for the classical model of structured expert judgment. . Reliability Engineering and System Safety 2017;163:109-20.
34. Hemming V, Armstrong N, Burgman MA, Hanea AM. Improving expert forecasts in reliability: Application and evidence for structured elicitation protocols. Quality and Reliability Engineering International. 2019;1:1-19.
35. LightTwist Software. Excalibur software for structured exoert judgement elicitation using Cooke's Classical Model. In: LightTwist Software, editor. LightTwist Software,. Austrlaia2019.
36. Cooke R, Solomatine D. EXCALIBR User's Manual. Delft, The Netherlands: Delft University of Technology/SoLogic; 1992.
37. MacMahon B, Weld, G. The cost of a minimum essential food basket in the Republic of Ireland. Study for six household types. . 2015.
38. Ababei D. UNINET. Software designed by the Risk and Environmental Modeling Group, Delft University of Technology. Developed by D. Ababei, Lighttwist Software. . 2016.
39. Smith JQ. Bayesian Decision Analysis, principles and practice. Cambridge: Cambridge University Press; 2010.
40. Jensen FV, Nielsen TD. Bayesian Networks and Decision Graphs Second Edition ed. New York: Springer; 2010.
41. Farmers Weekly. Opinion: Cheap food after Brexit is an illusion. In: Weekly F, editor. Farmers Weekly. UK: Farmers Weekly; 2018.
42. Smith JQ, Barons MJ, Leonelli M. Coherent inference for integrating decision support systems. arXiv. 2017.
43. Stirling A. Keep it complex. Nature. 2010;468 1029–31.
44. Loopstra R. Household Food Insecurity in Canada: Towards an Understanding of Effective Interventions. Toronto, Canada: University of Toronto; 2014.
45. Bamber JL, Oppenheimer M, Kopp RE, Aspinall WP, Cooke RM. Ice sheet contributions to future sea-level rise from structured expert judgment. Proceedings of the National Academy of Sciences. 2019;116(23):11195-200.

For peer review only - http://bmjopen.bmj.com/site/about/guidelines.xhtml

Figure 1 Bayes Net structure for calculating distributions for food basket price changes (ellipses with black ends) due to elicited judgments on individual foodstuff price movements under Brexit Deal and No Deal scenarios: percentage change in CPI Food Basket cost; cost change in £ for CPI Food Basket, and for two household baskets. The information nodes in the upper half of the BBN (Bread; Meat .. etc) comprise uncertainty distributions on price movements per foodstuff for the Brexit Deal scenario; the nodes in the lower half (BreadX; MeatX .. etc) represent uncertainty judgments for foodstuff price movements under a Brexit No Deal scenario. The quantified changes in the basic CPI Basket(s) are factored with ONS foodstuff weights (node "Wts"). Numerical distribution statistics for the output nodes are summarised on Table 1. (See Supplementary Information - File S2 for further details).

For peer review only

For peer review only - http://bmjopen.bmj.com/site/about/guidelines.xhtml

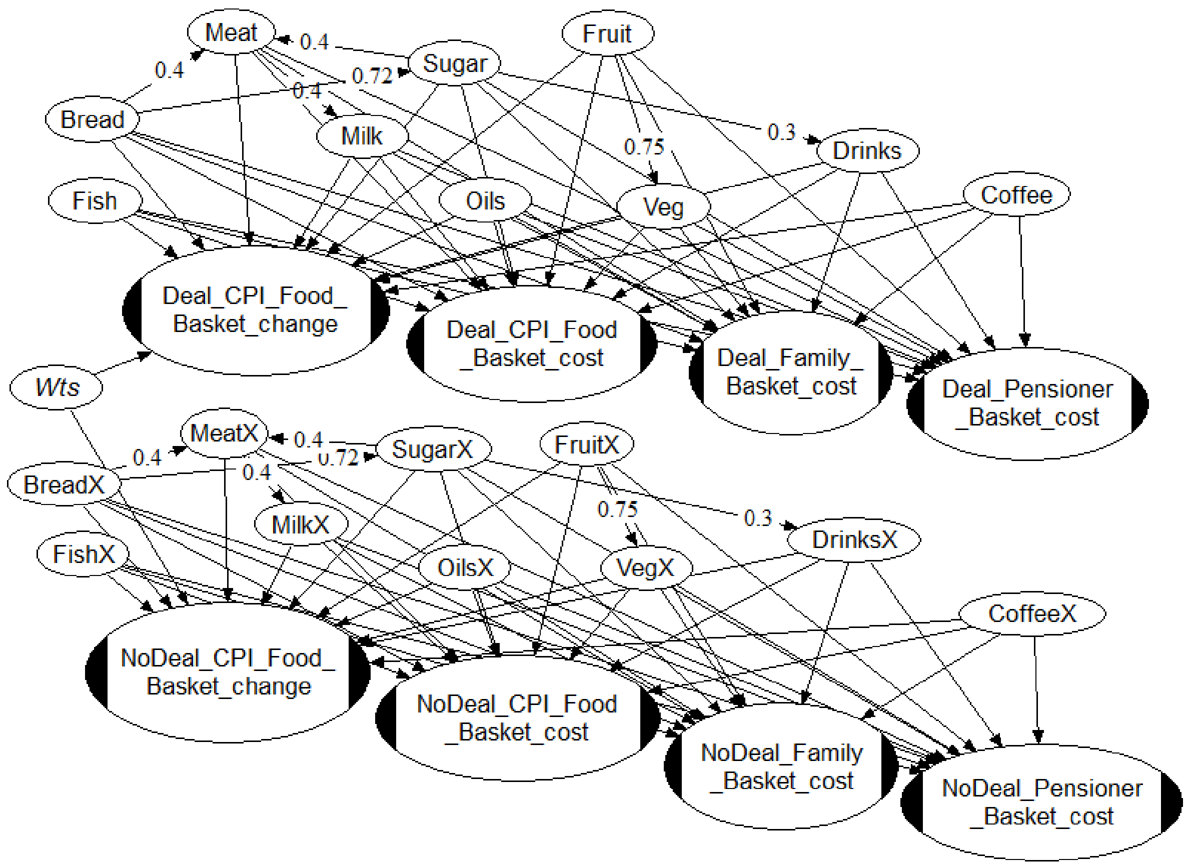
Meat
0.4
Sugar
Fruit
0.4
0.72
0.4
Bread
Milk
0.3
0.75
Drinks
Fish
Oils
Veg
Coffee
Deal_CPI_Food_
Basket_change
Deal_CPI_Food
_Basket_cost
Deal_Family_
Basket_cost
Deal_Pensioner
_Basket_cost
Wts
MeatX
0.4
SugarX
FruitX
0.4
0.72
0.4
BreadX
MilkX
0.75
0.3
DrinksX
FishX
OilsX
VegX
CoffeeX
NoDeal_CPI_Food_
Basket_change
NoDeal_CPI_Food
_Basket_cost
NoDeal_Family
_Basket_cost
NoDeal_Pensioner
_Basket_cost

review only

Supplementary Information – File S1

Barons, M. and Aspinall, W. **Anticipated impacts of Brexit scenarios on UK food prices and implications for policies on poverty and health: a structured expert judgement approach**

Summary of file contents

This file serves as a document of record for several details and aspects of a research Workshop that was convened in July 2018 to discuss and consider the potential impact of two alternative Brexit scenarios on food prices. The contents of this file relate to material that is too extensive to include in the main paper, and also cover details of the structured elicitation that was adopted to furnish the price change estimates reported in the paper. Other supporting information may be found in Supplementary File S2.

The objective of the Workshop was to develop estimates and quantify associated uncertainties in respect of several key variables that can influence future food prices, in order to provide decision support for UK policymakers concerned with food insecurity, public reliance on food banks, impacts on public health and related issues.

Contents

Supplementary Information – File S1 1
- Summary of file contents 1

Why the workshop was held 2

Structured Expert Judgement Elicitation 2
- Experts' scores 3
- Findings on target item food price changes 5

Discussion of results 18
- Target item comments 18

Appendix S1.1 Target items questionnaire 21

Appendix S1.2 Workshop Invitation 27

Appendix S1.3 Briefing note sent to participants 29

For peer review only

Why the workshop was held

This workshop was held as part of research aimed at providing decision support for policymakers concerned with ameliorating UK household food security (see also Appendix S2.2 Workshop Invitation). It is well known that critical factors in the pathway top household food insecurity are equivalised household disposable income and the cost of food.

Food prices in the UK are monitored as part of the Consumer Prices Index (CPI), the inflation measure used in the government's target for inflation. Consumer price inflation is the rate at which the prices of goods and services bought by households rise or fall. A convenient way of thinking about this is to imagine a very large "shopping basket" containing those goods and services bought by households. As the prices of the various items in the basket change over time, so does the total cost of the basket. Movements in consumer price inflation indices represent the changing cost of the shopping basket.

Although kept constant within year, the contents of the consumer price inflation basket of goods and services and their associated expenditure weights are updated annually. This is important in helping to avoid potential biases that might otherwise develop over time – for example, due to the development of entirely new goods and services, or the tendency for consumers to move away from buying goods and services that have risen relatively rapidly in price and to goods and services whose prices have fallen.

Changes to the items and their associated item weights are introduced in the February index each year, but prices are collected for both old and new items in January. This means that the figures for each year can be "chain linked" together to form a long-run price index spanning many years. In other words, price changes between December and January are based on the old basket and weights, while price changes between January and February, and beyond, are based on the new basket and weights. This procedure ensures that the annual changes to the basket and weights do not introduce a discontinuity in prices as measured by the indices.

(see: https://www.ons.gov.uk/economy/inflationandpriceindices/articles/ukconsumerpriceinflationbasketofgoodsandservices/2018)

Food and non-alcoholic beverages comprise one element of the UK CPI basket. This element is divided into 10 broad categories, with several sub-categories in each. The 10 categories are: Bread and cereals; Meat; Fish; Milk, cheese and eggs, Oils and fats; Fruit; Vegetables including potatoes and tubers; Sugar, jam, syrups, chocolate and confectionery; Coffee, tea and cocoa; Mineral waters, soft drinks and juices. In the workshop in July 2018 experts were asked to estimate the changes in prices of each of these food categories under a range of Brexit-related scenarios which were then expected to be implemented in March 2019; Brexit did not happen at that time.

Structured Expert Judgement Elicitation

When policymakers wish to make evidence-based decisions, they often take evidence from relevant experts. There are several established, tested structured elicitation protocols which aim to ameliorate biases and other frailties associated with gathering scientific advice.

In the present case, Cooke's method (Cooke, R.M. 1991 *Experts in Uncertainty*, OUP.), sometimes called the Classical Model, was adopted as the framework for eliciting and pooling experts' judgments about future food prices (a briefing note for participants is reproduced in Appendix S2.3).

For peer review only - http://bmjopen.bmj.com/site/about/guidelines.xhtml

It is a mathematical formulation for combining the views of a panel of experts, with their expressed uncertainty distributions on future food prices weighted by their performance scores from a set of calibration questions designed to assess an individual's statistical accuracy and informativeness. In the Classical Model, statistical accuracy is measured as the P-value or probability with which one would falsely reject the hypotheses that an expert's probability assessments were statistically accurate. A low value (near zero) means it is very unlikely that the discrepancy between an expert's probability statements and observed outcomes should arise by chance. Informativeness is measured as Shannon relative information with respect to an analyst-supplied background measure. Shannon relative information is used because it is scale invariant, tail insensitive, slow, and familiar.

Solutions to the elicitation of target item food price changes are presented as Performance Weighted (PW) combinations of the panel members' judgments and, for context, these PW solutions are usually compared with Equal Weights (EW) combinations, when there is no differential weighting of experts.

There are two flavours of performance weights: (1) *global weights*, which compute an expert's *combined score* as the product of his/her P-value (i.e. statistical accuracy score on the seeds) and the average of the expert's global information score over all seed and target variables; (2) *item weights*, which are computed specific to each variable/item, being the product, item-by-item, of the P-value (which is always global over all seeds) and the expert's information score for each separate variable/item.

Item weighting allows an expert, in effect, to up/down weight him/herself per item if (s)he thinks (s)he knows more/less about that item. If experts perform this up/down weighting well, item weights will out-perform global weights which, by definition, are more generic/non-specific. Hence, in the absence of contra-indications, we prefer *item weights* solutions to *global weights* as they offer more informative solutions, per item.

Experts' scores

In the present elicitation, ten participants completed the calibration exercise, and their statistical accuracy and information scores are shown on the following table.

Table S2. 1 Panel experts' scores from Classical Model calibration (see text)

Expert	Statistical accuracy (P-value)	Information score
1	0.0750	1.24
2	0.0171	1.25
3	0.0063	1.79
4	0.0028	0.97
5	0.0003	1.82
6	5.6E-05	1.40
7	1.9E-06	1.98
8	1.9E-06	1.97
9	2.8E-09	1.82
10	1.3E-10	2.24

The experts are ordered, anonymously, in terms of their P-values scores; expert labels, 1, 2 … 10 are not related to individual identities, nor do these identifiers correspond to labels used elsewhere in

For peer review only - http://bmjopen.bmj.com/site/about/guidelines.xhtml

this document. Three of the ten experts achieved scores indicating that they provided informative and statistically accurate judgments over the set of calibration questions.

The corresponding scores are shown in Figure S2.1:

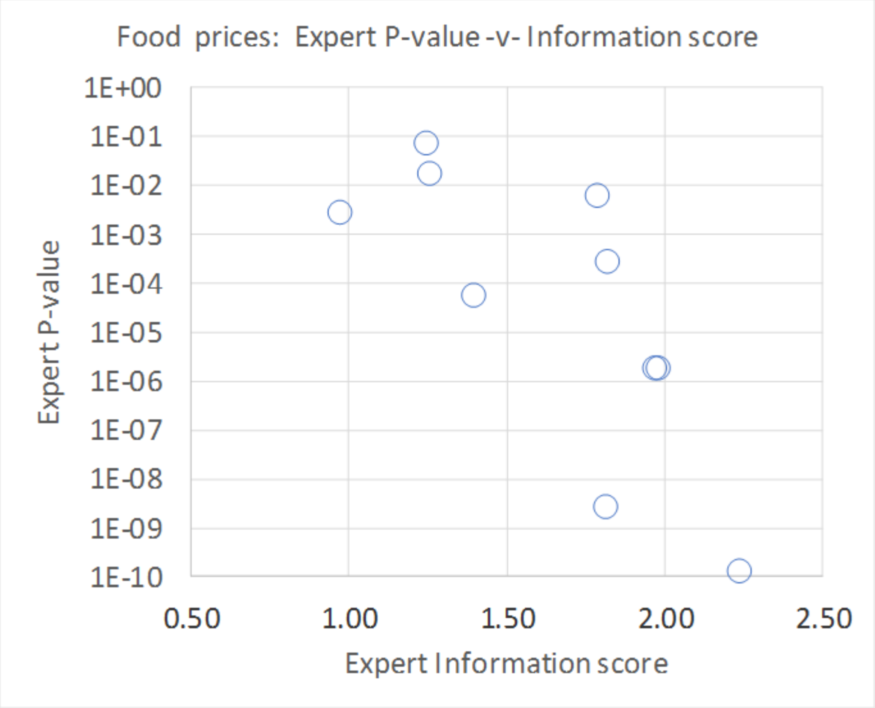

Figure S2. 1 Relationships of experts' statistical accuracy (P-value) scores to their Information scores (see text for explanation)

This plot shows that the food prices group evinced the usual trait of generally individual low P-values due to unduly high Information scores; this profile is encountered with nearly every professional expert group elicitation conducted with the Classical Model.

In short, there is a negative correlation between individuals' information scores (on seed items) and their corresponding P-values (statistical accuracy). Thus, several of the group recorded low P-values mainly because their uncertainty spreads were systematically too narrow; i.e. many demonstrated a tendency to express judgments that result in a high Information score but, because of this, they failed to capture enough realization values in the set to gain a high statistical accuracy score overall.

With the Classical Model providing a formal mathematical basis for expert scoring, this sort of expert group profile is unexceptional in structured elicitations that focus on uncertainty quantification for parameters and variables that are difficult to constrain otherwise, by statistical analysis, modelling or from theoretical considerations.

For peer review only - http://bmjopen.bmj.com/site/about/guidelines.xhtml

Findings on target item food price changes

The following range graphs record the median and 90% credible interval spreads provided by individual experts for each target question, together with the resulting Equal Weights ('EqWts') and Performance Weights ('PerfWts') solutions. The questions that were set before the participants for their judgments are reproduced in Appendix S1.1.

Subject matter: Changes in food prices with and without an agreed BREXIT trade deal (NB NO BREXIT in the plot title means no deal = hard BREXIT; agreed BREXIT means tade deals have been negotiated)

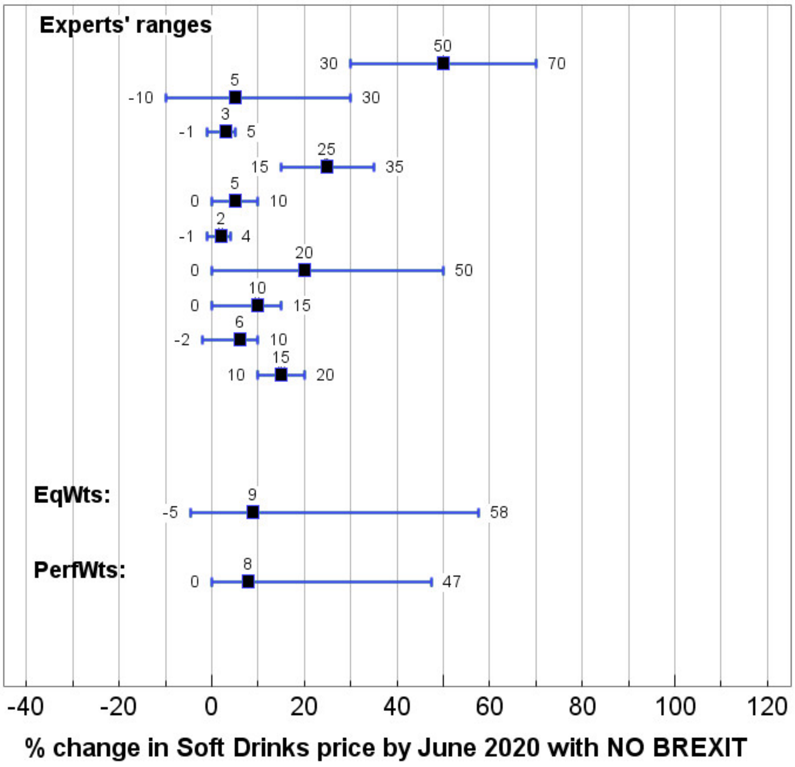

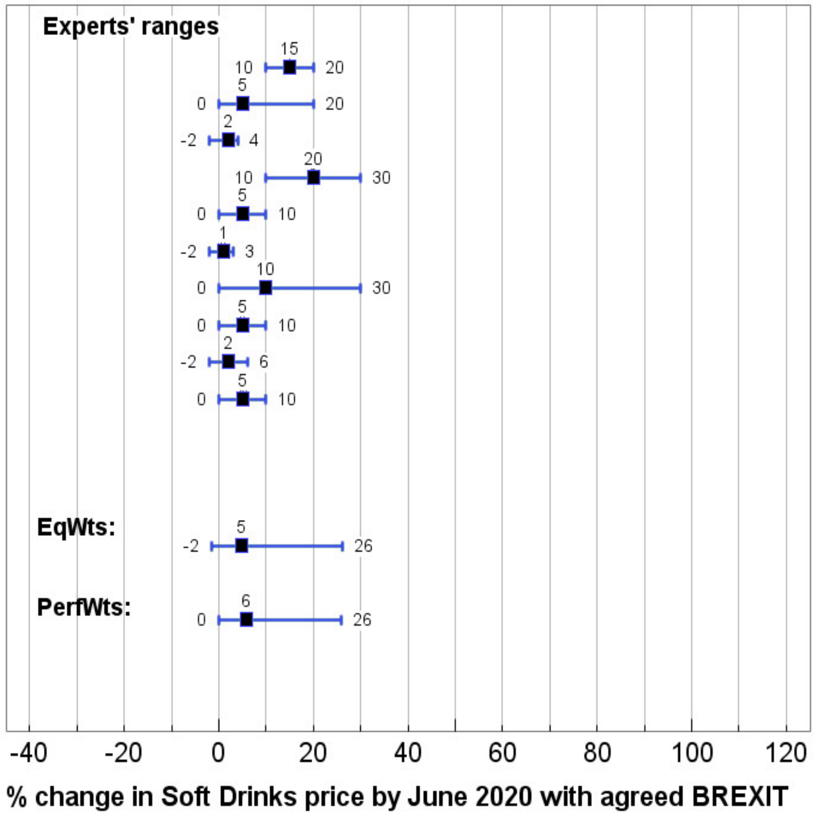
Experts' ranges
15
10 20
5
0 20
2
-2 4
20
10 30
5
0 10
1
-2 3
10
0 30
5
0 10
2
-2 6
5
0 10
EqWts:
5
-2 26
PerfWts:
6
0 26
-40 -20 0 20 40 60 80 100 120
% change in Soft Drinks price by June 2020 with agreed BREXIT

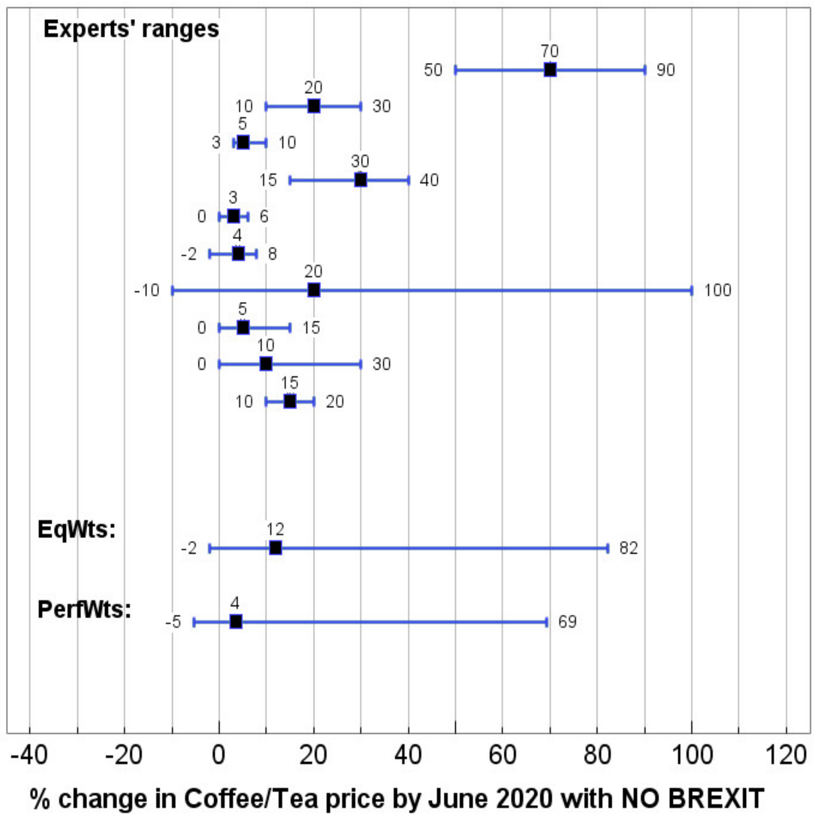
Experts' ranges
70
50
90
20
10
30
5
3
10
30
15
40
3
0
6
4
-2
8
20
-10
100
5
0
15
10
0
30
15
10
20
EqWts:
12
-2
82
PerfWts:
4
-5
69
-40 -20 0 20 40 60 80 100 120
% change in Coffee/Tea price by June 2020 with NO BREXIT

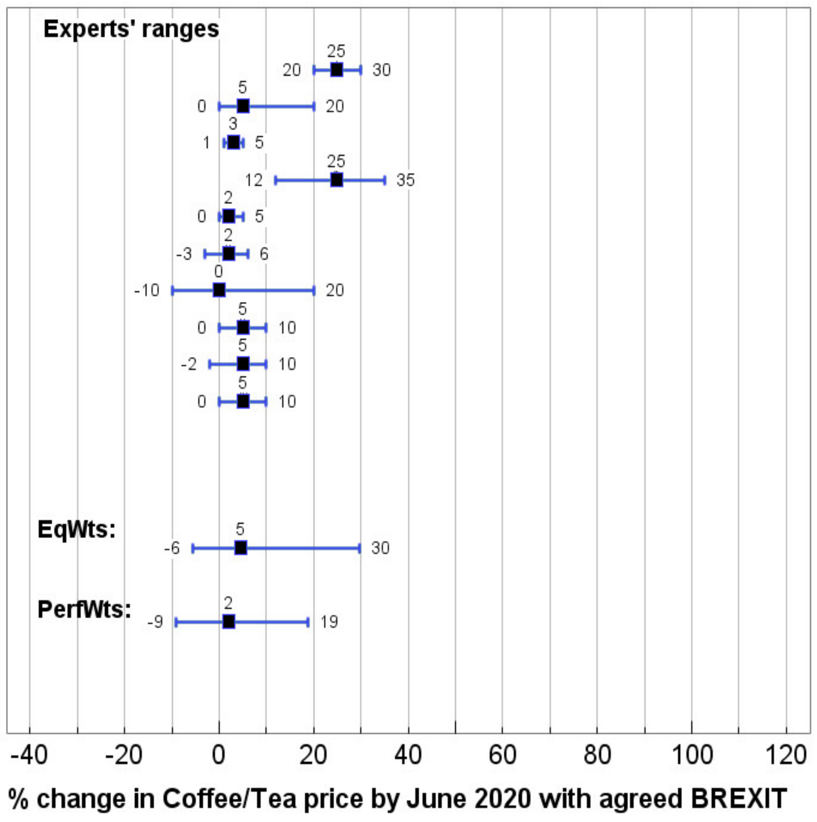
Experts' ranges
25
20
30
5
0
20
3
1
5
25
12
35
2
0
5
2
-3
6
0
-10
20
5
0
10
5
-2
10
5
0
10
EqWts:
5
-6
30
PerfWts:
2
-9
19
-40 -20 0 20 40 60 80 100 120
% change in Coffee/Tea price by June 2020 with agreed BREXIT

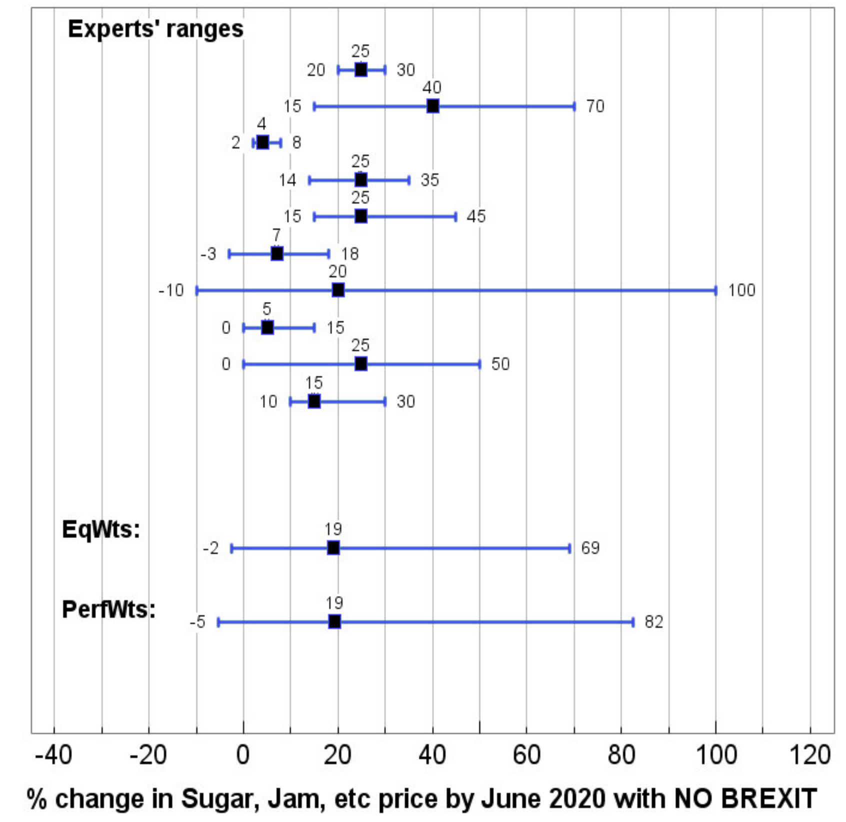
Experts' ranges
25
20 30
40
15 70
4
2 8
25
14 35
25
15 45
7
-3 18
20
-10 100
5
0 15
25
0 50
15
10 30
EqWts:
19
-2 69
PerfWts:
19
-5 82
-40 -20 0 20 40 60 80 100 120
% change in Sugar, Jam, etc price by June 2020 with NO BREXIT

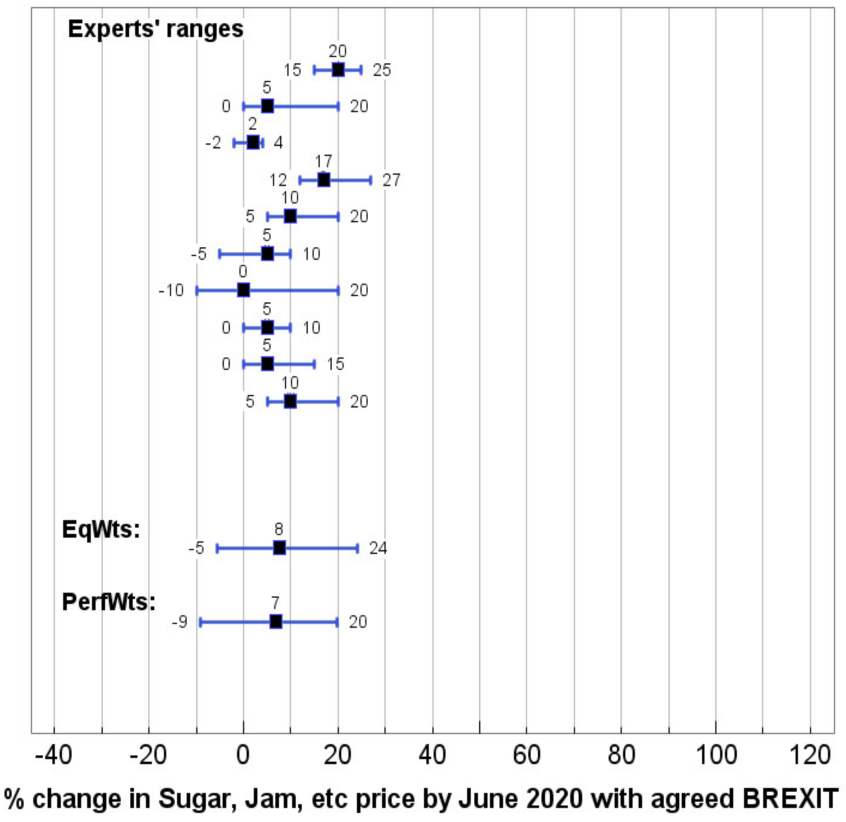
Experts' ranges
20
15 25
5
0 20
2
-2 4
17
12 27
10
5 20
5
-5 10
0
-10 20
5
0 10
5
0 15
10
5 20
EqWts:
8
-5 24
PerfWts:
7
-9 20
-40 -20 0 20 40 60 80 100 120
% change in Sugar, Jam, etc price by June 2020 with agreed BREXIT

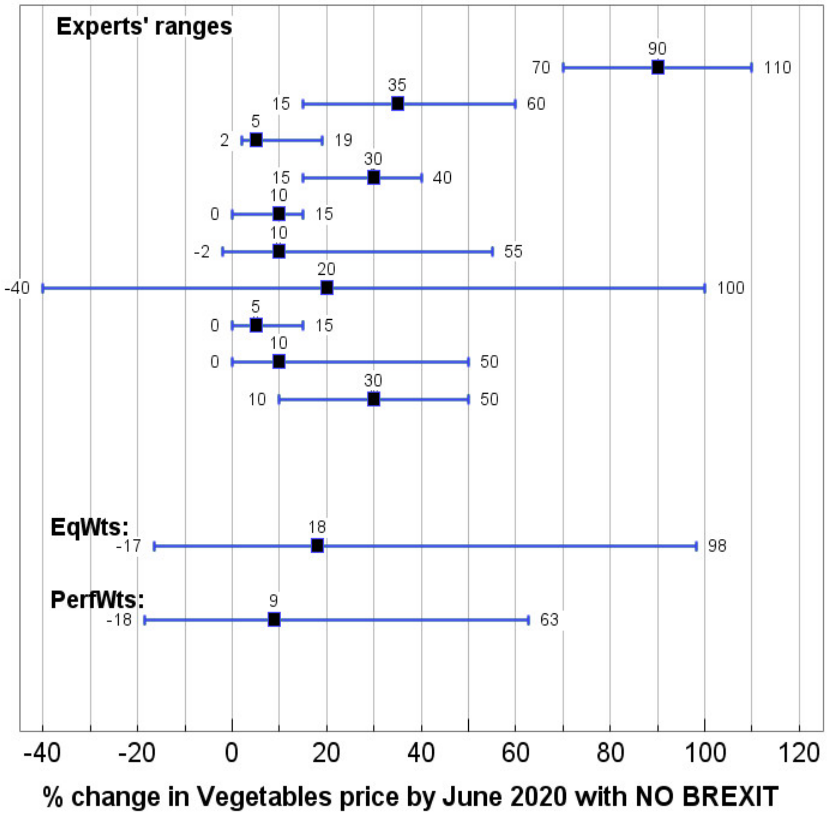
Experts' ranges
EqWts:
PerfWts:
-40 -20 0 20 40 60 80 100 120
% change in Vegetables price by June 2020 with NO BREXIT

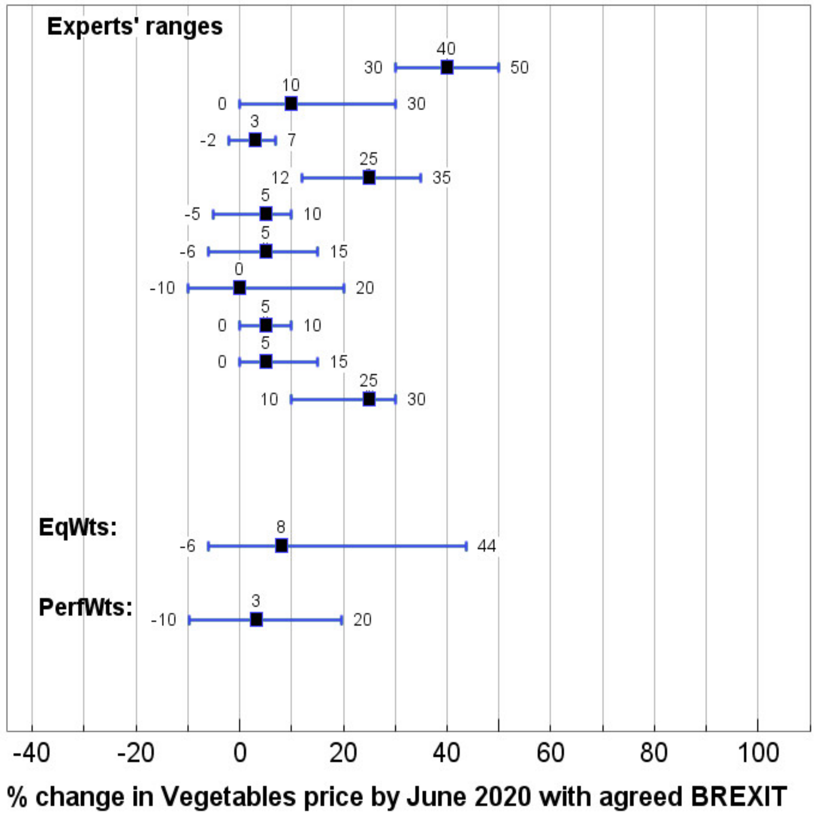
Experts' ranges
EqWts:
PerfWts:
-40 -20 0 20 40 60 80 100
% change in Vegetables price by June 2020 with agreed BREXIT

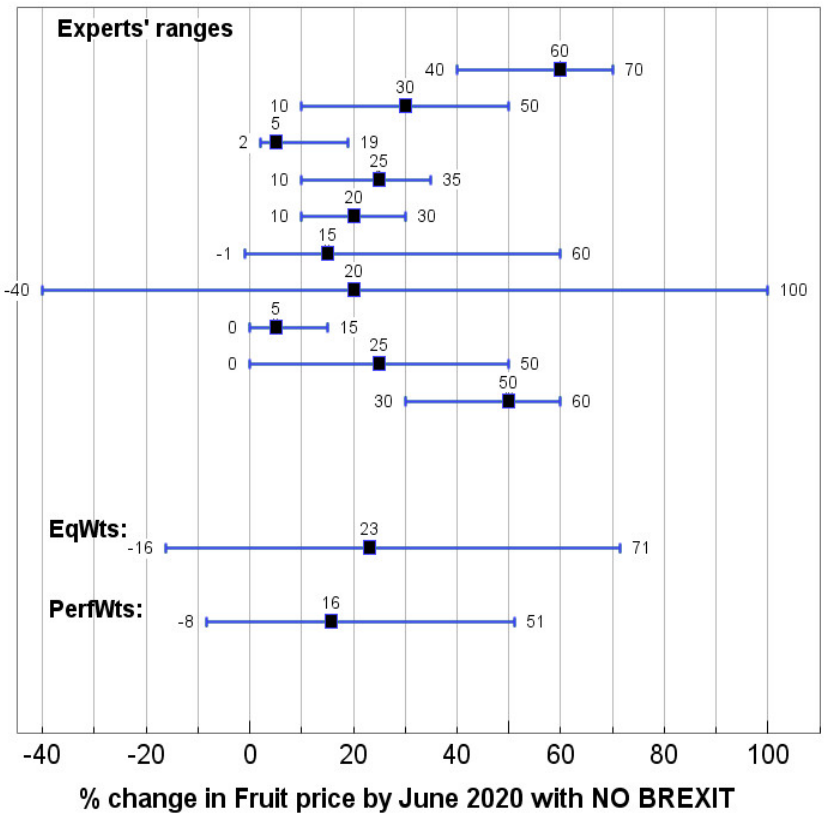
Experts' ranges
60
40 70
30
10 50
5
2 19
25
10 35
20
10 30
15
-1 60
20
-40 100
5
0 15
25
0 50
50
30 60
EqWts:
23
-16 71
PerfWts:
16
-8 51
-40 -20 0 20 40 60 80 100
% change in Fruit price by June 2020 with NO BREXIT

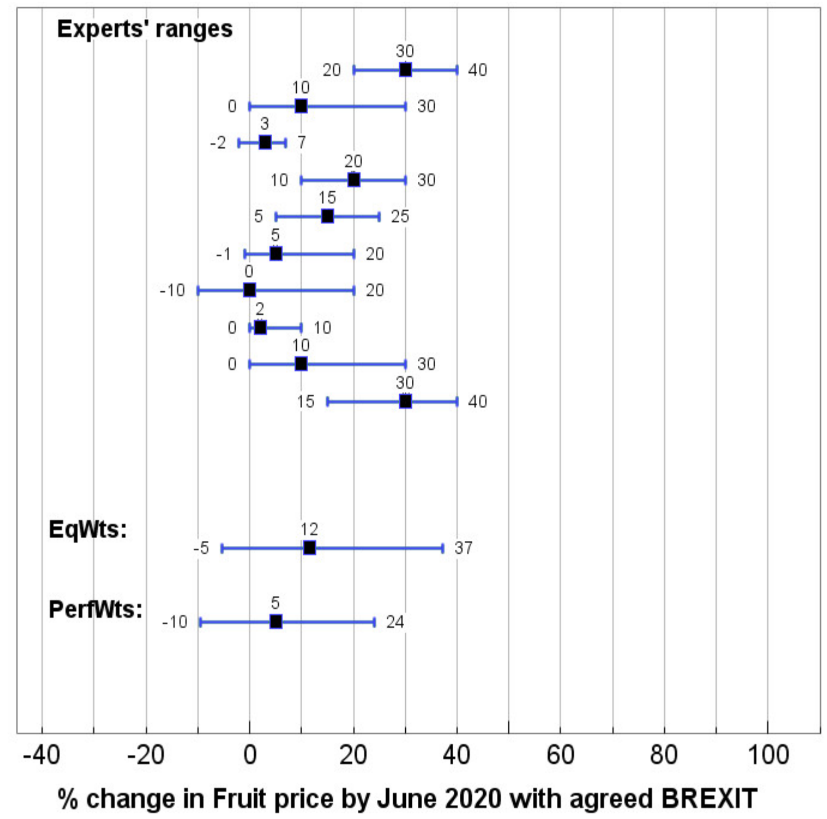
Experts' ranges
30
20 40
10
0 30
3
-2 7
20
10 30
15
5 25
5
-1 20
0
-10 20
2
0 10
10
0 30
30
15 40
EqWts:
12
-5 37
PerfWts:
5
-10 24
-40 -20 0 20 40 60 80 100
% change in Fruit price by June 2020 with agreed BREXIT

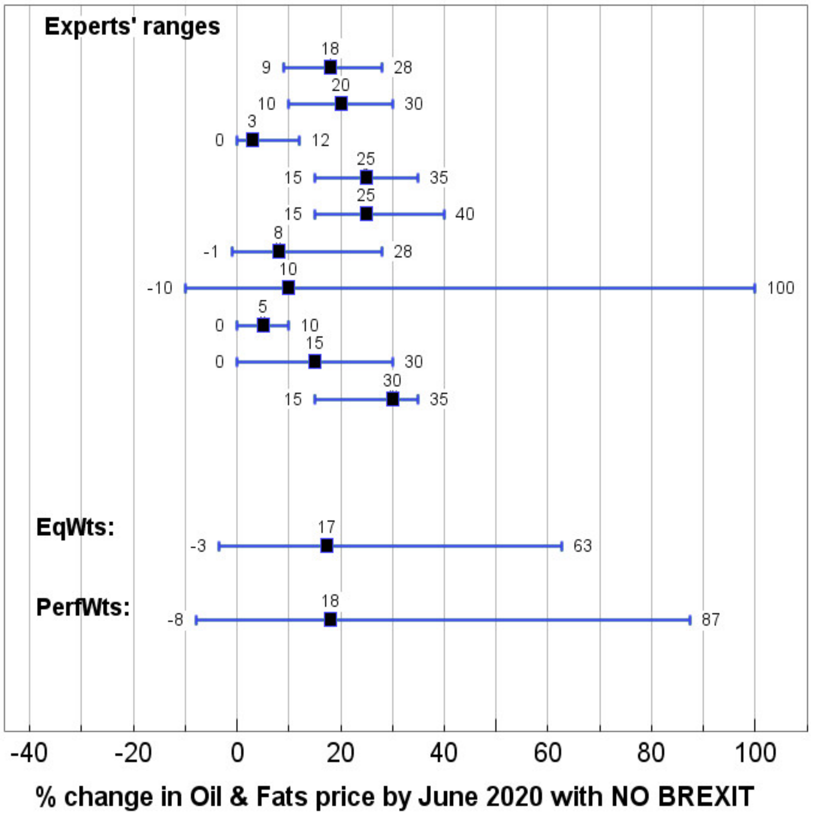

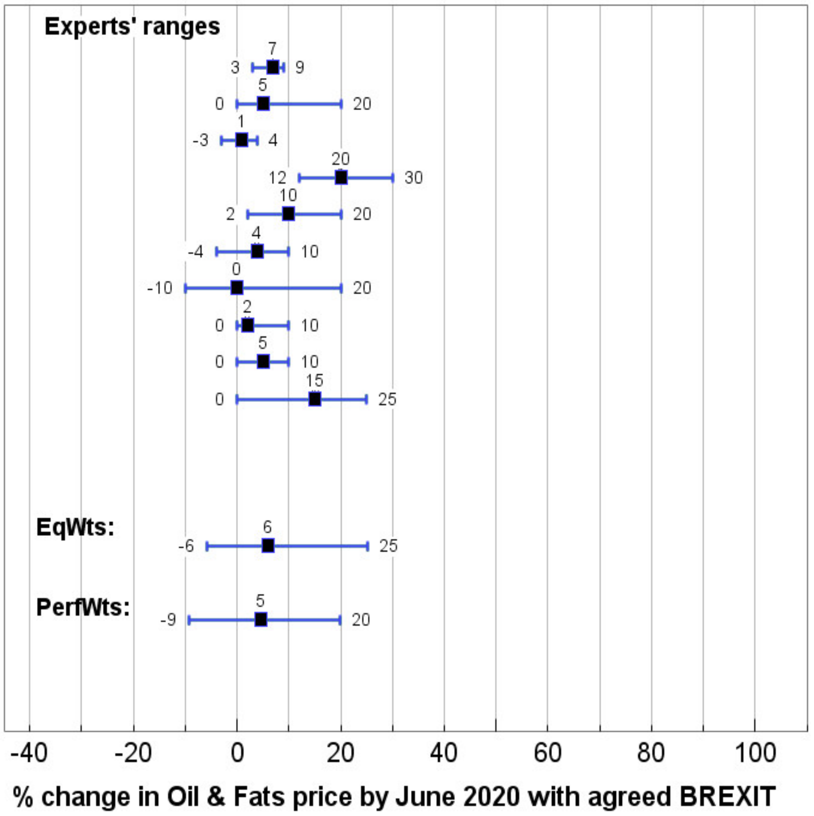

For peer review only - http://bmjopen.bmj.com/site/about/guidelines.xhtml

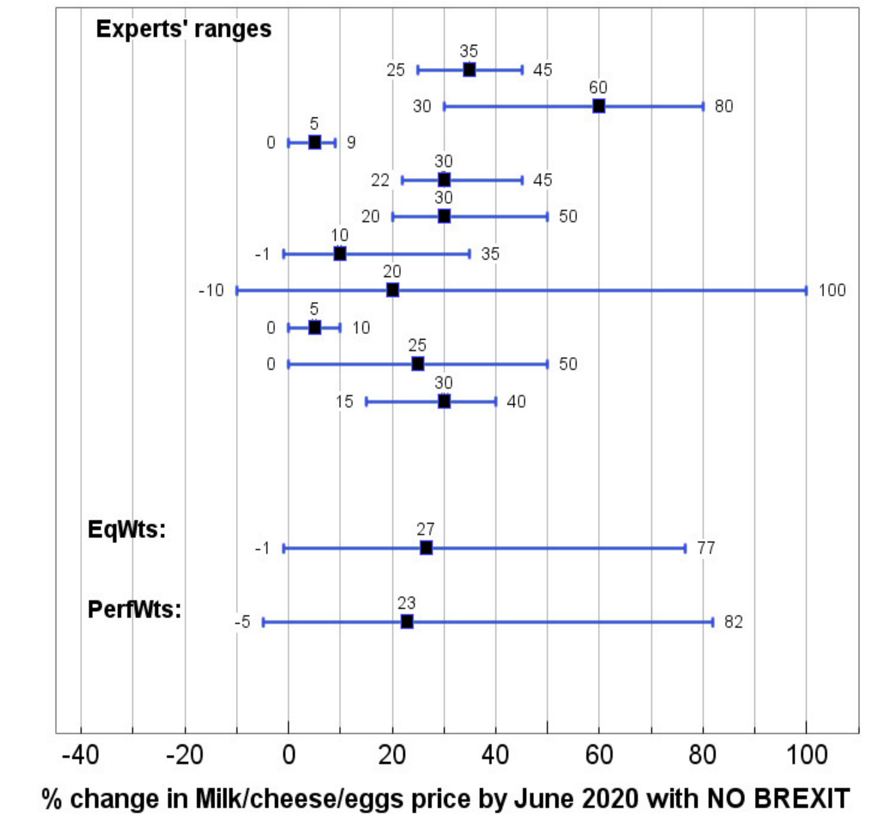
Experts' ranges
35
25 45
60
30 80
5
0 9
30
22 45
30
20 50
10
-1 35
20
-10 100
5
0 10
25
0 50
30
15 40
EqWts:
27
-1 77
PerfWts:
23
-5 82
-40 -20 0 20 40 60 80 100
% change in Milk/cheese/eggs price by June 2020 with NO BREXIT

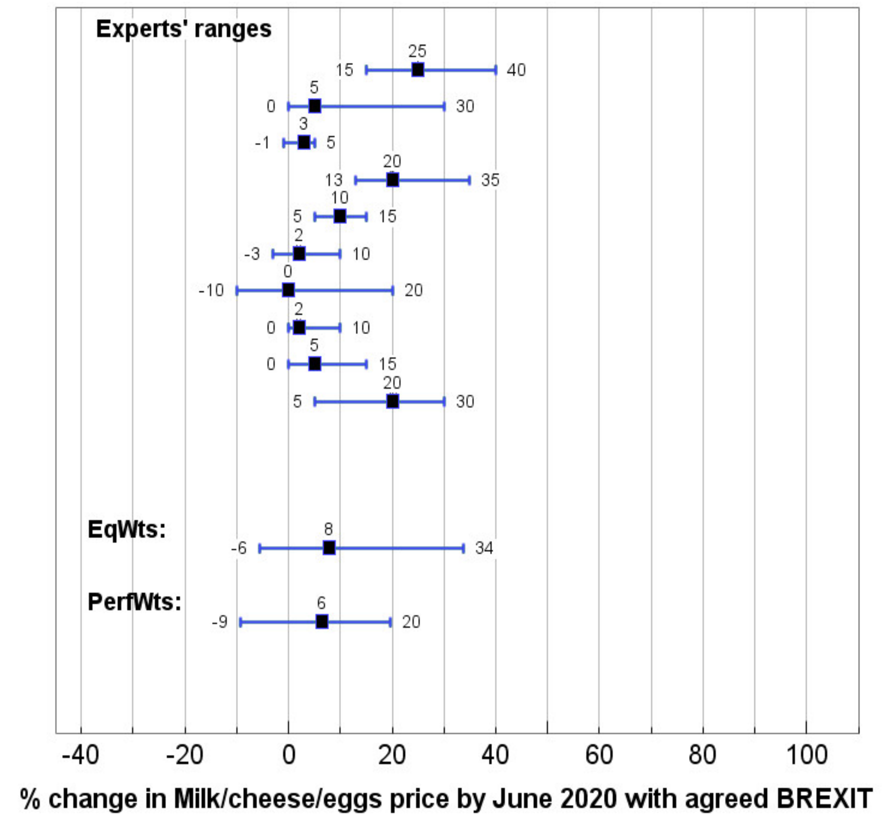
Experts' ranges
25
15 40
5
0 30
3
-1 5
20
13 35
10
5 15
2
-3 10
0
-10 20
2
0 10
5
0 15
20
5 30
EqWts:
8
-6 34
PerfWts:
6
-9 20
-40 -20 0 20 40 60 80 100
% change in Milk/cheese/eggs price by June 2020 with agreed BREXIT

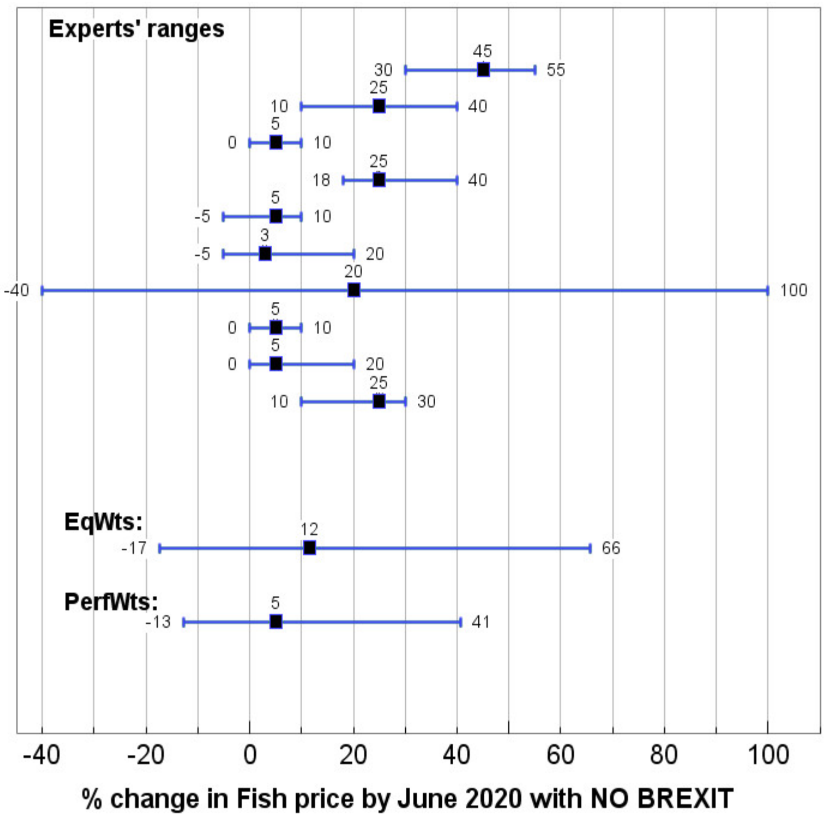

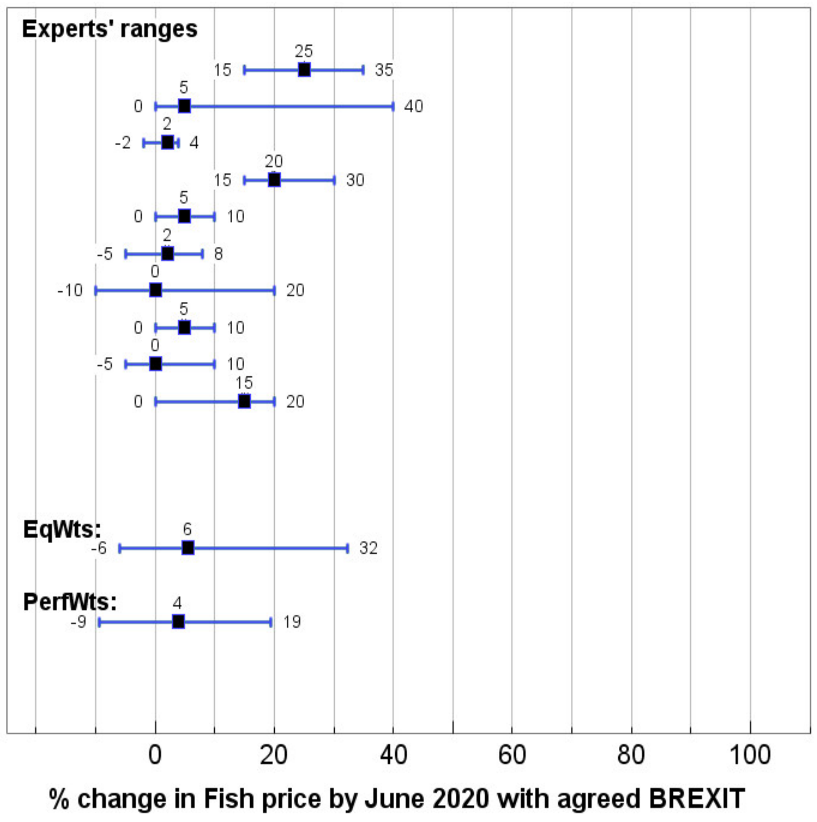

For peer review only - http://bmjopen.bmj.com/site/about/guidelines.xhtml

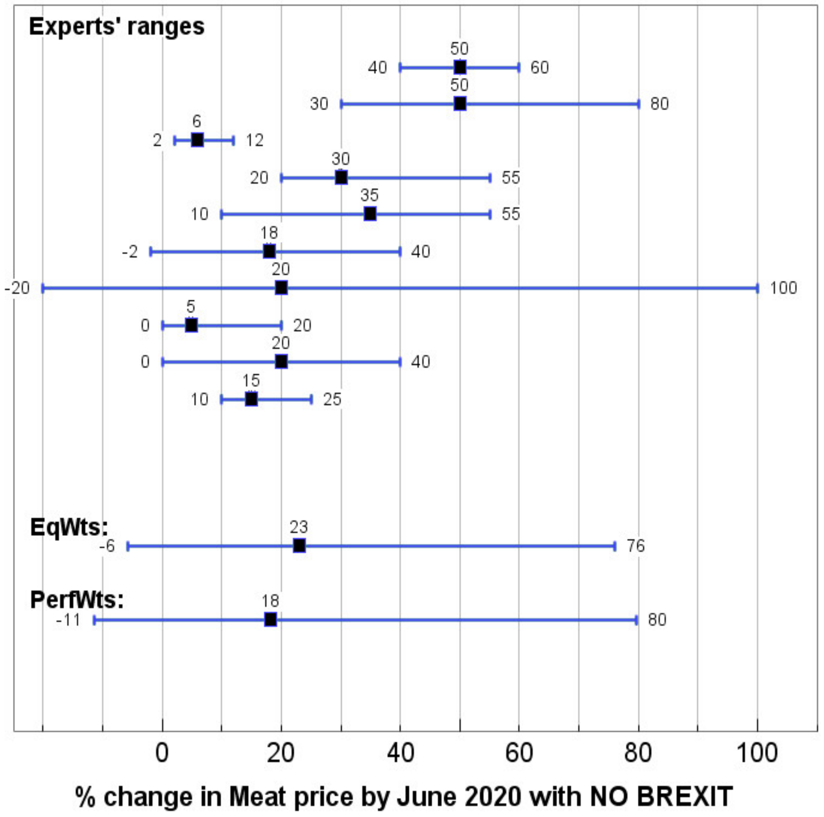
Experts' ranges
50
40 60
50
30 80
6
2 12
30
20 55
35
10 55
18
-2 40
20
-20 100
5
0 20
20
0 40
15
10 25
EqWts:
23
-6 76
PerfWts:
18
-11 80
0 20 40 60 80 100
% change in Meat price by June 2020 with NO BREXIT

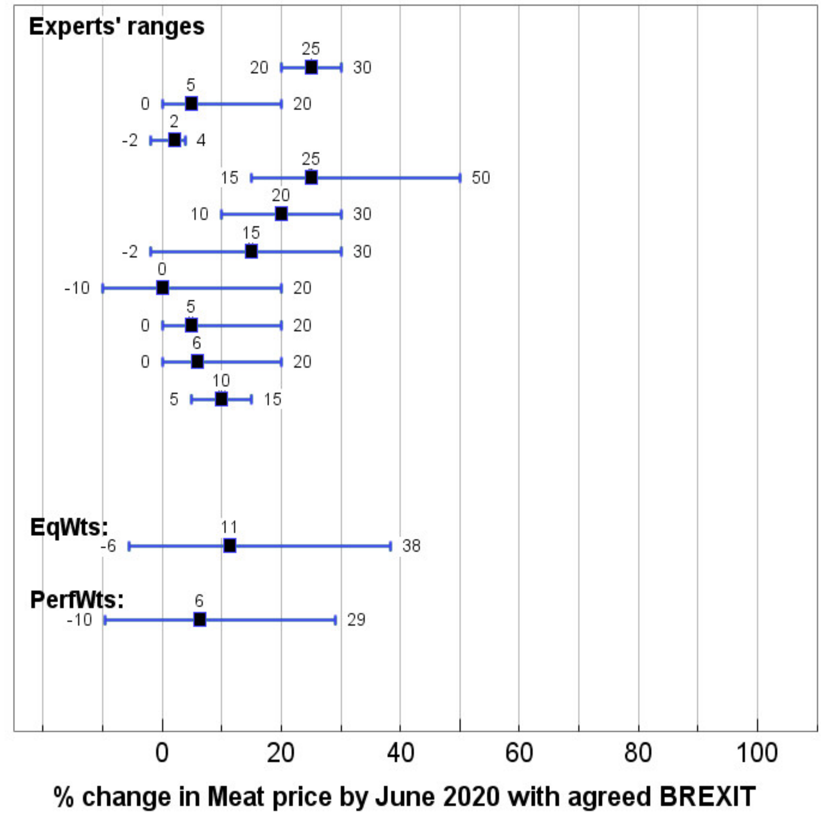
Experts' ranges
25
20 30
5
0 20
2
-2 4
25
15 50
20
10 30
15
-2 30
0
-10 20
5
0 20
6
0 20
10
5 15
EqWts:
11
-6 38
PerfWts:
6
-10 29
0 20 40 60 80 100
% change in Meat price by June 2020 with agreed BREXIT

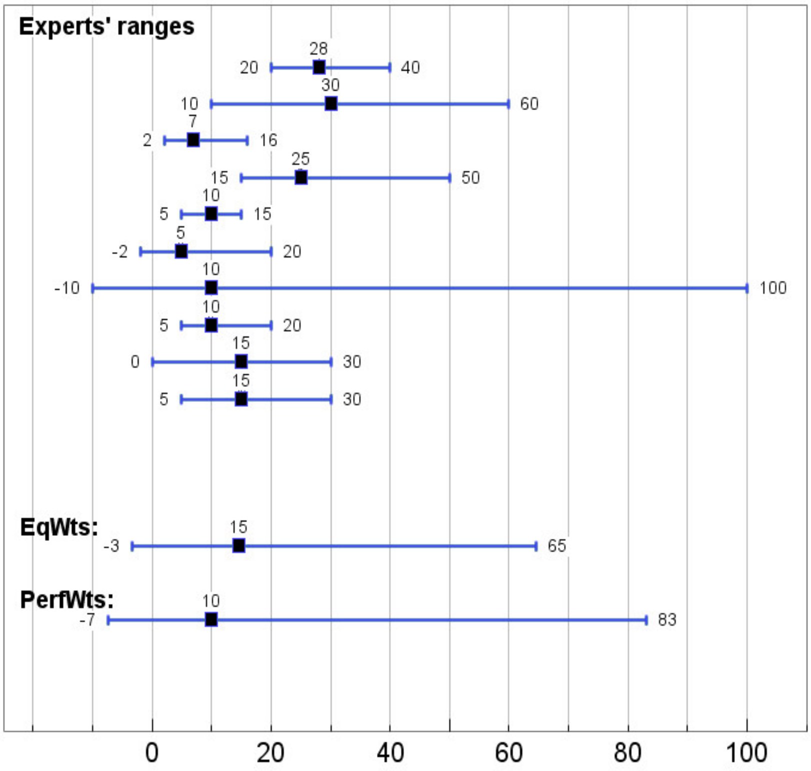

% change in Bread & Cereals by June 2020 with NO BREXIT

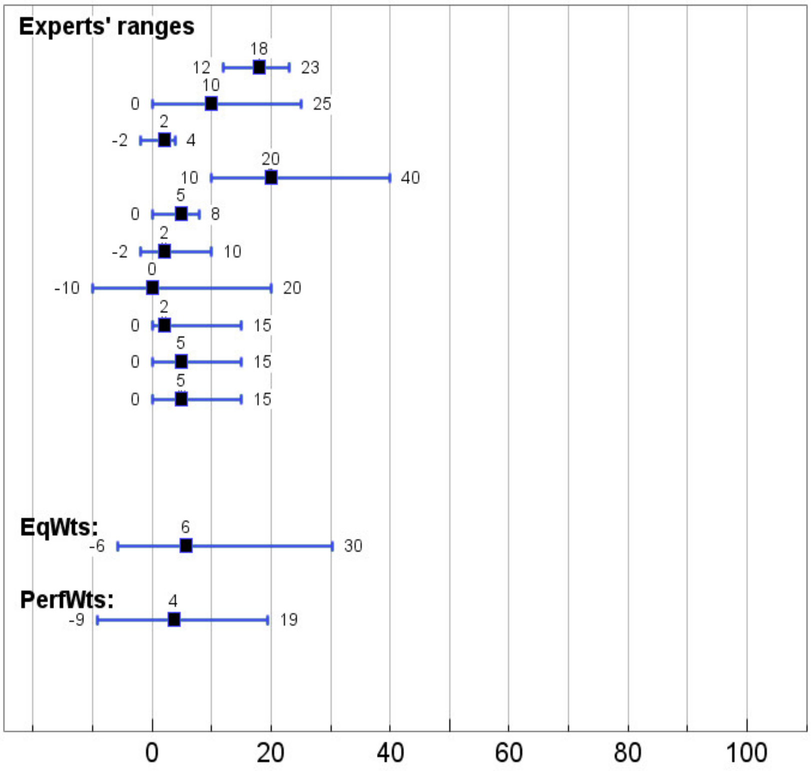

% change in Bread & Cereals by June 2020 with agreed BREXIT

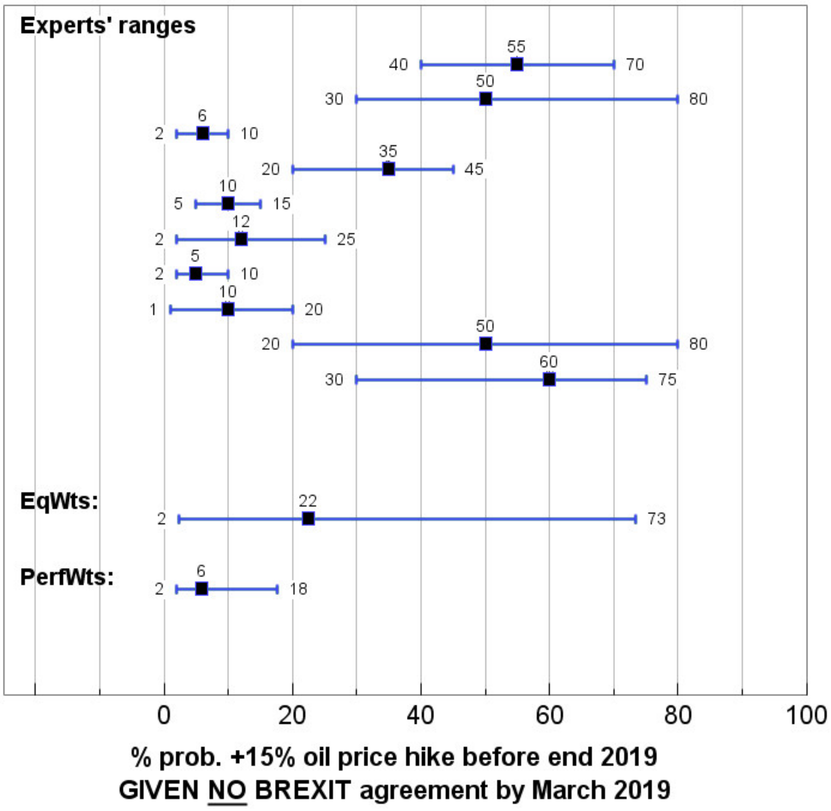

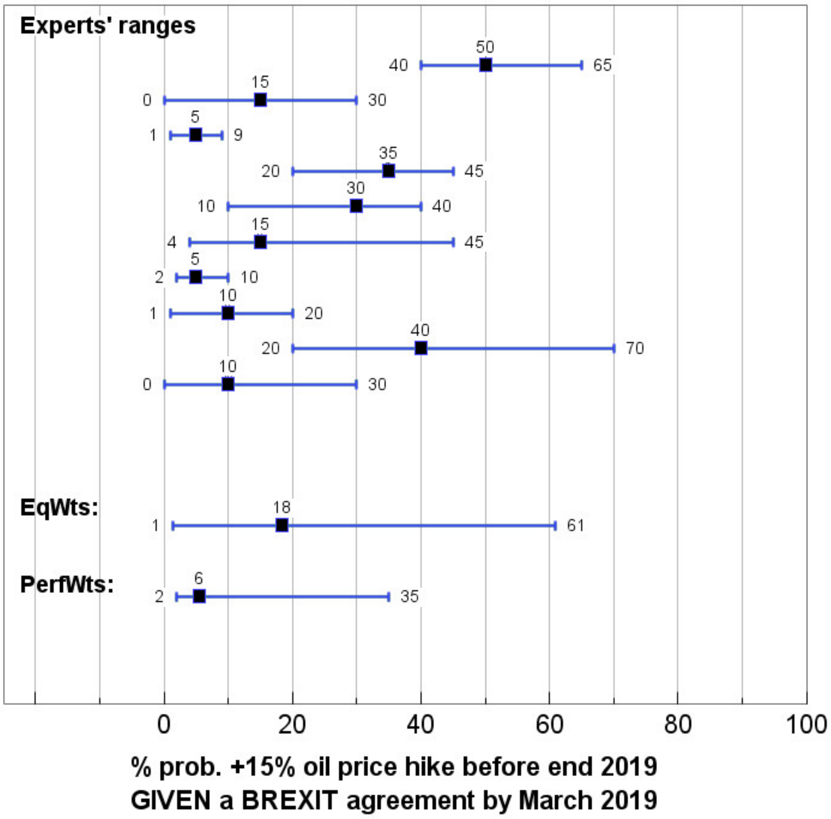

For peer review only - http://bmjopen.bmj.com/site/about/guidelines.xhtml

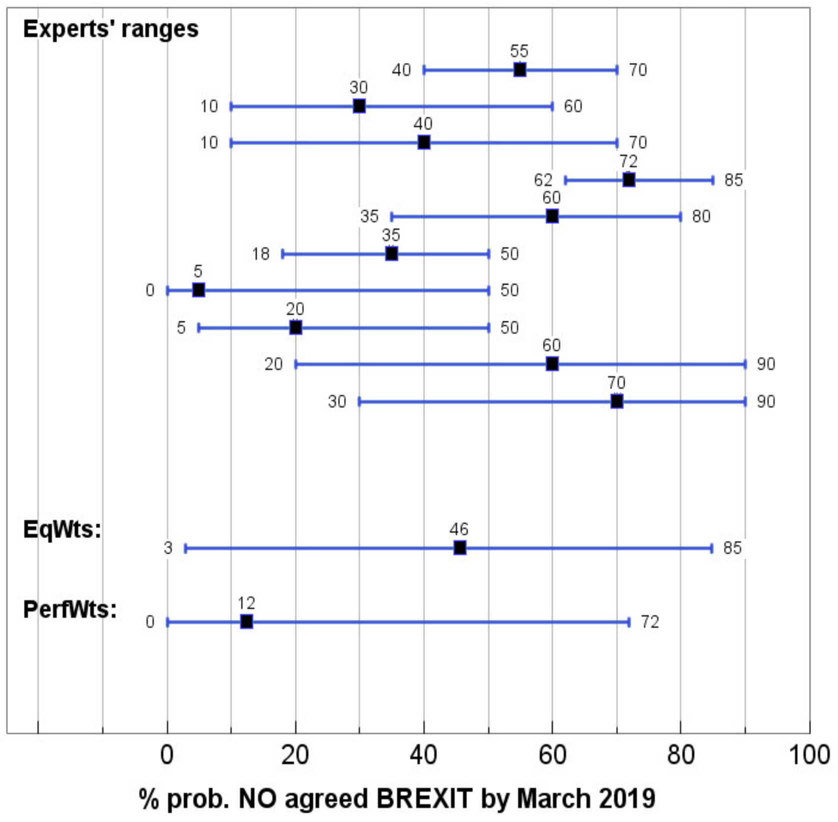

review only

For peer review only - http://bmjopen.bmj.com/site/about/guidelines.xhtml

Discussion of results

The elicitation was judged to be highly successful by the experienced facilitator (WPA).

As noted above, three of the ten scored experts achieved significant performance weights. The performance weighted (PW) aggregation solutions, as influenced by these three experts and their weights, reduced group-wide uncertainty spreads in most cases.

The following notes record immediate panel member verbatim comments on preliminary results from the elicitation, which were presented to the group on the final morning of the workshop; n.b. target item solutions, as presented in the previous section, were subsequently finalised after additional responses were received from some participants.

Comments recorded here are not attributed to individual participants.

Target item comments

1. *Food prices with and without a BREXT trade deal in place*
    a. *Bread and cereals*

- Agreed that there was expected uncertainty relating to the 'no BREXIT deal' graph so were not surprised by the bounds widening.
- One expert pointed out that this is an individual food category, if the question considered the entire CPI basket as a single variable then their own bounds would be smaller, but for this one category their wide bounds were truly meant to reflect their uncertainty.
- Bread and cereals category is a mix of domestic flours for biscuits and animal feed, imported cereals from Canada and the US. This is a compounded first order effect of what happens to the supply.
- Input of information that the UK imports Canadian flour for making bread, but uses domestic flour for items such as biscuits. Some experts said that they assumed most of our cereals came from USA which would have little impact from BREXIT deals.
- Noted that cereals are highly weather dependent, so regardless of BREXIT we have to consider crop yield dependent on environmental factors.
- How much are farmers going to plant? What's the weather like? What's the yield? What happens elsewhere in the world? If we don't have the protection of the EU, how do we meet the supply? What if we have to import 'poor quality' food, whether that means actually poor nutritional quality or 'poor' due to not meeting the technical specifications?
- Another factor that would affect the price is a heightened risk of job losses, which would result in less purchasing power in the population as a whole.
- In several of the instances we've looked at, then the substitution of goods also impacts the answer to this question. Potatoes and cereal don't necessarily substitute for each other, although they're both starches.
- With respect to potatoes, consider the fact that only half of the US crops are irrigated. If you have drought and flooding, then potato prices might shoot up in October [2019].
- One expert said they always tried to measure substitutes, for example if potatoes were blighted then they would expect more people to move to grains if their household was closer to food insecurity.

For peer review only - http://bmjopen.bmj.com/site/about/guidelines.xhtml

b. MEAT

- 70% of the cost of pork is attributed to wheat for feed; therefore this is heavily linked to the previous questions. However, most experts had not realised this so did not factor it into their judgements.
- One expert mentioned that the performance weights median seems too low because they reckon that we would see more climate change effects, even in the two-year short-term.
- Experts believed that with 'no BREXIT' deal then there would be a decrease in price for New Zealand lamb and South American meat due to the sudden influx in the market.
- Cattle are either dairy or meat assigned therefore no overlap in effects of meat and milk. Slaughtered dairy cows' meat is used in processed food such as pies and pasties, and not for joints of beef at the supermarket, etc. The calves born to initiate lactation are sold for meat, although some females may be retained to become milkers in future.

c. FISH

- Fish prices could go down quite a lot.
- UK fish comes predominantly from Norway; UK sells the fish it catches.
- Seas sovereignty is currently 40%. There was an expectation amongst the industry that this would increase to 80% after BREXIT, but that currently [July 2018] seems unlikely.
- We probably cannot police the seas as we have no military boats.

d. MILK, CHEESE, EGGS

- Lead time for hard cheese is anything up to one year, so would be a lag time effecting cheddar cheese.
- But soft cheeses, such as mozzarella, have a very short lead time and these are the cheeses used on pizzas and in ready meals; there are different lead times for each of the different cheese types.
- If milk subsidy disappeared, more farmers might turn to producing meat. How fast would the changes be with each of these?

e. OILS & FATS

- One expert was not happy that the aggregated answers for oils & fats are roughly the same as for the meats item – they believe that the median and uncertainty in meat should be much higher.
- Food oil is completely imported.
- Interconnected to petroleum oil due to biofuel industry.
- Price of crude oil directly affects the price of oil and fats.

f. FRUIT

- The UK imports 82% of fruit, a lot of these are non-EU countries; however, they have free EU trade agreements

g. VEGETABLES

- There seems to be more uncertainty associated with vegetables than with previous food categories in **either** situation (BREXIT deal vs. no-BREXIT deal) because of the inherent impacts of weather/seasonality.
- Most experts agree that uncertainty around vegetables is not as great as uncertainty around fruit because of the import numbers.
- Vegetable substitutions are vast. One expert hypothesised that the British public would be happy to drop certain supermarket fruits from diets, if these were too expensive. This is true for fish too, but the general consensus is that the public is much fussier about changing fish type than fruit type.
- Some crops (potatoes) have bulking phase in their growth cycle, so weather and the timing of rainfall, sun, etc. have a significant effect on yield.

For peer review only - http://bmjopen.bmj.com/site/about/guidelines.xhtml

h. SUGAR+JAM

- Sugar beet is grown in the UK in very focussed areas so flooding or rainfall in these areas has serious consequences (however, not sure this will change by 2020).
- Trade policy important for sugarcane and jam. One major producer of cane sugar is an advocate of BREXIT because of profit opportunity by reduction of cane tariff; the other major is a sugar beet producer.

i. COFFEE, TEA, COCOA

- In CPI terms, this category means instant and ground coffee or tea bags and loose tea sold in supermarkets, not drinks sold in shops.
- Huge market, all imported, subject to exchange rate and tariffs.
- Tea / coffee pricing can be controlled to some extent by different blending, e.g. by diluting Arabica.

j. SOFT DRINKS

- Relatively speaking, there seems to be much less future pricing uncertainty than for other foodstuffs.
- Soft drinks can easily recalibrate recipes and ingredients to accommodate changing prices of raw materials, e.g. sugar.
- One expert pointed out that they cannot recall Coca-cola or Pepsi changing their prices in our lifetime, which makes it seem like they are unlikely to change in the future

For peer review only

Appendix S1.1 Target items questionnaire

For each question, your three values should be unique (i.e. no ties), and should be in ascending order: i.e. Low < Median < High.

First, three questions about the probabilities of two potential major external factors that could influence Food prices.

TQ1a. This question concerns BREXIT

What is the probability there will be NO agreed EU-UK BREXIT deal by March 2019? Please give your range of judgments for the low-end, median and high-end probabilities of this happening, expressed as percentages in range 0 – 100%.		
**Low (5th)**	**Median (50th)**	**High (95th)**

Future Food Prices changes: outlook timeframe is June 2020.

TQ2a. This pair of questions concern *Bread & cereals* prices and BREXIT

GIVEN current quarterly Bread & Cereals spend per household is £141.55 AND GIVEN there is an agreed EU-UK BREXIT deal by March 2019, please give your judgments of the low-end, median and high-end percentage changes in Bread & Cereals spend per household by June 2020. (A decrease should be expressed as a negative percentage change).		
**Low (5th)**	**Median (50th)**	**High (95th)**

TQ2b.

GIVEN current quarterly Bread & Cereals spend per household is £141.55 AND GIVEN there is NO BREXIT deal by March 2019, please give your judgments of the low-end, median and high-end percentage changes in Bread & Cereals spend per household by June 2020. (A decrease should be expressed as a negative percentage change).		
**Low (5th)**	**Median (50th)**	**High (95th)**

For peer review only - http://bmjopen.bmj.com/site/about/guidelines.xhtml

TQ3a. This pair of questions concern Meat prices and BREXIT

GIVEN current quarterly Meat spend per household is £188.12 AND GIVEN there is an agreed EU-UK BREXIT deal by March 2019, please give your judgments of the low-end, median and high-end percentage changes in Meat spend per household by June 2020.
(A decrease should be expressed as a negative percentage change).

Low (5th)	Median (50th)	High (95th)

TQ3b.

GIVEN current quarterly Meat spend per household is £188.12 AND GIVEN there is NO BREXIT deal by March 2019, please give your judgments of the low-end, median and high-end percentage changes in Meat spend per household by June 2020.
(A decrease should be expressed as a negative percentage change).

Low (5th)	Median (50th)	High (95th)

TQ4a. This pair of questions concern *Fish* prices and BREXIT

GIVEN current quarterly Fish spend per household is £40.47 AND GIVEN there is an agreed EU-UK BREXIT deal by March 2019, please give your judgments of the low-end, median and high-end percentage changes in Fish spend per household by June 2020.
(A decrease should be expressed as a negative percentage change).

Low (5th)	Median (50th)	High (95th)

TQ4b.

GIVEN current quarterly Fish spend per household is £40.47 AND GIVEN there is NO BREXIT deal by March 2019, please give your judgments of the low-end, median and high-end percentage changes in Meat spend per household by June 2020.
(A decrease should be expressed as a negative percentage change).

Low (5th)	Median (50th)	High (95th)

For peer review only - http://bmjopen.bmj.com/site/about/guidelines.xhtml

TQ5a. This pair of questions concern *Milk, cheese & eggs* prices and BREXIT

GIVEN current quarterly Milk, cheese & eggs spend per household is £110.37 AND GIVEN there is an agreed EU-UK BREXIT deal by March 2019, please give your judgments of the low-end, median and high-end percentage changes in Milk, cheese & eggs spend per household by June 2020.<br>(A decrease should be expressed as a negative percentage change).
______ Low (5th) ______ Median (50th) ______ High (95th)

TQ5b.

GIVEN current quarterly Milk, cheese & eggs spend per household is £110.37 AND GIVEN there is NO BREXIT deal by March 2019, please give your judgments of the low-end, median and high-end percentage changes in Milk, cheese & eggs spend per household by June 2020.<br>(A decrease should be expressed as a negative percentage change).
______ Low (5th) ______ Median (50th) ______ High (95th)

TQ6a. This pair of questions concern *Oil & fats* prices and BREXIT

GIVEN current quarterly Oil & fats spend per household is £19.65 AND GIVEN there is an agreed EU-UK BREXIT deal by March 2019, please give your judgments of the low-end, median and high-end percentage changes in Oil & fats spend per household by June 2020.<br>(A decrease should be expressed as a negative percentage change).
______ Low (5th) ______ Median (50th) ______ High (95th)

TQ6b.

GIVEN current quarterly Oil & fats spend per household is £19.65 AND GIVEN there is NO BREXIT deal by March 2019, please give your judgments of the low-end, median and high-end percentage changes in Oil & fats spend per household by June 2020.<br>(A decrease should be expressed as a negative percentage change).
______ Low (5th) ______ Median (50th) ______ High (95th)

For peer review only - http://bmjopen.bmj.com/site/about/guidelines.xhtml

TQ7a. This pair of questions concern *Fruit* prices and BREXIT

GIVEN current quarterly Fruit spend per household is £94.94 AND GIVEN there is an agreed EU-UK BREXIT deal by March 2019, please give your judgments of the low-end, median and high-end percentage changes in Fruit spend per household by June 2020.<br>(A decrease should be expressed as a negative percentage change).
**Low (5th)** **Median (50th)** **High (95th)**

TQ7b.

GIVEN current quarterly Fruit spend per household is £94.94 AND GIVEN there is NO BREXIT deal by March 2019, please give your judgments of the low-end, median and high-end percentage changes in Fruit spend per household by June 2020.<br>(A decrease should be expressed as a negative percentage change).
**Low (5th)** **Median (50th)** **High (95th)**

TQ8a. This pair of questions concern *Vegetables* prices and BREXIT

GIVEN current quarterly Vegetables spend per household is £123.55 AND GIVEN there is an agreed EU-UK BREXIT deal by March 2019, please give your judgments of the low-end, median and high-end percentage changes in Vegetables spend per household by June 2020.<br>(A decrease should be expressed as a negative percentage change).
**Low (5th)** **Median (50th)** **High (95th)**

TQ8b.

GIVEN current quarterly Vegetables spend per household is £123.5 AND GIVEN there is NO BREXIT deal by March 2019, please give your judgments of the low-end, median and high-end percentage changes in Vegetables spend per household by June 2020.<br>(A decrease should be expressed as a negative percentage change).
**Low (5th)** **Median (50th)** **High (95th)**

TQ9a. This pair of questions concern *Sugar, jam etc* prices and BREXIT

For peer review only - http://bmjopen.bmj.com/site/about/guidelines.xhtml

GIVEN current quarterly Sugar, jam, etc. spend per household is £122.78 AND GIVEN there is an agreed EU-UK BREXIT deal by March 2019, please give your judgments of the low-end, median and high-end percentage changes in Sugar, jam, etc. spend per household by June 2020.
(A decrease should be expressed as a negative percentage change).

**Low (5th)**	**Median (50th)**	**High (95th)**

TQ9b.

GIVEN current quarterly Sugar, jam, etc. spend per household is £122.78 AND GIVEN there is NO BREXIT deal by March 2019, please give your judgments of the low-end, median and high-end percentage changes in Sugar, jam, etc. spend per household by June 2020.
(A decrease should be expressed as a negative percentage change).

**Low (5th)**	**Median (50th)**	**High (95th)**

TQ10a. This pair of questions concern *Coffee, tea & cocoa* prices and BREXIT

GIVEN current quarterly Coffee, tea & cocoa spend per household is £31.88 AND GIVEN there is an agreed EU-UK BREXIT deal by March 2019, please give your judgments of the low-end, median and high-end percentage changes in Coffee, tea & cocoa spend per household by June 2020.
(A decrease should be expressed as a negative percentage change).

**Low (5th)**	**Median (50th)**	**High (95th)**

TQ11b.

GIVEN current quarterly Coffee, tea & cocoa spend per household is £31.88 AND GIVEN there is NO BREXIT deal by March 2019, please give your judgments of the low-end, median and high-end percentage changes in Coffee, tea & cocoa spend per household by June 2020.
(A decrease should be expressed as a negative percentage change).

**Low (5th)**	**Median (50th)**	**High (95th)**

TQ12a. This pair of questions concern *Soft drinks etc.* prices and BREXIT

GIVEN current quarterly Soft drinks etc. spend per household is £82.75 AND GIVEN there is an agreed EU-UK BREXIT deal by March 2019, please give your judgments of the low-end, median and high-end percentage changes in Soft drinks etc. spend per household by June 2020. (A decrease should be expressed as a negative percentage change).		
**Low (5th)**	**Median (50th)**	**High (95th)**

TQ12b.

GIVEN current quarterly Soft drinks etc. spend per household is £82.75 AND GIVEN there is NO BREXIT deal by March 2019, please give your judgments of the low-end, median and high-end percentage changes in Soft drinks etc. spend per household by June 2020. (A decrease should be expressed as a negative percentage change).		
**Low (5th)**	**Median (50th)**	**High (95th)**

For peer review only

For peer review only - http://bmjopen.bmj.com/site/about/guidelines.xhtml

Appendix S1.2 Workshop Invitation

On 21 June 2018 at 20:20, Barons, Martine <Martine.Barons@warwick.ac.uk> wrote:

I recently invited you to take part in a research exercise on food prices as part of developing decision support for policymakers concerned with food insecurity, reliance on food banks etc.

It would be very helpful if you could let me know in the next day or two whether you are able to participate.

There is a minimum number of participants required for the research exercise to run, and if the exercise needs to be cancelled, I would like to give reasonable notice to those who have already offered their time and to Prof Willy Aspinall, who is in high demand and may be able to accommodate other bookings if we cancel.

Best wishes, Martine

Dr Martine J. Barons CMath MIMA AMInstP
Director of the Applied Statistics and Risk Unit
Department of Statistics
University of Warwick
C0.10 | Zeeman Building | University of Warwick | CV4 7AL
martine.barons@warwick.ac.uk e-mail
http://www2.warwick.ac.uk/fac/sci/statistics/staff/academic-research/barons Web page

---

From: Barons, Martine
Sent: 21 May 2018 16:39

Subject: Food Price scenario workshop invitation

I would like to invite you to participate in a structured expert elicitation exercise to be facilitated by Prof. Willy Aspinall at the University of Warwick, 4th – 6th July 2018. Invitees will be asked to suggest what, in their best judgement would be the effects of various scenarios, such as oil price changes, food scares, natural disaster, trade deals, on the UK price of foodstuffs as categorised in the Consumer Prices Index.

We will acknowledge your contribution unless you tell us you would prefer not to be credited and all discussions will be held under Chatham House rules, i.e. anyone who comes to the meeting is free to use information from the discussion, but is not allowed to reveal who made any comment.

We are looking for up to 20 people to give opinions on one or more foodstuffs. You have been invited since we believe you are expert in this area. If you are unable to attend yourself, please nominate a suitably qualified colleague.

For peer review only - http://bmjopen.bmj.com/site/about/guidelines.xhtml

Whilst we are unable to pay for your time, all UK travelling expenses will be covered, all meals provided and overnight accommodation provided on campus if required.

This structured elicitation forms part of ongoing research by myself and Professor Jim Smith on decision support for policies designed to ameliorate food poverty. We are collaborating with local authorities and so our interest is the effects on UK food prices.

Prof Willy Aspinall is Cabot Professor in Natural Hazards & Risk Science, University of Bristol.

The exercise will proceed as follows:
4th July: arrive in time for lunch. Afternoon briefing on protocol and discussion of scenarios and target questions. Evening, social dinner and overnight accommodation on campus if required.
5th July: Main structured elicitation exercise, evening meal, and accommodation on campus if required.
6th July: Feedback and final elicitation items if required. Leave after lunch.

I do hope you will be able to join us for this event.

Please let me know either way at your earliest convenience. Here is the form to capture dietary and accommodation requirements http://www2.warwick.ac.uk/fac/sci/statistics/asru/elicitation.

Dr Martine J. Barons **CMath MIMA AMInstP**
Director of the Applied Statistics and Risk Unit
go.warwick.ac.uk/ASRU
Department of Statistics |University of Warwick
C0.10 | Zeeman Building |University of Warwick | CV4 7AL
martine.barons@warwick.ac.uk e-mail
go.warwick.ac.uk/MJBarons Web page

For peer review only

For peer review only - http://bmjopen.bmj.com/site/about/guidelines.xhtml

Appendix S1.3 Briefing note sent to participants

Expert Elicitation and Judgment Pooling using the Cooke Classical Model and EXCALIBUR

Short briefing note for participants in an Expert Elicitation

Prepared by Willy Aspinall

July 2018

Important statement

In this note, a formal mathematical process is described for deriving objective differentiating weights for a group of experts in order to pool their opinions on questions of interest to the project in an optimal way. This calibration is based on empirical testing of individuals' abilities to provide informative uncertainty distributions capturing known realization values for certain related subject-matter quantities. The resulting calibration scores are NOT a measure of knowledge, scholarship or intelligence – but simply furnish a rational basis for setting out an understanding - for decision-support - of how uncertain parameters may be represented when alternative methods or solutions are not available.

1 Essentials

Expert opinion is almost invariably sought when technical uncertainty impacts on an important decision process. Because such uncertainty is ubiquitous in scientific knowledge - if it were not, any decision related to the issue at hand would be obvious – there is the inescapable corollary that the experts themselves cannot be absolutely certain, and thus it is extremely unlikely they will ever be in total agreement with one another. This is especially true where such uncertainty is substantial, or where the consequences of the decision are particularly serious or onerous.

In circumstances where scientific uncertainty impinges on the determination of an issue, soliciting expert advice is not new. Generally, however, this has been pursued on an informal basis, and such an unstructured approach is rarely, if ever, found entirely satisfying to all parties. Neither is it likely to be immune to legitimate criticism, from one side or another. To counteract these shortcomings, a *structured* expert judgment elicitation refers to the deliberate effort to subject the whole process to transparent methodological rules, with the goal of treating expert judgments as scientific data in a formal decision process.

Various methods for assessing and combining expert uncertainty are available in the literature. Some advocate a group decision-conferencing consensus approach for eliciting opinions, for instance, but other approaches exist for carrying out this process. Notable amongst these is the expert weighting procedure known as the Classical Model, formulated by Cooke (1991), which can be implemented with the computer package EXCALIBUR to analyse opinions obtained through a structured elicitation procedure. The unique feature of this approach is that it is the only one in which distinct weights are given to individual experts, based statistically on their ability to judge uncertainties as determined empirically by performance metrics derived from control questions; the theoretical basis and

For peer review only - http://bmjopen.bmj.com/site/about/guidelines.xhtml

principles of the Classical Model are expounded in Cooke (1991), and a recent summary of case histories using the procedure is given in Cooke and Goossens (2008).

1.1 The EXCALIBUR Procedure

The main steps in the procedure for applying the EXCALIBUR approach in practice can be summarised as follows:

- A group of experts are selected.
- Experts are elicited individually regarding their uncertainty over the results of possible measurements or observations within their domain of expertise.
- Experts also assess variables within their field, the true values of which are known or become known post hoc.
- Experts are treated as statistical hypotheses and are scored with regard to statistical likelihood (often called 'calibration') and informativeness.
- Scores are combined to form weights. These weights are constructed to be 'strictly proper scoring rules' in an appropriate asymptotic sense: experts receive their maximal expected long-run weight by, and only by, stating their true degrees of belief. With these weights, statistical accuracy strongly dominates informativeness – one cannot compensate poor statistical performance by very high information.
- Likelihood and informativeness scores are used to derive performance- based weighted combinations of the experts' uncertainty distributions.

The key feature of this method is the performance-based combination of expert uncertainty distributions. When it comes to attempting to resolve differences in expert judgments, people who seek to find a harmony of views by conciliation can be disconcerted by this approach, but extensive experience overwhelmingly confirms that experts grow to favour it because its performance measure are entirely objective and amenable to diagnostic examination.

1.2 Combining expert assessments to form a Decision Maker

A combination of expert assessments is often referred to as a "decision maker" (DM), in the sense of linear pooling. The steps in the process by which one can arrive at a decision maker outcome are summarised and illustrated schematically in Fig. 1. On the left-hand side of this diagram, hypothetical examples of the responses of three different experts to three seed questions are depicted, showing how their calibration can vary, in relation to the true realization value for the seed item, and how their information can also vary, generally from expert-to-expert, rather than within experts. Note that each expert is required to provide a fixed number of quantiles (usually three) to express his or her degree of belief in their judgment of the seed item value and the credible interval within which it should fall in their opinion.

With a set of several seed items (usually about ten in number), a group of experts can be ranked according to their individual calibration and information scores, and then according to the weights overall, as determined by the product of calibration and information scores. With these latter weights

For peer review only - http://bmjopen.bmj.com/site/about/guidelines.xhtml

to hand, it is then possible to elicit from the same group of experts their quantile-based distributions for items of interest (i.e. for questions for which an expert consensus is sought), and these individual response distributions can be linearly pooled, applying the individual weights. It should be noted that a weighted combination distribution, obtained in this way, is seldom if ever identical to the distribution of any one contributing expert, but does represent a rational consensus of the information provided by members of the group as a whole, differentiated by their performance on the seed items.

The Classical Model is essentially a formal method for deriving the requisite weights for a linear pool in which, as just noted, these weights are expressed as the product of an individual's calibration and information scores. "Good expertise" corresponds to good calibration (high statistical likelihood the expert's distributions reflect true values) and superior information. Strong weights reward good expertise, and pass these virtues on to the decision maker.

The reward aspect of weights is very important. An expert's influence on the decision maker should not appear haphazard, and he/she should be discouraged from attempting to game the system by tilting his/her assessments to achieve a desired outcome. Thus, it is necessary to impose a strictly proper scoring rule constraint on the weighing scheme. Roughly speaking, this means that an expert achieves his maximal expected weight by, and only by, stating assessments in conformity with his/her true beliefs.

2 Remarks

In the Classical Model, calibration and information are combined to yield an overall or combined score with the following attributes:

1. Individual expert assessments, realizations and scores can be recorded. This enables any reviewer to check the application of the method, in compliance with the principle of **accountability / scrutability.**
2. Performance is measured and hopefully validated, in compliance with the principle of **empirical control.** An expert's weight is determined by performance on seed items.
3. The score is a long run proper scoring rule for average probabilities, in compliance with the principle of **neutrality.**
4. Experts are treated equally, prior to the performance measurement, in compliance with the principle of **fairness.**

Whilst expert names and qualifications should be part of the documentation of every expert judgment study, they are not usually associated directly with identifiable individual assessments in the open literature. The experts' reasoning is always recorded and that is sometimes published as expert rationales.

There is no mathematical theorem which states that either item weights or global weights will out-perform equal weights or out-perform the best expert. Indeed, it is not difficult to construct artificial examples where this is not the case. Selecting which of these weighting schemes to use is a matter of experience. In practice, global weights are used unless item weights perform markedly better.

Of course, there may be other ways of defining expert weights that perform better, and indeed there might be better performance measures. But, good performance on a one-off basis for a single individual data set is not convincing. What is convincing is good performance on a large diverse data

For peer review only - http://bmjopen.bmj.com/site/about/guidelines.xhtml

For peer review only

set, such as the TU Delft expert judgment database (Cooke and Goossens, 2008; Colson and Cooke, 2017). In practice a method should be easy to apply, easy to explain, should do better than equal weighting and should never do something ridiculous.

More than one hundred different expert elicitations involving seed variables have been performed to date. These are all studies performed under contract for a problem owner, and reviewed and accepted by the contracting party. In most cases the results have been published. Given the body of experience with structured expert judgment that has now accumulated, the performance-based Classical Model approach is well established: as mentioned earlier, simply using equal weights for scientific uncertainty quantification no longer seems to be a convincing alternative.

Colson and Cooke (2017) have updated the TU Delft structured expert judgment database with data from 33 recent professionally-contracted Classical Model studies, and have evaluated the Classical Model's performance relative to other expert aggregation models. Performance weighting outperformed equal weighting in all but 1 of the 33 studies in-sample. For out-of-sample testing of the sets of calibration variables, overall the information of performance-based combinations is typically double that of equal weights combinations. Colson and Cooke (2017) propose an Out-of-Sample Validity Index based on averaging the product of statistical accuracy and information over all training sets sized at 80% of the calibration set. Performance weighting outperforms equal weighting on this Out-of-Sample Validity Index in 26 of the 33 studies; the probability of 26 or more successes on 33 trials -- if there were no difference between performance weighting and equal weighting -- is 0.001.

This experience shows that in the great majority of cases, the Classical Model performance-based combination of expert judgments gives more informative and statistically more accurate results than either the best individual expert or the 'equal weights' combination of all expert distributions. Upon reflection, it is evident that equal weighting has a very serious drawback. As the number of experts increases, the equal weight combination typically becomes increasingly diffuse, until it represents no one's belief and is useless for decision support. This is frequently seen as the number of experts exceeds, say, eight. The viability of equal weighting is maintained only by severely restricting the number of experts who will be treated equally, leaving others outside the process. It appeals to a sort of one-man-one-vote consensus ideal. Progress in science, however, is driven by rational consensus.

Ultimately, consensus is an equilibration of power; in science, it is not the power of the ballot but the power of arguments that counts (Kurowicka and Cooke, 2006), and this can be made manifest through the Classical Model / EXCALIBUR structured elicitation procedure.

Bibliography

Aspinall, W.P., 2006. Structured elicitation of expert judgement for probabilistic hazard and risk assessment in volcanic eruptions. In: Statistics in Volcanology (eds. H.M. Mader, S.G. Coles, C.B. Connor and L.J. Connor) - Special Publications of IAVCEI No. 1; London, The Geological Society for IAVCEI: 15-30.

Brown, A.J. and Aspinall, W.P., 2004. Use of expert elicitation to quantify the internal erosion processes in dams. Proceedings of the British Dam Society Conference, Thomas Telford, pp 282-297

Colson, A. R. and R. M. Cooke (2017). Cross validation for the classical model of structured expert judgment. Reliability Engineering and System Safety 163: 109-120.

For peer review only - http://bmjopen.bmj.com/site/about/guidelines.xhtml

Cooke, R. M., 1991. Experts in Uncertainty - Opinion and Subjective Probability in Science. Environmental Ethics and Science Policy Series. Oxford University Press, ISBN 0195064658.

Cooke, R.M., 2008. Guest Editorial, Special Issue on Expert Judgment. Reliability Engineering & System Safety. In press, corrected proof. doi:10.1016/j.ress.2007.03.001

Cooke, R.M. and Goossens, L.L.H.J., 2008. TU Delft expert judgment data base. Reliability Engineering & System Safety. In press, corrected proof. doi:10.1016/j.ress.2007.03.005.

Goossens L., Cooke R. and Kraan B. (1998) Evaluation of weighting schemes for expert judgment studies. PSAM4 Proceedings, eds. A. Mosleh and R.A. Bari. Vol. 4. Springer, 1937-1942.

Kurowicka, D. and Cooke, R., 2006. *Uncertainty Analysis with High Dimensional Dependence Modelling*. Wiley, Series in Probability and Statistics, Chichester: 284pp.

Fig. 1 Schematic chart showing how experts responses are calibrated against (multiple) seed

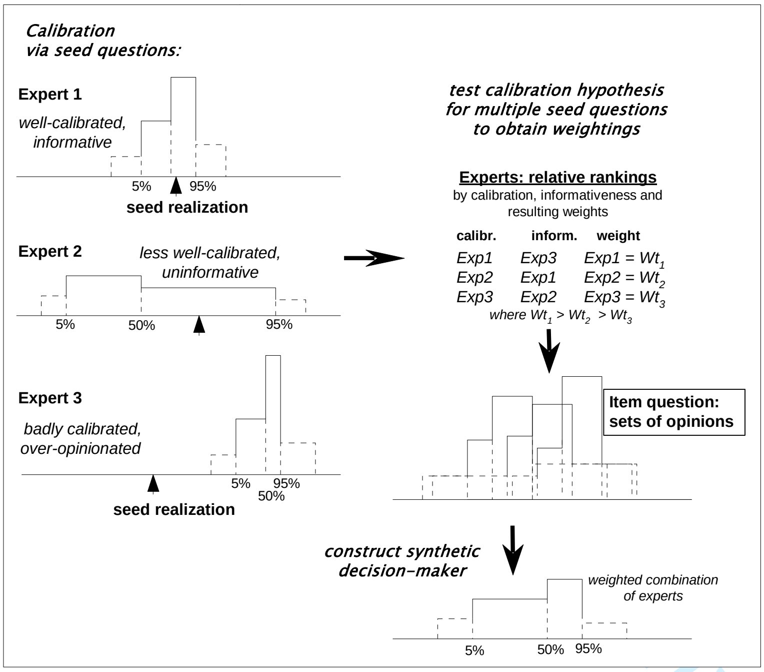

questions at given quantiles to produce performance-based weights; these are then used to pool the experts' opinions on corresponding quantiles for target questions which the problem owner wants quantified.

For peer review only - http://bmjopen.bmj.com/site/about/guidelines.xhtml

Supplementary Information – File S2

Barons, M. and Aspinall, W. **Anticipated impacts of Brexit scenarios on UK food prices and implications for policies on poverty and health: a structured expert judgement approach**

UNINET

UNINET enables efficient calculation of Bayes rule equations and rapid parameter conditionalization for making probabilistic inferences from observations. Conditionalizing a large network on multiple disparate observations and propagating this information through a multi-nodal model is analytically challenging; heuristics and simple statistical models can often lead us astray. BNs not only solve particular problems of information synthesis, they also provide a basis for reasoning probabilistically and testing model assumptions.

The main features of UNINET (distinguishing it from other packages) are that it supports non-parametric continuous or discrete BBNs, supplemented with functional nodes. Probabilistic nodes may be assigned arbitrary continuous or discrete distributions. UNINET has the valuable attribute of accepting Decision Maker results directly from EXCALIBUR output. Thus, Classical Model DM quantile histograms can be compiled into minimum information distribution form, without pre-processing, and used for propagating uncertainties into the desired analysis calculations.

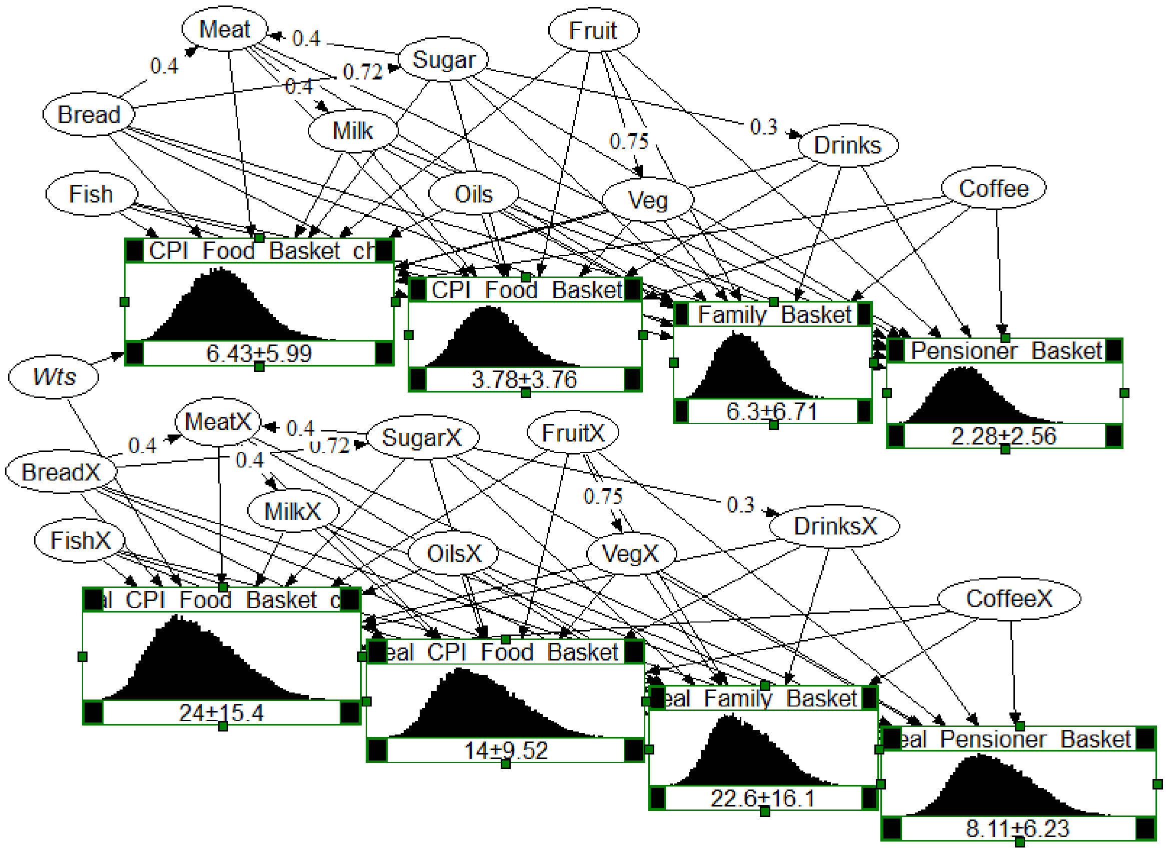

Figure A1 Bayes Net calculated output node distributions for percentage change in CPI Food Basket cost for Brexit Deal and No deal scenarios (LH pair of histograms) and for cost change in CPI Food Basket and two household cases in £, for the same Brexit scenarios. Each histogram panel reports the mean ± 1 s.d. change for that target item; other distribution statistics are output numerically by UNINET. Note hints of long upper tail skewness in every case (see text for discussion).

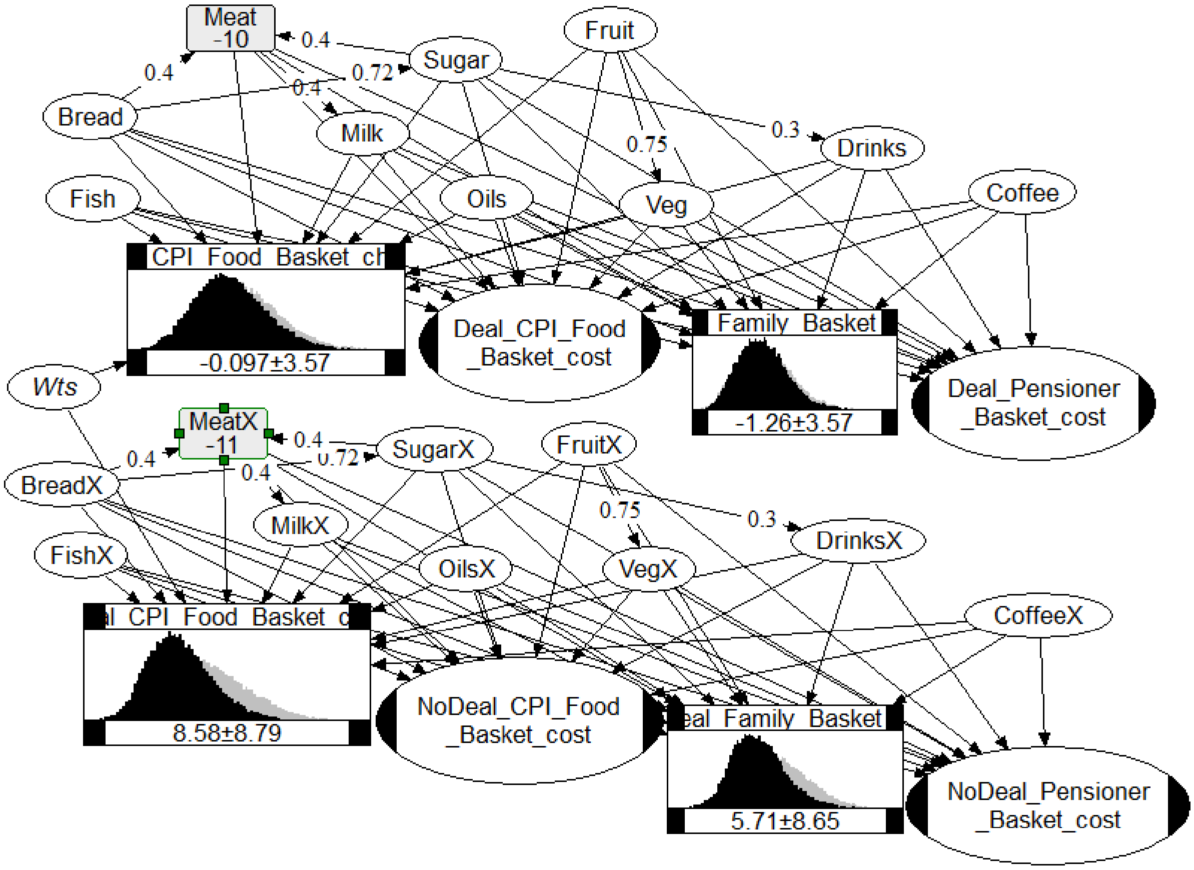

Figure A2 Bayes Net for Food baskets prices conditionalized on projected 5th percentile Meat (Deal: -10% change) and MeatX (No deal: -11% change) costs. The resulting updated target node distributions, due to conditionalizing Meat costs, are shown in black, the original model unconditionalized distributions are shown in grey.

A valuable attribute of UNINET, of particular benefit in the present study, is its capability to allow specific value conditioning on any number of probabilistic nodes. Analytic conditioning comprises either: (a) conditioning on a single value, which applies only for probabilistic nodes; or (b) sample-based conditioning, by which means the user can conditionalize on specific value intervals (for probabilistic and functional nodes). Thus, UNINET can model an empirical multivariate distribution by building a joint density function, which can be analytically conditioned. This capability is used here for conducting a pair of example sensitivity tests, based on selected Meat cost changes.

The UNINET BN is re-run with two food category nodes (deal Meat and no-deal MeatX) conditionalized to their 5th percentile levels from the elicitation distributions (-10% and -11%, respectively). In this example, we examine the effects of fixing these two nodes on the deal and no-deal outcome CPI basket percentage change and on the family-of-four basket cost, respectively. The resulting updated target node distributions, due to conditionalizing Meat costs, are shown on Figure A2.

Next, we run the BN with Meat (Deal) and MeatX (No deal) conditionalized to their elicited 95th percentile changes, i.e. +29% and +80%, which represent price increases well above the

elicited medians, respectively (Figure A3). Under the Brexit no-deal scenario: the CPI Basket mean change would be +44.0% (cf +24.0%) %)

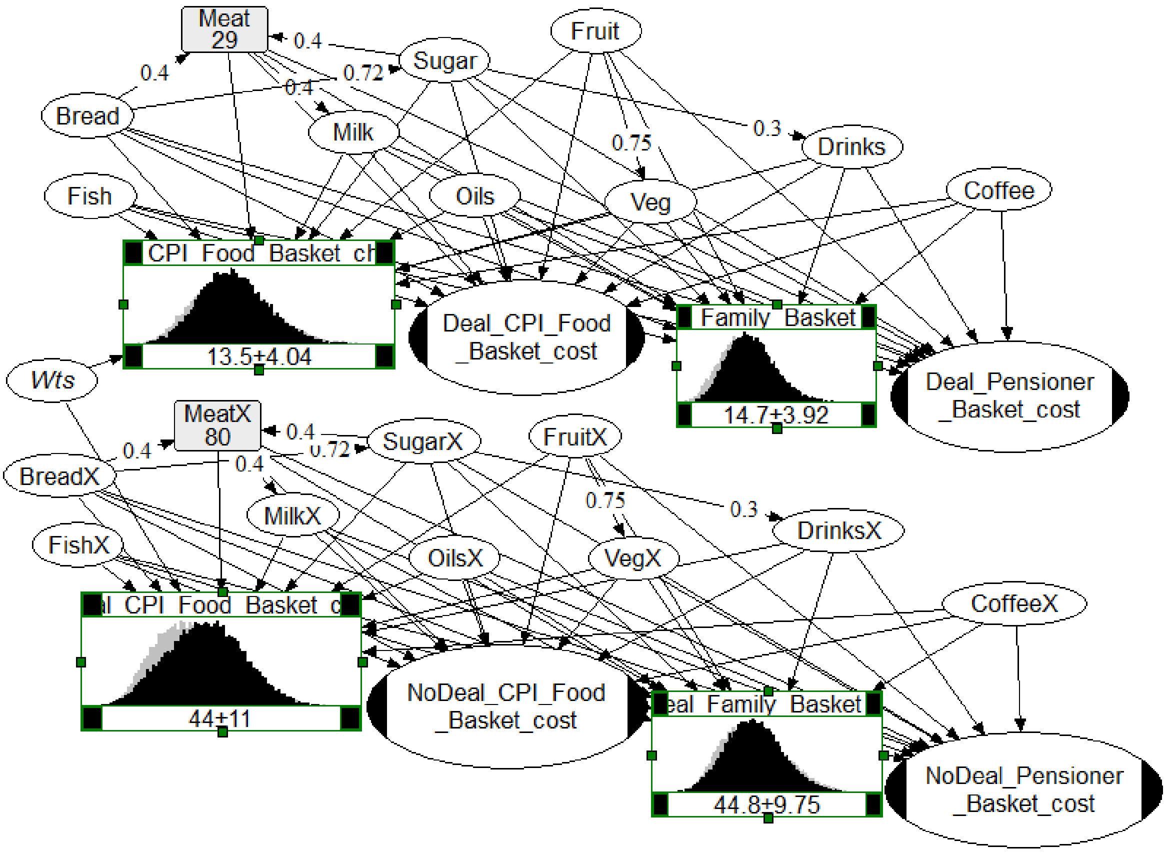

Figure A3 Bayes Net for Food baskets prices conditionalized on projected 95th percentile costs for Meat (Deal: +29% change) and MeatX (No deal: +80% change). The resulting updated target node distributions, due to conditionalizing Meat costs, are shown in black, the original model unconditionalized distributions are shown in grey.

For the what-if scenario where Meat prices are anchored to their projected 5th percentile values (Figure A2), the resulting Basket distribution shapes are bulk shifted to lower values, while those for the 95th percentile sensitivity test are moved higher (as might be expected, given certain other food prices are correlated with Meat price). Moreover, the standard deviations on the means are universally smaller under these Meat price sensitivity tests, and kurtosis of the distributions is reduced. In this hypothetical situation, because of the correlations structure in the Bayes Net, uncertainty distributions relating to the selected Baskets' percentage changes and monetary costs may be more Gaussian if future Meat prices were better constrained. However, the opposite may apply with other foods or combinations of foods; only detailed sensitivity testing will reveal how the estimates might change under different circumstances.

Gaining new or better information about individual contributory food price trajectories can radically change inferences about target item forecast distributions; focussing only on mean values can conceal or distract from meaningful effects, especially in patterns of tail behaviour. And, when we are dealing with multiple variables – here just ten food categories – the interplay

between factors and their uncertainties will be almost impossible to evaluate reliably, without the assistance of suitable numerical software. There are always likely to be subtle, sometimes counter-intuitive, relationships lurking in the complexities of any stochastic model of this nature.

The changes in the CPI Basket sub-food costs, expressed as percentages, appear numerically smaller than the corresponding changes if monetary costs are used. This is likely due to the weights that are applied to the different ways the various Baskets are composed. For instance, under the scenario of No deal and Meat price being set to its 95th percentile, the increase in mean percentage change for the CPI Basket is less than a factor 2x (+44% -v- +24%), whereas the monetary cost increase is more than a factor 3x greater (£44.84 -v- £13.97). Similar differences in sensitivity are present at the distribution 95th percentile levels.

Basic dependence structuring is added into the net to reflect the likely main correlations in prices that exist in reality. The converse, and frequently adopted, assumption -- that probabilistic model parameters are independent when in fact they are correlated -- introduces the risk of under-estimating the extent of joint distribution tails. At this stage, we do not claim that the correlation structures included in our Bayes Net model are comprehensive or numerically precise -- formally determining these dependencies requires further work. This said, the influence links in our net (i.e. arrows carrying correlation values) serve to offset, to some extent, the fallacious assumption that all the food price factors can be regarded as independent of one another.

To map the entire landscape of various possible Brexit-related food price projections, a full range of similar what-if sensitivity tests would be needed, including compound pricing evaluations for multiple combinations of foodstuffs, and for all, jointly; such an exercise lies outside the scope of the present analysis.

For peer review only
